# Putting the Squeeze on Compression Garments: Current Evidence and Recommendations for Future Research: A Systematic Scoping Review

**DOI:** 10.1007/s40279-021-01604-9

**Published:** 2021-12-06

**Authors:** Jonathon Weakley, James Broatch, Shane O’Riordan, Matthew Morrison, Nirav Maniar, Shona L. Halson

**Affiliations:** 1 School of Behavioural and Health Sciences, Australian Catholic University, 1100 Nudgee Rd, Banyo, Brisbane, QLD Australia; 2 Sports Performance, Recovery, Injury and New Technologies (SPRINT) Research Centre, Australian Catholic University, Fitzroy, VIC Australia; 3 Carnegie Applied Rugby Research (CARR) Centre, Institute for Sport, Physical Activity and Leisure, Leeds Beckett University, Leeds, West Yorkshire UK; 4 Institute for Health and Sport (iHeS), Victoria University, Footscray, VIC Australia; 5 Australia Institute of Sport, Bruce, ACT Australia; 6 School of Behavioural and Health Sciences, Australian Catholic University, Fitzroy, Melbourne, VIC Australia

## Abstract

**Background:**

Compression garments are regularly worn during exercise to improve physical performance, mitigate fatigue responses, and enhance recovery. However, evidence for their efficacy is varied and the methodological approaches and outcome measures used within the scientific literature are diverse.

**Objectives:**

The aim of this scoping review is to provide a comprehensive overview of the effects of compression garments on commonly assessed outcome measures in response to exercise, including: performance, biomechanical, neuromuscular, cardiovascular, cardiorespiratory, muscle damage, thermoregulatory, and perceptual responses.

**Methods:**

A systematic search of electronic databases (PubMed, SPORTDiscus, Web of Science and CINAHL Complete) was performed from the earliest record to 27 December, 2020.

**Results:**

In total, 183 studies were identified for qualitative analysis with the following breakdown: performance and muscle function outcomes: 115 studies (63%), biomechanical and neuromuscular: 59 (32%), blood and saliva markers: 85 (46%), cardiovascular: 76 (42%), cardiorespiratory: 39 (21%), thermoregulatory: 19 (10%) and perceptual: 98 (54%). Approximately 85% (*n* = 156) of studies were published between 2010 and 2020.

**Conclusions:**

Evidence is equivocal as to whether garments improve physical performance, with little evidence supporting improvements in kinetic or kinematic outcomes. Compression likely reduces muscle oscillatory properties and has a positive effect on sensorimotor systems. Findings suggest potential increases in arterial blood flow; however, it is unlikely that compression garments meaningfully change metabolic responses, blood pressure, heart rate, and cardiorespiratory measures. Compression garments increase localised skin temperature and may reduce perceptions of muscle soreness and pain following exercise; however, rating of perceived exertion during exercise is likely unchanged. It is unlikely that compression garments negatively influence exercise-related outcomes. Future research should assess wearer belief in compression garments, report pressure ranges at multiple sites as well as garment material, and finally examine individual responses and varying compression coverage areas.

**Supplementary Information:**

The online version contains supplementary material available at 10.1007/s40279-021-01604-9.

## Key Points

**Table:** 

In the past decade, there has been substantial growth in the amount of compression garment research. The majority of this research has occurred in non-professional adult male participants.
The evidence is equivocal whether compression garments improve physical performance. Furthermore, a range of measures (e.g. rating of perceived exertion, kinetic and kinematic outputs and cardiorespiratory measures) are likely unchanged with their use. However, muscle oscillatory properties, perceived muscle soreness and localised skin temperature may be affected.
Despite evidence suggesting there is little benefit of wearing compression garments, it is unlikely that they harm performance. Additionally, wearing compression garments during certain situations (e.g. exercising in cold temperatures) may be of benefit.
When completing future research, researchers should measure and report wearer belief, pressure and materials, and individual responses to compression garment use.

## Introduction

The use of compression garments during and following exercise has become increasingly popular over the last three decades. Simply, compression garments provide mechanical pressure to the body, which may have physiological, biomechanical, performance, and perceptual benefits for individuals exercising [1–5]. There has been considerable attention given to these garments for their potential ergogenic effects on performance and recovery, with athletes commonly wearing them during competition or in the hours and days following exercise. However, a wide range of mechanisms for their potential efficacy have been provided [6]. With the growing interest in compression garments, there is still ambiguity concerning their potential benefits, the research methodologies applied, and how practitioners should prescribe their use. Therefore, a scoping review that considers the entire area would provide practitioners and researchers with an overview of the current scientific literature and help support future research.

The inconsistencies in compression garment and exercise outcomes observed throughout the scientific literature are likely due to a number of methodological reasons [6]. For example, research varies according to when the garments are worn (e.g. during exercise and/or recovery) [7–9], the garment pressure and distribution [10–12], where compression is applied (e.g. upper body [torso and/or arms] vs lower body [knee/thigh/full length]) [13, 14], the level of competition at which the athlete/participant competes [12, 15, 16], the type of exercise performed [7, 15], and the athlete’s belief in the product [2, 17]. Furthermore, considerations such as garment pressure have been estimated to be reported in only one third of the literature to date [6]. This suggests that important methodological information that may influence outcomes is often overlooked. Additionally, while some high-quality reviews have been conducted in the area, they typically have a narrow focus (e.g. high-intensity exercise [18], running performance and recovery [19], recovery from exercise-induced muscle damage [20, 21] or central haemodynamics [1]). Therefore, it is often outside the scope of these reviews to provide an overview of the wider knowledge base of compression garments and exercise and present recommendations for the improvement of future research. Thus, a comprehensive overview of the effects of compression garments and commonly assessed outcome measures (i.e. performance, biomechanical and neuromuscular, cardiovascular, cardiorespiratory, muscle damage, thermoregulatory, and perceptual) relevant to athlete performance will support researchers and practitioners by providing potential benefits and recommendations for use. Additionally, by completing a systematic scoping review, the compression garment and exercise literature can be assessed as a whole, which can provide greater context.

While there has been substantial growth in compression garment use during and following exercise, there is uncertainty regarding their effects [18, 22]. Furthermore, the broad application of their use has caused ambiguity in the literature. Therefore, it is important to provide both researchers and practitioners with an overview of the effects across all relevant performance domains, current evidence base and associated outcome measures, and recommendations for future research. Considering these points, the aims of this scoping review are to (1) conduct a systematic search of the published literature and describe the effects of compression garments on responses to exercise; (2) provide a summary of the research outcomes and variables investigated during compression garment research; (3) describe the characteristics of the research and identify gaps in the literature; and (4) provide recommendations for future compression garment research.

## Methods

### Design and Search Strategy

A systematic scoping review was carried out in accordance with the Preferred Reporting Items for Systematic reviews and Meta-analyses extension for Scoping Reviews (PRISMA-ScR) [23] and was registered with the Open Science Framework (registration: osf.io/p75 × 8). A systematic search of electronic databases (PubMed, SPORTDiscus, Web of Science and CINAHL Complete) was performed from the earliest record to 1 June, 2020. An updated search of the literature was completed on 27 December, 2020. All study designs were included. The search strategy is shown in Table 1. Reference lists of selected articles were manually searched for other potentially eligible papers. Once all articles were found, to support dissemination of findings similar to other systematic scoping reviews, outcome variables from all studies were separated into topics.

**Table 1:** Search terms used in the search strategy

Term 1	Term 2	Term 3
Compression Garments OR Compression Stocking OR Compression Tights	Exercise OR Recovery OR Sport	Muscle Damage OR Perceptual Responses OR Perceived Fatigue OR RPE OR Perceived Recovery OR Muscle Soreness OR Blood Flow OR Biomechanics OR Kinematics OR Muscle Oscillation OR Injury OR Proprioception OR Cardiovascular OR Temperature OR Strength OR Power OR Travel OR Venous Thromboembolism OR VTE OR Perfusion OR Vibration OR Fatigue OR Joint Position

Search terms 1, 2 and 3 were combined with ‘AND’

### Study Selection

Following the removal of duplicates, search results were screened independently by two researchers (JW and MM) against the eligibility criteria. Disagreements were resolved through discussion or via a third researcher (SH). Articles that could not be eliminated by the title or abstract were retrieved and evaluated for inclusion via a full-text review. The titles and authors were not masked to the reviewers.

Studies were eligible for inclusion if they investigated the use of compression garments in able-bodied individuals during exercise or recovery from physical exercise. Additionally, studies were included if they investigated the use of compression garments during activities that related to exercise (e.g. landing, jumping). Only original research investigations in English that were published in peer-reviewed journals were included. Studies were excluded from the review if they did not investigate the effects of compression garments in relation to exercise, or if they investigated the effects of a ‘brace’ for posture or rehabilitation purposes. For example, Cole et al. [24] investigated whether a scapular stabilization brace acutely alters posture and scapular muscle activity in healthy overhead athletes. While these studies do investigate garments that provide compression, stabilisation braces have a distinctly different purpose to commonly used compression garments. Additionally, studies that did not directly investigate the effects of compression garments (e.g. compression garments were worn during exercise but not investigated) were excluded. Review articles and conference proceedings were excluded. Finally, when authors could not be contacted to retrieve a full text of the article, the study was excluded.

### Data Extraction

Following the screening of articles and extraction of information, the authors discussed the overarching themes and topics covered within the compression garment literature. Outcomes from each study were then categorised into topics, similar to previous scoping reviews in sport science [25, 26]. In the current review, these topics were: performance and muscle function, biomechanical and neuromuscular, blood and saliva markers, cardiorespiratory, cardiovascular and haemodynamics, thermoregulatory, and perceptual responses. If an investigation had outcome measures that branched across multiple areas, this study was placed into multiple tables to support the direct comparison of the outcome. It should be noted that if an outcome measure was assessed but was only used to describe participant characteristics rather than used as an outcome of the intervention (e.g. *V*O_2max_ of participants in each group [14, 27]), it was not included in the corresponding table and section. The general characteristics (i.e. year of publication, geography, cohort investigated, sample size, compression garment pressure) of each study were extracted. Data relating to the participants’ characteristics (i.e. sex and age), the aim, outcome measures and key findings of each study relating to the purpose of this review were also retrieved. Outcome measures were converted into comparable units where appropriate (e.g. stature converted from m to cm).

### Data Synthesis

As the aims of a scoping review are to (1) demonstrate the extent, range and nature of the literature on a topic and (2) summarise the findings of the topic [23], no analysis was carried out. Study characteristics, key outcomes and data are summarised with data presented as mean ± standard deviation unless otherwise stated.

## Results

### Search and Selection of Studies

**Fig. 1:**
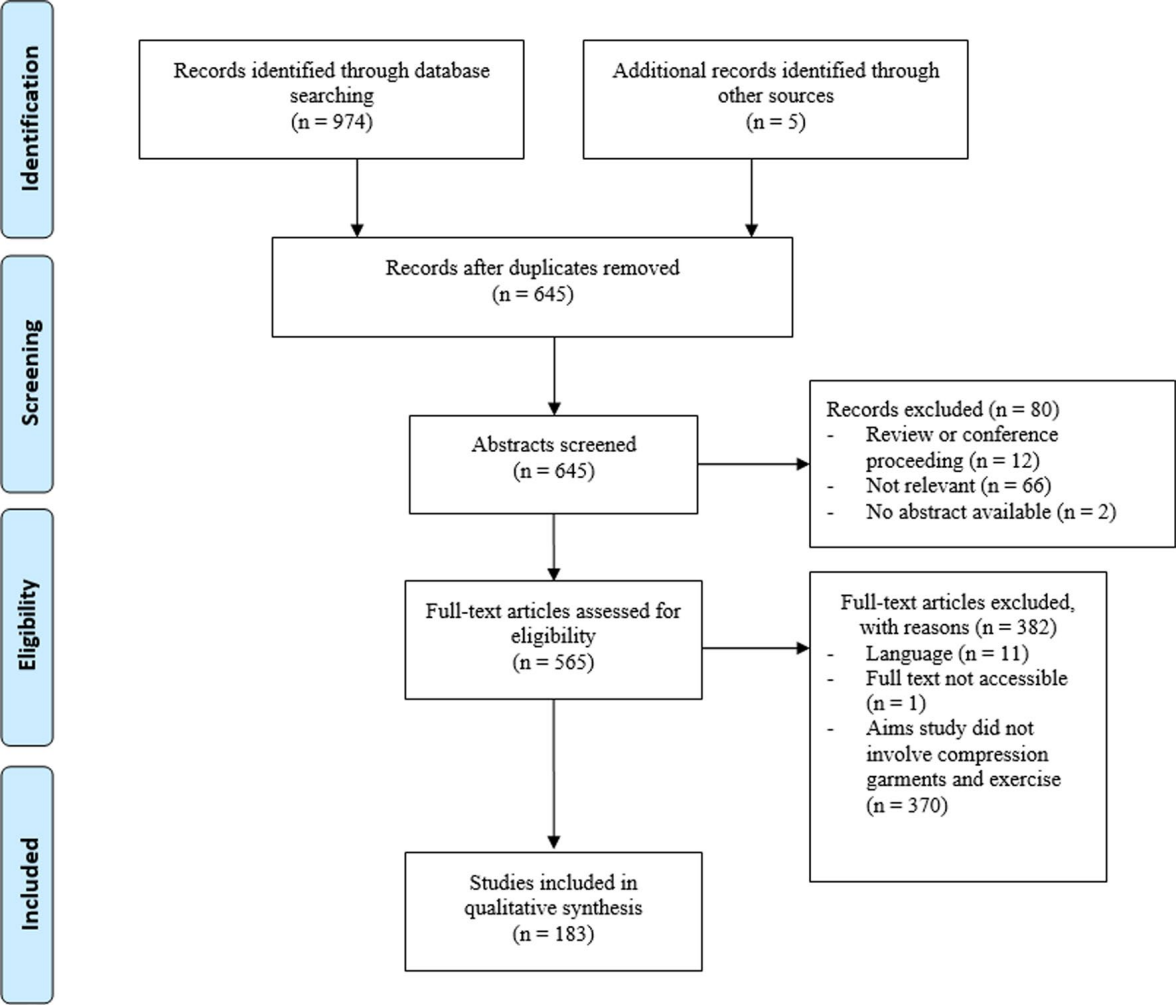
Flow of selection process for eligible studies for inclusion

The literature search identified 974 articles, with five studies identified through a manual search of article reference lists. Following the removal of duplicates and screening of abstracts, 645 full-text articles were assessed for eligibility, with 183 articles included in the qualitative synthesis. The flow of articles through identification to final inclusion is shown in Fig. 1.

### General Study Characteristics

#### Publication Year and Compression Garment Topics

**Fig. 2:**
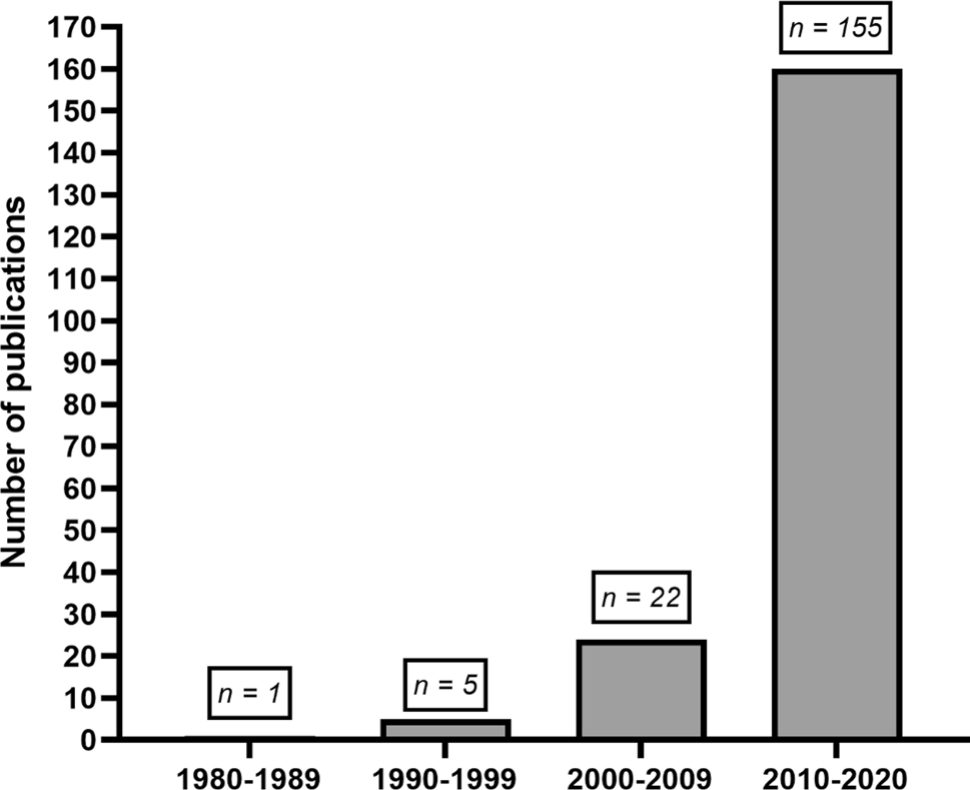
Number of articles by decade of compression garment and exercise research from earliest records until December 2020

Figure 2 demonstrates the substantial growth in studies over recent years, with ~ 85% (*n* = 156) of studies published between 2010 and 2020. The earliest study that investigated compression garment use and physical exercise was from 1987 [28], while all other studies were from 1995 onwards.

**Fig. 3:**
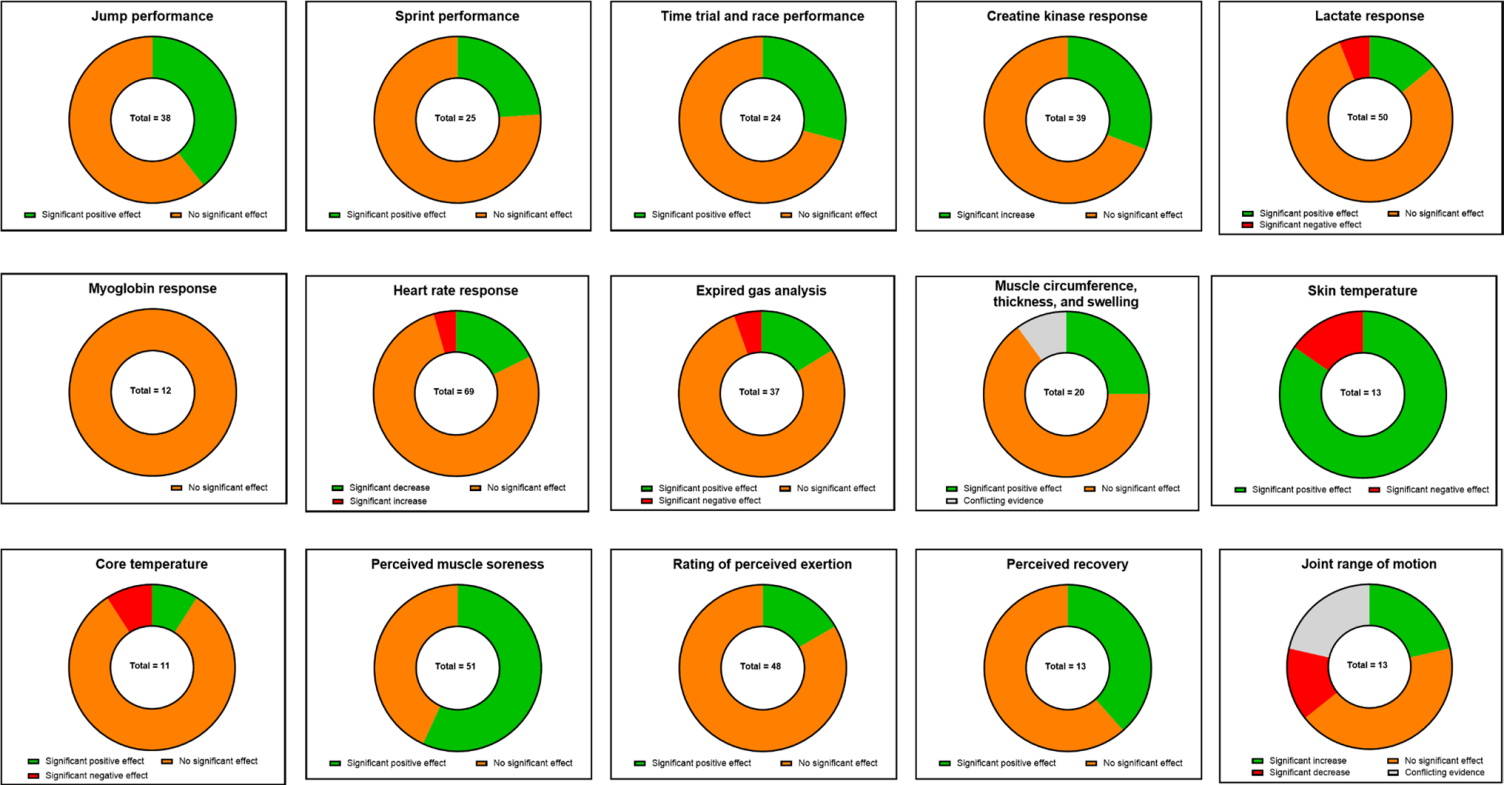
Commonly used outcome measures and reported significant, non-significant, and conflicting findings

From the 183 studies included in the review, performance and muscle function outcomes were assessed in 114 studies (62%), biomechanical and neuromuscular outcomes were assessed in 59 (32%), blood and saliva markers were assessed in 85 (46%), cardiovascular outcomes were assessed in 76 (42%), cardiorespiratory outcomes were assessed in 39 (21%), muscle damage and swelling in 25 (14%), thermoregulatory outcomes were assessed in 19 (10%), and perceptual outcomes were assessed in 98 (54%). This demonstrates the substantial variance across the domains of interest within the compression garment literature. To illustrate the broad research aims and outcomes, a category breakdown by decade can be found in Table 2. Additionally, significant positive/negative or conflicting findings to commonly used outcome measures (i.e. outcome measures that were used 10 or more times) are presented in Fig. 3. For ease of interpretation, measures that use similar methodology (e.g. gas analysis outcomes) or imply similar outcomes (e.g. change in thickness and swelling) have been grouped together.

**Table 2:** Number of articles that had output measures that related to the eight topics identified within the compression garment and exercise literature

Research topic	Year group published				
	< 1990	1990–9	2000–9	2010–December 2020	Total
	Number of investigations	Number of investigations	Number of investigations	Number of investigations	Number of investigations
Performance and muscle function	1	4	17	93	115
Biomechanical and neuromuscular	–	3	8	48	59
Blood and saliva markers	1	–	13	71	85
Cardiovascular and haemodynamic	–	–	8	66	74
Cardiorespiratory	1	–	4	32	37
Muscle damage and swelling	–	1	5	19	25
Thermoregulation	–	–	4	15	19
Perceptual	–	2	13	84	99

If a manuscript had multiple measures within the same category, this was still counted as a single article. Additionally, an article could have multiple outcome measures (e.g. body temperature and *V*O_2max_)

#### Geography of Studies

**Fig. 4:**
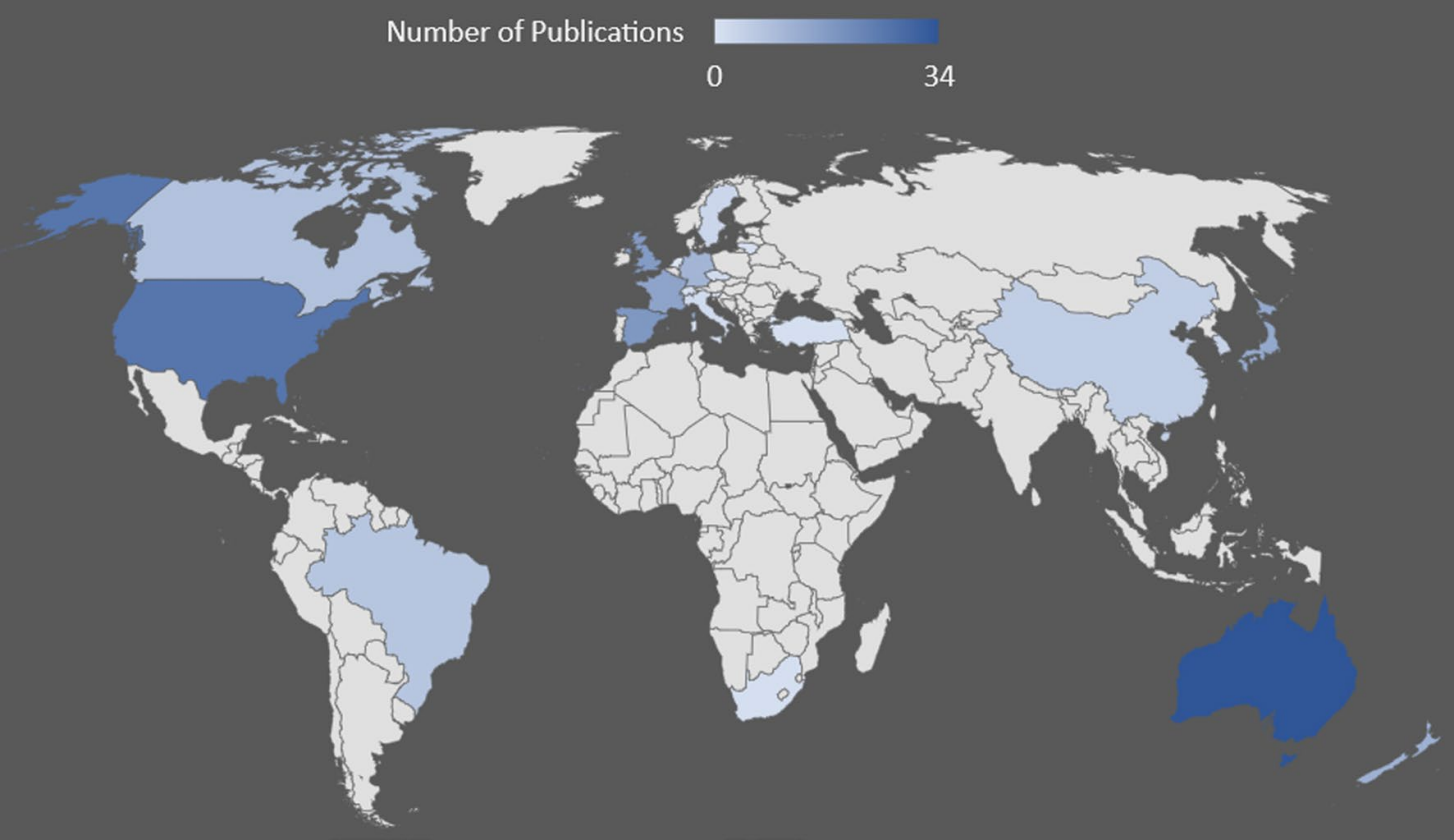
Heat map of articles published by each country. Articles are attributed to the first affiliation of the first author

Of the 183 articles within this review, researchers from Australia have published the greatest number (*n* = 34), followed by the USA (*n* = 26), Spain (*n* = 18), the UK (*n* = 17), France (*n* = 15), Japan (*n* = 14), New Zealand (*n* = 12), Germany (*n* = 11), Canada (*n* = 8), Brazil (*n* = 7), China (*n* = 5), South Korea (*n* = 4), Sweden (*n* = 3), Czech Republic and Italy (*n* = 2), and Lithuania, Slovenia, South Africa, Switzerland, and Turkey (all *n* = 1). Figure 4 provides a graphical illustration of the articles by country.

#### Cohorts Investigated

The majority of studies within this review investigated participants who were non-professional athletes (*n* = 175, 96%), while professional athletes (i.e. an athlete who earns their full time wage through their sport) were used in *n* = 8 (4%) [29–36]. Of the 183 studies included, 51 investigated mixed-sex cohorts, 110 investigated male participants only, and only 14 investigated female participants exclusively. Furthermore, eight studies did not state the sex of the participants [2, 58, 62, 87, 93, 102, 130, 188]. Finally, 167 studies involved adults with a mean reported age between 20 and 64 years, two studies investigated ‘older adults’ (i.e. participants with a mean age > 65 years), 11 studies investigated adolescents (i.e. participants with a mean age < 20 years) [13, 37–46], two studies investigated adult and adolescent cohorts [33, 47], and a single study did not provide the age of the participants included [48].

#### Compression Garment Types

**Fig. 5:**
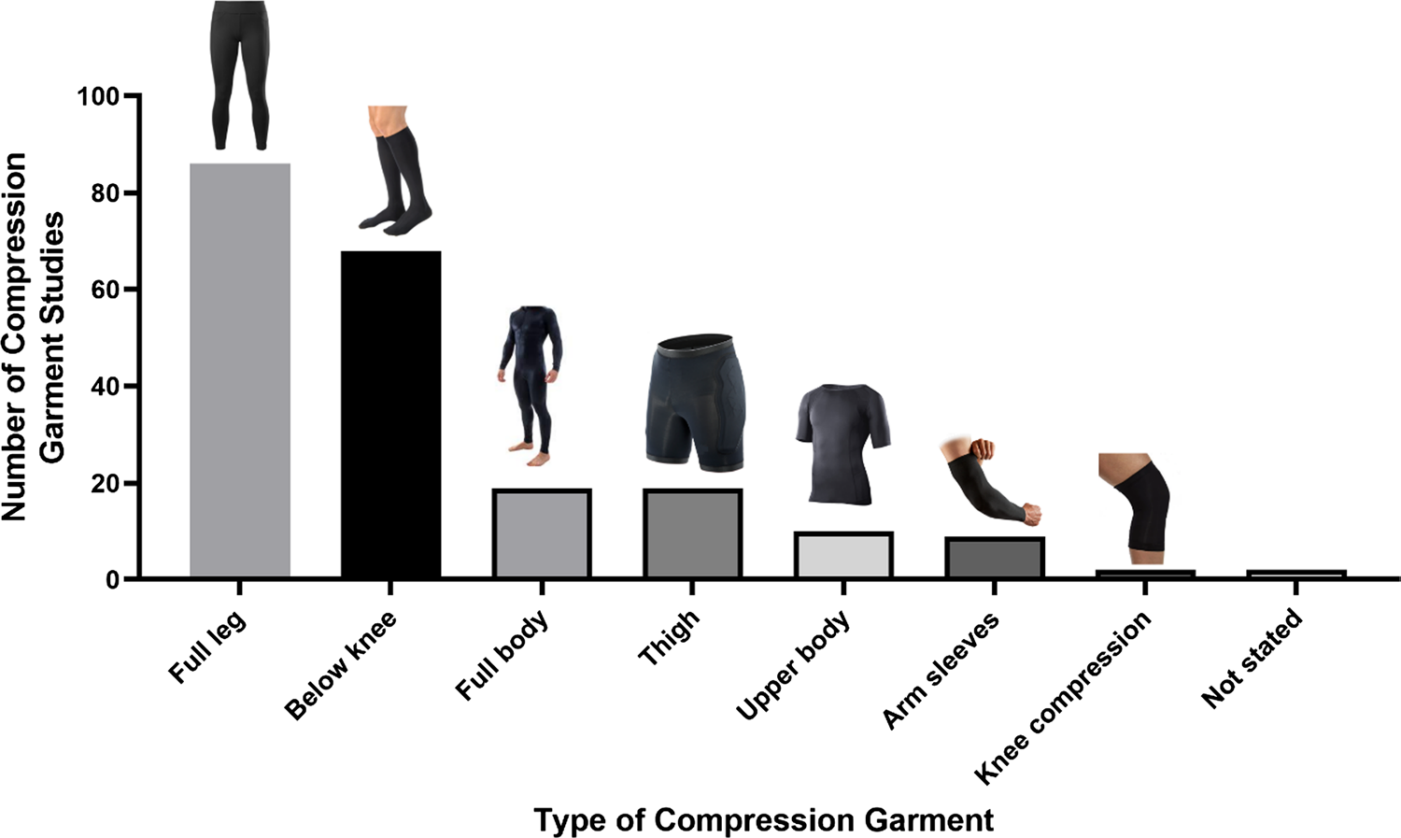
Coverage area of compression garments investigated within the compression garment and exercise literature

The coverage areas of compression garments used in exercise literature are presented in Fig. 5. With multiple garments within the same study, the effects of compression on the lower limbs have been the most investigated (*n* = 175, 81%). Furthermore, upper body compression garments were examined in 19 (9%) studies, with full-body compression garments investigated also in 19 (9%) studies.

## Discussion

**Fig. 6:**
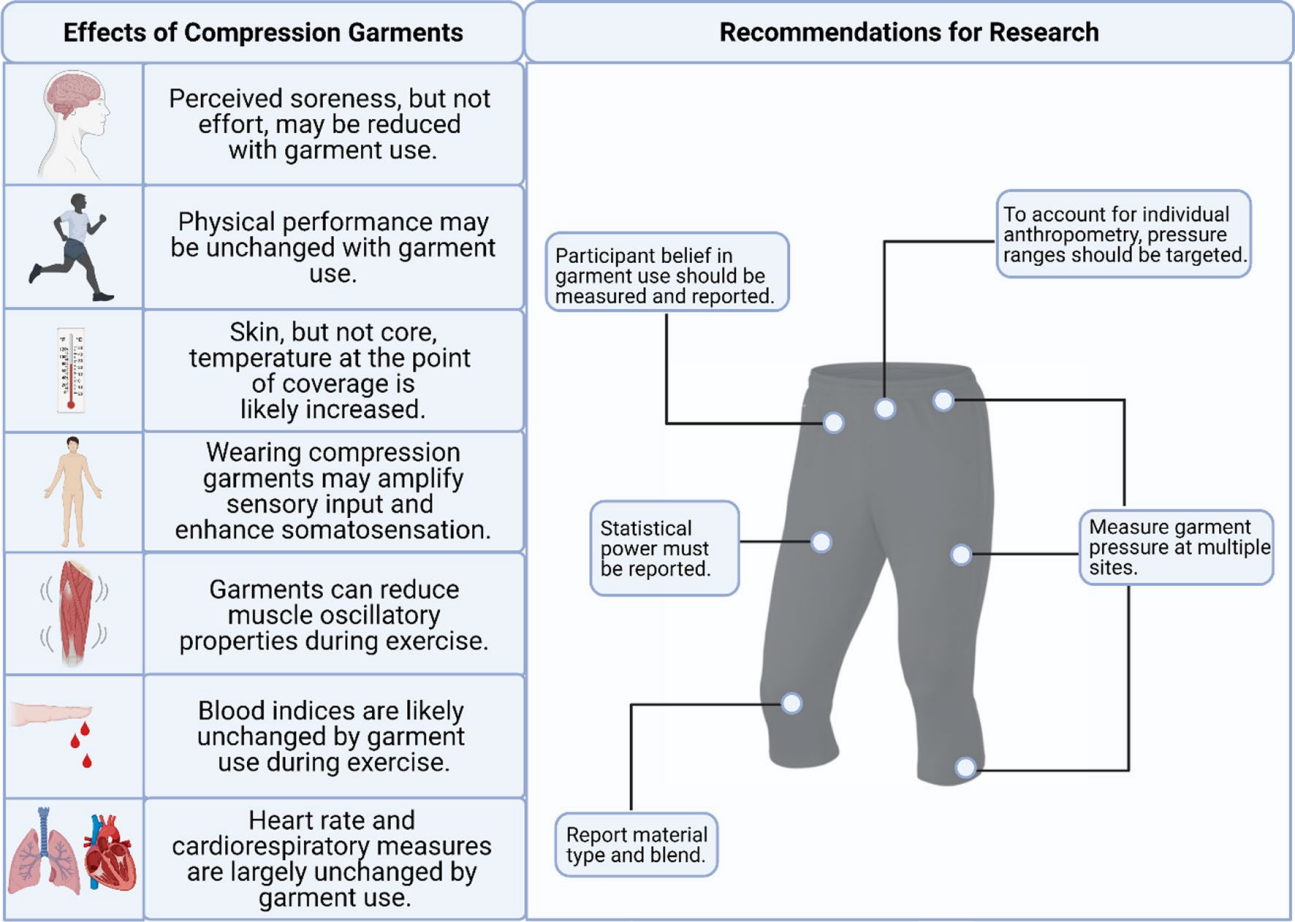
Research recommendations and a summary of key findings relating to the use of compression garments

The aims of this study were to (1) conduct a systematic search of the published literature considering the effects of compression garments and exercise; (2) provide a summary of the research outcomes and variables investigated during compression garment research; (3) describe the characteristics of the research and identify gaps; and (4) provide recommendations for the improvement of future compression garment research. From the 183 studies published, outcome measures have been categorised into performance and muscle function, biomechanical and neuromuscular, blood markers, cardiovascular and haemodynamic, cardiorespiratory, muscle swelling and damage, thermoregulatory, and perceptual measures. Findings throughout the literature are conflicting, with compression garments predominantly causing trivial or small beneficial effects during or following exercise (Fig. 6). However, it is unlikely that compression garments negatively influence exercise-related outcomes. Evidence is equivocal as to whether garments improve physical performance, with little evidence supporting improvements in kinetic or kinematic outcomes. However, compression likely reduces muscle oscillatory properties and has a positive effect on sensorimotor systems, which may explain the reported beneficial effects on joint proprioception and repositioning sense. Further, potential increases in arterial blood flow may occur. Alternatively, it is unlikely that compression garments meaningfully alter metabolic responses, while blood pressure, heart rate, and cardiorespiratory measures are largely unchanged with their use. Compression garments do increase localised skin temperature and can reduce perceptions of muscle soreness and pain following exercise. However, during exercise, rating of perceived exertion (RPE) is likely unchanged. Globally, these findings suggest that compression garments may have ergogenic benefits in specific settings/circumstances, although practitioners should carefully consider their implementation. Furthermore, it is highly unlikely that compression garments have a detrimental effect on exercise and recovery, suggesting that the potential ergogenic benefits outweigh any associated risks.

### Performance and Muscle Function

One-hundred and fifteen studies investigated the effects of compression garments on exercise performance and/or muscle function outcomes (Table S1 of the Electronic Supplementary Material [ESM]). The most common measures investigated were jump (36%, *n* = 41), sprint (22%, *n* = 25) and time trial (21%, *n* = 24) performance. A smaller portion of studies investigated performance metrics including agility (8%, *n* = 9), time to exhaustion (6%, *n* = 7), incremental exercise test performance (6%, *n* = 7), and distance travelled and speed (4%, *n* = 5). In addition, six studies investigated sports-specific performance metrics [2, 15, 31, 40, 49, 50]. A large number of studies (40%, *n* = 46) investigated the effects of compression on metrics related to muscle function, including isometric strength [4, 11, 27, 34, 45, 51–73], isokinetic strength at various contraction speeds [40, 41, 54, 74–87], repetition maximum testing [3, 66, 78, 88, 89], and ballistic movements [40, 57, 87, 90, 91].

The vast majority of studies reported no ergogenic effect of compression on jump [7, 10, 29, 33, 34, 38, 44, 51, 56, 64, 65, 70, 71, 79, 80, 88–90, 92–98], time trial/race [36, 56, 95, 99–112], sprint [10, 34, 38–40, 44, 50, 70, 71, 87–89, 92, 96, 97, 113–116], or agility [10, 38, 44, 88] performance. Nonetheless, there were a number of studies reporting beneficial effects on jump [3, 27, 31, 39, 69, 73, 85, 86, 91, 103, 117–122], time trial/race [5, 17, 72, 123–126], sprint [22, 73, 126–129], and agility [73, 130, 131] performance. A similar pattern was identified with time to exhaustion/fatigue, as compression is reported to improve [8, 132] or have no effect [9, 12, 36, 133, 134] on time to fatigue. Incremental exercise test performance was generally improved when wearing compression garments [28, 100, 131, 135]; however, compression was also reported to have no effect [97] or diminish [47] incremental exercise test performance, highlighting the uncertainty of the effect of compression garments on incremental exercise performance. Compression was reported to improve running speed and distance travelled during netball [92] and team-sport [136] specific circuits, but have no effect on triathlon discipline speed [93] or distance travelled during a simulated soccer match [98]. When investigating sport-specific performance metrics, compression improved tennis shot rate and accuracy [31], but had no effect on throwing [50], kayaking [49], cross-country ski ergometer double-poling [15], or rugby scrum power [40] performance.

The most commonly used markers of muscle function were isometric [4, 11, 27, 34, 45, 51–71] and isokinetic [40, 41, 54, 74–87] muscle strength. Despite this, the effects of compression on these strength markers are unclear, as a large number of studies reported either a beneficial effect [4, 27, 34, 41, 45, 66–73, 82–86] or no effect [11, 40, 51–65, 74–81, 87]. Research investigating the effects of compression on ballistic movements is scarce, with reports of a beneficial effect on bench throw [90], but no effect on bench press barbell velocity [57] or plyometric bounds [87]. Similarly, compression has been reported to have a beneficial effect [3, 66], or no effect [78, 88, 89], on repetition maximum testing.

Of the 115 studies identified, only one study reported a detrimental effect of compression on performance [47]. Considering this represents a very small percentage (~ 1%) of studies across a wide range of performance metrics, these findings suggest it is highly unlikely that wearing compression garments will negatively influence performance and/or muscle function. Additionally, it may improve performance in certain situations. However, considering the large heterogeneity in performance outcomes between studies, future research may wish to consider adopting ‘universal’ performance metrics (e.g. muscle strength) that are consistently implemented across studies to help delineate the true effects of compression on performance and muscle function. Furthermore, there is a continued need to identify when compression garments may be of the greatest benefit. Regardless, the potential ergogenic benefit of wearing compression garments far outweigh the risks of a potentially detrimental effect concerning performance and/or muscle function.

### Biomechanical and Neuromuscular Outcomes

Fifty-nine studies investigated the effects of compression garments on biomechanical and neuromuscular outcomes (Table S2 of the ESM). Studies used a range of participants from recreationally active [53, 118, 137–144] to elite-level athletes [39, 51, 145, 146]. A large portion of these studies (49%, *n* = 29) investigated the effects of compression garments on various kinematic variables including gait [52, 95, 104, 127, 132, 145, 147] and jumping technique [46, 48, 118, 137], limb/muscle oscillatory patterns [39, 48, 54, 121, 143, 148], range of motion [39, 42, 46, 77, 84, 88, 149, 150], and joint angles [16, 46, 48, 51, 118, 127, 141, 147, 151–153]. Thirteen studies investigated the effects of wearing compression garments on kinetic variables including ground reaction forces during landing [39, 48, 55, 93, 137, 140, 145, 154], leg spring stiffness [104, 137, 145], joint power [132, 147], and joint moments [46, 118, 147]. Neuromuscular parameters were investigated in 33 studies, which comprised predominantly outcomes relating to muscle activation patterns as measured by surface electromyography (*n* = 20, 34%) [54, 55, 58–60, 63, 68, 75, 76, 82, 127, 140, 142, 143, 146–148, 152, 155, 156]. Additionally, studies have investigated the effects of compression garments on joint proprioception [4, 53, 121, 144, 149, 157, 158], balance and postural control [138, 140], stability and lower body joint alignment [16], potentiated recruitment patterns and M-wave characteristics using electrical stimulation (*n* = 5, 8%) [59, 87, 159, 160], reaction time [73], skeletal muscle metabolic state during exercise [161] and movement-related cortical patterns via electroencephalography [130].

The large majority of studies investigating kinematic outcomes relating to gait (i.e. step length, step frequency, center of gravity, and/or ground contact time) or jumping (i.e. joint angular velocity, flight/loading/contact durations, and/or vertical displacement of the center of mass) reported no effect of compression garments [52, 70, 95, 104, 132, 137, 145, 147, 152]. Only four studies reported beneficial effects of compression on kinematic outcomes, including increased step length [127], decreased contact time/increased aerial time [104], and reduced tibial acceleration [152] during running, as well as improved kicking kinematics in football [42]. Joint range of motion appears to be largely unchanged when compression garments are worn [16, 39, 42, 46, 77, 88, 147, 149, 150]; however, some studies have also reported increases [48, 83, 84] and decreases [39, 46, 48, 149, 153] in joint range of motion. Multiple studies have reported beneficial effects of compression on joint angles during exercise [51, 118, 127, 141], which have been hypothesized to reduce the risk of injury during landing [118, 127, 141]. However, considering compression garments have also been reported to have no effect on joint angles during exercise [16, 46, 118, 147], future research to delineate these differences is warranted. Compression is consistently reported to reduce muscle oscillatory properties during exercise [39, 51, 121, 143, 148]; however, one study has reported compression to have no effect on joint oscillatory properties during drop jumps [48]. Evidence to support the alteration of kinetics with compression is limited, with a large majority of studies showing no effect on ground reaction forces during landing [48, 55, 93, 137, 140, 145], leg stiffness [137, 145], joint power [132, 147], or joint moments [46, 118]. However, some studies have reported beneficial effects of compression to decrease impact force on landing [39], increase force on landing in anterior-cruciate ligament recovery (indicating an increased reliance on the knee) [154], increased leg stiffness [104], and beneficial effects on joint moments during landing [48] and running [147].

A large number of studies reported compression to reduce muscle activation during different contraction/exercise models [54, 55, 75, 76, 142, 143, 148, 155], which has previously been associated with improved contraction efficiency and movement economy [54, 142]. However, a number of studies also reported no effect [58–60, 63, 68, 140, 146, 147, 156] or an increase [82, 127] in muscle activation with compression, highlighting the need for more research in this area. Compression was also reported to improve the filtering of ‘non-specific’ sensory information [155], which may help to explain the compression-induced reported improvements in joint proprioception and repositioning sense [53, 121, 144, 149, 157, 158], as well as balance and postural sway [16, 138]. There is also evidence to suggest that compression garments positively influence potentiated muscle fiber recruitment [52, 159] and movement-related cortical patterns [130]; however, research in this area is scarce.

### Blood and Salivary Outcomes

Eighty-five studies investigated the effects of compression garments on blood and saliva measures (Table S3 of the ESM). The most common measures investigated were lactate (*n* = 49, 58%), creatine kinase (*n* = 39, 46%), inflammation (e.g. c-reactive protein, interleukins, tumour necrosis factor-α; *n* = 14, 16%), myoglobin (*n* = 13, 15%) and lactate dehydrogenase (*n* = 7, 8%). Other frequent blood markers assessed included glucose (*n* = 5, 6%), blood pH (*n* = 6, 7%), oxygen saturation of haemoglobin (*n* = 5, 6%), markers of coagulation and fibrinolysis (*n* = 4, 5%) and partial pressure of oxygen (*n* = 3, 4%).

A large number of studies (*n* = 40) reported compression to have no effect on lactate, [7, 13, 17, 22, 31, 35, 36, 40, 41, 47, 49, 50, 56, 57, 61, 63, 64, 87, 89, 92, 94, 100, 103, 108–110, 114–116, 119, 120, 123, 126, 127, 129, 132, 133, 135, 136, 162], while seven studies [5, 15, 28, 124, 163–165] have reported positive effects of compression (i.e. reduced blood lactate concentration), and three studies [165–167] have reported negative effects. A similar pattern was identified in blood markers used to assess muscle damage. Creatine kinase was largely not influenced by compression, with 27 studies [5, 7, 11, 27, 30, 33, 40, 44, 61, 67, 69, 74, 79, 85–89, 93–95, 119, 120, 126, 150, 168, 169] reporting no effect, and only 12 studies [32, 38, 50, 65, 71–73, 84, 90, 96, 97, 170] reporting positive effects. Similarly, 12 studies [5, 7, 44, 64, 66, 69, 86, 88, 89, 93, 95, 119] reported no effect of compression on myoglobin, and three studies [64, 73, 170] reported a positive effect. Lactate dehydrogenase was not influenced by compression in five studies [11, 38, 84, 95, 168], while two studies [90, 97] reported positive effects. Of the 14 studies investigating blood markers of inflammation, 13 studies [11, 27, 43, 61, 64, 66, 67, 69, 87, 94, 96, 120, 168] reported no effect of compression, and only one study [119] reported a positive effect on interleukin-6. No studies reported negative effects of compression on blood markers of muscle damage and inflammation.

A small number of studies investigated other blood markers including glucose [64, 89, 93, 119, 120], blood pH [15, 22, 50, 87, 164, 171], oxygen saturation of haemoglobin [12, 15, 50, 93, 167], and partial pressure of oxygen [12, 15, 50], with no effect of compression reported, with the exception of a positive effect in one study measuring oxygen saturation of haemoglobin [167]. For markers of coagulation and fibrinolysis two studies reported no effect [122, 169], and one study [170] reported a positive effect on thrombin–antithrombin complex and plasminogen activator inhibitor with compression. However, one study [172] reported a negative effect of compression on d-dimer. Finally, there is evidence to suggest compression garments positively influence blood markers of glutamic oxalacetic transaminase [97], glutamic–pyruvic transaminase [97], fatty-acid binding protein [112] and biological antioxidant potential [162], and salivary cortisol [37]. However, compression garments appear to have little effect on a range of other blood and salivary markers [30, 43, 66, 73, 90, 93, 95]. Future research should aim to identify the influence of when compression is applied (during or post-exercise), and the duration of use on blood and salivary markers. In addition, further research that investigates the effects of compression on markers of coagulation and fibrinolysis during travel in athletes may be beneficial [171].

### Cardiovascular and Haemodynamic Outcomes

The results from the 74 studies reporting cardiovascular and haemodynamic measures are provided in Table S4 of the ESM. The most common central haemodynamic measures investigated were heart rate (*n* = 68, 91%), blood pressure (*n* = 9, 12%), cardiac output (*n* = 5, 7%), stroke volume (*n* = 4, 5%), and mean arterial pressure (*n* = 4, 5%). Additionally, studies have investigated the effects of compression on heart rate variability [13, 173, 174], total peripheral resistance [106, 173], electrocardiography [165, 175], baroreflex sensitivity [173] and total vascular conductance [176]. Peripheral haemodynamic measures assessed while wearing compression garments included changes in muscle oxygenation (*n* = 19, 25%), arterial blood flow (*n* = 11, 14%) and venous blood flow (*n* = 2, 3%).

The vast majority of studies (*n* = 53) reported no effect of compression on heart rate [2, 5, 7–9, 13–15, 27, 35, 47, 49, 50, 52, 56, 64, 87, 92, 94, 97, 99–108, 114, 115, 117, 123, 124, 126, 127, 131, 135, 136, 148, 156, 162, 163, 165, 171, 177–183], while 12 studies [22, 31, 36, 119, 122, 132, 133, 164, 173, 175, 176, 184] reported positive effects of compression, and three studies [14, 166, 185] reported negative effects. Blood pressure was largely not influenced by compression, with seven studies [106, 107, 123, 156, 162, 165, 186] reporting no change and only two studies [122, 173] reporting a positive effect. There is some evidence to suggest compression garments positively influence cardiac output, with three studies [106, 173, 176] reporting positive effects of compression and two studies reporting no effect [15, 156]. For stroke volume, three studies [15, 106, 156] reported no effect of compression, while only one study [173] reported a positive effect of compression. No studies reported negative effects of compression on blood pressure, cardiac output or stroke volume. Compression literature investigating changes in mean arterial pressure [109, 176, 178, 183] and total vascular conductance [176] has reported no effect of compression. Additionally, there is mixed evidence on the effects of compression garments on other cardiovascular measures. For instance, positive effects of compression have been reported on heart rate variability [173, 174], total peripheral resistance [173], electrocardiography [175] and baroreflex sensitivity [173]. However, other studies measuring heart rate variability [13], total peripheral resistance [106] and electrocardiography [165, 175] have reported no effect of compression.

Muscle oxygenation, often used to reflect changes in peripheral blood flow, was largely not influenced by compression, with ten studies [15, 22, 49, 56, 100, 104, 107, 108, 127, 187] reporting no change and seven studies [9, 104, 122, 133, 136, 156, 188] reporting a positive effect. In addition, two studies [35, 186] reported negative effects of compression on muscle oxygenation. Of the 11 studies investigating arterial blood flow, six studies [22, 104, 106, 162, 176, 178, 184] reported positive effects of compression, while three studies [107, 109, 156] reported no effect and one study [189] reported a negative effect of compression. Research is limited in the effects of compression on venous blood flow with only two studies available to date, with conflicting findings of positive [107] and no effects [190] of compression reported. As alterations in peripheral blood flow (i.e. muscle and venous blood flow) are proposed to be a major contributor to the effectiveness of compression for exercise performance and recovery, future research should incorporate these measures to help assert the underlying mechanisms of compression.

### Cardiorespiratory

Thirty-seven studies investigated cardiorespiratory outcomes related to compression garments (refer to Table S5 of the ESM). The majority of studies involved running (*n* = 23, 62%) [7, 12, 17, 28, 47, 52, 64, 102, 115, 119, 127, 132, 133, 135, 143, 145, 147, 162, 164, 166, 177, 180, 181] or cycling (*n* = 10, 27%) [5, 22, 100, 109, 123, 165, 171, 178, 183, 185] as the primary form of exercise. However, activities relating to alpine skiing (i.e. double-poling and holding a tuck position) [15, 35], kayaking (*n* = 1, 3%) [49] and speed skating (*n* = 1, 3%) [108] were also investigated. Additionally, there were a range of different tests that were used to induce changes in cardiorespiratory responses whilst wearing compression garments. These involved tests to exhaustion (*n* = 7, 19%) [12, 28, 132, 133, 135, 165, 181], tests that were sub-maximal (*n* = 15, 41%) [7, 35, 52, 64, 119, 143, 145, 147, 162, 164, 171, 178, 180, 183, 185], and distance or time trials (*n* = 9, 24%) [15, 17, 22, 47, 102, 108, 115, 123, 127]. Furthermore, six studies [5, 49, 100, 109, 166, 177] used a combination of tests (e.g. a sub-maximal test followed by a maximal test). Finally, 28 studies assessed the effects of compression garments compared to a control in cardiorespiratory changes, while 11 studies directly compared the effects of different types of compression garments.

Of the 37 studies that investigated the effects of cardiorespiratory responses when wearing compression garments, 29 demonstrated non-significant or trivial responses [5, 7, 12, 15, 17, 22, 28, 35, 47, 49, 64, 100, 102, 108, 109, 115, 119, 123, 127, 135, 143, 147, 162, 165, 171, 178, 180, 181, 183], two potentially negative outcomes [166, 185], while six studies [52, 132, 133, 145, 164, 177] suggested positive improvements. From these six studies, it was demonstrated that improved running economy may occur at low sub-maximal intensities (e.g. 8–10 km/h) but not necessarily at higher intensities [133, 177]. Furthermore, during downhill running, there may be small reductions in the respiratory exchange ratio of trail runners [52]. While the majority of studies found little change in the respiratory exchange ratio, it is feasible that changes may be individualised and related to gait variability [145]. Additionally, while changes in cardiorespiratory measures may not change, improvements in performance may occur [5]. Finally, it should be noted that in hot (i.e. 40 °C) temperatures, upper-body compression garments can increase respiratory strain, which may have ramifications on subsequent recovery [166, 185]. Considering the current literature, it is likely that compression garments, irrespective of coverage area or pressure, do not substantially alter cardiorespiratory measures. However, future research may wish to consider individualised responses to their use and whether subsequent alterations in movement strategy as a consequence of their implementation [35] may change cardiorespiratory outcomes.

### Muscle Damage and Swelling

Twenty-five studies have investigated the effect of compression on muscle swelling and other measures of non-blood marker muscle damage (Table S6 of the ESM). The most common measures investigated were changes in muscle circumference, thickness or volume (*n* = 20, 80%), with no effect of compression reported in 13 studies [38, 43, 62, 64, 66, 77, 80, 88, 95, 120, 150, 163, 187], five studies [71, 84, 97, 110, 122] reporting positive effects and two studies [90, 111] demonstrating conflicting outcomes when using compression garments. No studies reported negative effects on muscle circumference or volume. There is evidence to suggest compression garments positively influence muscle damage, with methods including magnetic resonance imaging [161, 191], P-magnetic resonance spectra [192] and histological markers of muscle damage [193] reporting positive effects of compression. Other studies including the use of Doppler echo intensity [60], muscle pennation angle [111] and magnetic resonance imaging [150] report no effect of compression on muscle damage. Future research is encouraged to use more advanced techniques (i.e. magnetic resonance imaging, histological markers) to determine the effect of compression garments on muscle damage and standardise muscle damaging protocols so that direct comparisons can be improved. In addition, research is required to identify if an optimal duration of compression use post-exercise exists to elicit positive effects on muscle damage.

### Thermoregulation

Nineteen studies have investigated thermoregulatory outcomes in response to the use of compression garments during, or following, exercise (Table S7 of the ESM). Exercise included running (*n* = 6, 32%) [40, 107, 115, 166, 180, 194], cycling (*n* = 8, 42%) [14, 106, 123, 171, 178, 183–185], a half ironman (*n* = 1, 5%) [93] and motorised treadmill cross-country skiing (*n* = 1, 5%) [36]. Additionally, two studies [39, 50] completed a range of exercises (i.e. sprinting, jumping, cycling and throwing) and a single study [63] completed drop jumps until fatigue. Only one of the studies that have investigated thermoregulatory responses and compression garments have used professional athletes (Olympic and international medallists [36]), with all other participants being non-professional. However, one study did investigate male older adults (mean age 66 ± 2 years) [183]. Finally, studies investigated the effects of compression garments on skin (*n* = 13, 68%) [39, 40, 50, 63, 106, 107, 115, 171, 178, 180, 183, 184, 194], and core [measured through rectal, tympanic, intestine, and oesophageal temperatures] (*n* = 11, 58%) temperature [14, 93, 106, 107, 115, 166, 171, 178, 183, 185, 194], and thermal comfort and sensation (*n* = 1) [36].

Findings demonstrate that compression garments increase skin temperature at the point of coverage [106, 184], although these changes do not influence core body temperature [166, 178], sweat rate [123, 178, 183] or body mass loss [40, 93, 115, 194]. The use of compression garments increases skin temperature faster than control conditions during warm-ups [39], and these temperatures are maintained to a greater extent throughout, and following, exercise [40, 50, 63, 106, 107, 115, 180, 184]. However, athletes may not perceive differences in temperature compared to loose fitting garments [36]. At cold (i.e. 10 °C) environmental temperatures, compression garments may promote greater skin temperatures, although there is conflicting evidence in hot (32–40 °C) environmental temperatures [14, 185, 194], while sweat wicking compression garments may improve heat reduction following exercise [171]. Finally, it is unlikely that differing full-body compression garment brands, which exert similar pressure, exhibit different thermal effects during high-intensity intermittent exercise [50]. Future research may wish to investigate whether changes in pressure or textile influence thermoregulatory responses at rest, during, and following exercise.

### Perceptual Measures

Ninety-nine studies investigated the effects of compression garments on perceptual measures (Table S8 of the ESM). Studies used both non-professional and professional athletes. The most common measures investigated were muscle soreness (*n* = 51, 52%) and RPE (*n* = 46, 47%). A smaller number of studies investigated perceived fatigue (*n* = 13, 13%), perceived recovery (*n* = 7, 7%), muscle pain (*n* = 9, 9%) and t hermal sensation (*n* = 5, 5%).

Of the 50 studies investigating muscle soreness, 29 reported positive effects of compression on soreness [2, 10, 11, 27, 31, 38, 40, 44, 50, 52, 65–67, 69, 73, 79, 85, 86, 89, 94, 99, 105, 110, 122, 126, 160, 167, 168, 182], while 22 reported no effect [7, 12, 43, 53, 60, 62, 69, 77, 80, 88, 90, 94, 104, 110, 114, 115, 119, 120, 125, 128, 150, 192, 195]. Rating of perceived exertion was largely not influenced by compression, with 40 studies reporting no change [2, 8, 9, 12–15, 47, 49, 50, 56, 69, 94, 95, 97, 99, 101–103, 106–110, 113, 115, 119, 124, 127, 131, 132, 148, 152, 161, 162, 165, 177, 181, 183] and only eight studies reporting improved perception of effort while wearing compression garments [29, 30, 114, 117, 191, 194, 195]. No studies reported negative effects on muscle soreness or RPE.

Consistent with the above findings, of the nine studies investigating muscle pain, six reported a reduction [81, 84, 95, 103, 104, 124], two reported no effect of compression [33, 70] and one reported increased perception of pain when wearing compression garments during a 56-km ultramarathon [111]. Perceived fatigue was not influenced by wearing compression garments in eight of 13 studies [104, 110, 119, 120, 139, 179, 180, 195], while five studies reported improved perceptions of fatigue [10, 29, 44, 122, 126]. Perceived recovery as measured by a visual analogue scale, Total Quality of Recovery Scale or Acute Recovery and Stress Scale was not influenced by compression in six of nine studies [17, 96, 97, 110, 129, 131] and improved in only three of nine studies [29, 61, 94]. Additionally, Profile of Mood States (POMS) [37], perception of performance [121], and perception of muscle oscillation were altered in a single study [196]. Finally, perceived thermal sensation was not affected by wearing compression garments in three of five studies [101, 106, 107] and reduced in two studies where upper-body compression was worn while exercising in the heat [14, 183]. Considering these findings, wearing compression garments may positively influence muscle soreness and muscle pain in the days following exercise. Furthermore, compression likely has little effect on RPE, perceived fatigue, perceived recovery and thermal sensation. These findings suggest it is highly unlikely that wearing compression garments will negatively influence perceptions of effort, recovery, fatigue, and soreness.

#### Gaps in the Literature and Future Recommendations

There is considerable breadth in the exercise and compression garment research, and this review has identified several gaps in the current knowledge. First, despite the strength of evidence that is currently available surrounding compression garments’ influence on sensorimotor systems, the mechanisms and individual responses to their use are still unknown. Second, considering the substantial reduction in muscle oscillation in response to garment use, the implications of this finding have scarcely been investigated. Of note, whether there is a reduction in metabolic cost during exercise is still unknown. Third, while compression garments have demonstrated sufficient evidence to suggest a potential reduction in perceived soreness and pain following exercise, the types of exercise that this is most beneficial for has not been considered. With equivocal outcomes, it is feasible that garment use may have particular benefits following certain forms of exercise. Fourth, with compelling evidence demonstrating compression garments increase localised skin temperature, further research is still required to elucidate the influence of this across a range of environmental conditions. Fifth, while acute responses are commonly assessed, the long-term effects of compression garments are still unknown across all research areas. While acute changes often imply adaptive outcomes or chronic changes, until this research is performed, their long-term effects remain undetermined. Finally, the compression literature to date has had a particular emphasis on the lower body with > 80% using garments that are worn below the waist. Consequently, future research may wish to place greater emphasis on the differing effects of coverage area during, and in response to, exercise.

To improve research quality and transparency, and to enhance research dissemination, several recommendations can be made. First, participant belief in the garment being tested should be assessed and reported. Furthermore, a placebo condition should be implemented where possible. Research has indicated that an athlete’s prior belief in the ability for compression garments to aid performance may enhance the observed outcome [2, 17]. Thus, to truly quantify the expected benefit of these ergogenic aids, it is important to assess their perceived utility [110]. Second, with the potential for garment material and pressure to alter the response to exercise, all compression garment research is recommended to measure and report the pressure across multiple sites and state the material used. This can be further enhanced by aiming for targeted levels of compression at each site to account for differences in individual anthropometry. Last, because of the often subtle effects of these garments, appropriate statistical methods that consider power and the likely observed effect must be completed prior to investigation. Over 90% of the literature within this review did not state this fundamental methodological consideration and, as a consequence, a large portion of the literature is likely underpowered and provides findings that do not allow confident conclusions to be drawn.

## Conclusions

This scoping review has identified 183 studies that have investigated the effects of compression garments on exercise-related outcomes, with a marked increase in research in recent years. The majority of research originates from Australia and has been performed in non-professional male adults. Across the eight identified topics, performance and muscle function measures have been the most investigated, followed by perceptual, and blood and salivary outcomes. While there is a lack of evidence to suggest that compression garments alter kinetic and kinematic outputs, changes in muscle oscillatory properties likely occur with their use. Furthermore, biomechanical and neuromuscular responses are largely unchanged but improvements in joint proprioception and repositioning may occur due to enhanced filtering of ‘non-specific’ sensory information. Despite the lack of consistent and clear evidence supporting compression garment use on cardiovascular, cardiorespiratory, and muscle damage and swelling measures, it should be noted that compression garments can increase skin temperature at the point of coverage, improve heat maintenance during and following exercise, and improve perceptions of muscle soreness and pain in the days following exercise. Finally, the majority of studies reported no ergogenic effect of compression garments on jump, time trial/race performance, strength, sprinting, or agility. Additionally, sport-specific outcomes (e.g. distance covered during competition) have been equivocal. However, it should also be noted that the research to date does not suggest that compression garments have a negative effect on performance. Therefore, athlete preference, belief, environmental considerations, and ergonomics may be the deciding factors whether they are worn during competition.

While the breadth of research that considers compression garments is considerable and this is often accompanied by conflicting findings, there are clear recommendations and directions that researchers should follow to enhance future research. First, researchers must be conscious that the effectiveness of compression garments is likely influenced by wearer belief and this should be assessed and reported. Second, pressure ranges that account for heterogeneity in anthropometry should be provided and these should be provided at multiple sites to support the standardisation of garment use. Additionally, garment material and blends should be reported with the average pressure exerted at each site. Finally, future research should continue to assess whether compression garments have an augmented effect during different forms of exercise and with different coverage areas, assess whether long-term use can alter adaptive outcomes, assess the individual responses associated with garment use and consider their use across a greater breadth of environmental conditions.

## Supplementary Information

Below is the link to the electronic supplementary material.

Supplementary file1 (DOCX 130 kb)

Supplementary file2 (DOCX 65 kb)

Supplementary file3 (DOCX 91 kb)

Supplementary file4 (DOCX 72 kb)

Supplementary file5 (DOCX 49 kb)

Supplementary file6 (DOCX 35 kb)

Supplementary file7 (DOCX 29 kb)

Supplementary file8 (DOCX 96 kb)

## Declarations

### Availability of Data and Material

All data and material reported in this systematic review are from peer-reviewed publications.
