# Supplementary material for: Putting the Squeeze on Compression Garments: Current Evidence and Recommendations for Future Research: A Systematic Scoping Review
Source: Sports Med. 2021 Dec 6;52(5):1141–60. doi: 10.1007/s40279-021-01604-9 (PMC9023423; doi:10.1007/s40279-021-01604-9)
Supplement: Supplementary file 1 — Supplementary file1 (DOCX 130 kb) [file 40279_2021_1604_MOESM1_ESM.docx]

**Supplementary Table S1.** Details of studies and information relevant to performance and muscle function outcomes.

| **Study** | **Cohort/ sample size (n), sex, age** | **Study purpose** | **Outcome Measures** | **Exercise Protocol** | **Compression worn during/after/both** | **Compression pressure – reported value or not stated** | **Key findings** |
| --- | --- | --- | --- | --- | --- | --- | --- |
| Ali and Cressey 2011 | 12 well-trained competitive runners, 3F and 9M, (33 ± 10 y) | Examine the effects of wearing different grades of graduated compression garments on 10-km running performance in well-trained athletes. | 10 km TT and jump height | 10-km running TT | During | Low: 15 and 12 mmHg (calf and ankle), Medium: 21 and 18 mmHg, and High: 32 and 23 mmHg | No effect of compression garments on performance time when compared with a non-GCS control. However, vertical jump height was better maintained from pre to post-exercise when wearing Low and Med compression |
| Ali et al., 2007 | Experiment 1: 14 healthy participants, M, (22 ± 0.4 y)  Experiment 2: 14 healthy participants, M, (23 ± 0.5 y) | To examine the influence of wearing graduated compression stockings on several performance responses during and after exercise. | Field-based running protocol | Experiment 1: two multi-stage intermittent shuttle running tests with 1 h recovery between tests  Experiment 2: continuous 10-km road run | During | 18 – 22 mmHg | No effect on performance |
| Ali et al., 2010 | 10 triathletes, 1F and 9M, (36.0 ± 10.0 y) | To examine the performance effects of wearing graduated compression stockings during fast-paced running in a controlled laboratory environment. | CMJ height and peak power | 90% of 10 km personal best speed at 1% incline for 40 min on treadmill | During | Low compression garment:  Calf: 12 mmHg  Ankle: 15 mmHg  High compression garment:  Calf: 23 mmHg Ankle: 32 mmHg | No effect on muscle function |
| Areces et al., 2015 | 34 marathon runners, 4F and 30M,  Control group: (42.7 ± 7.8 y), Compression group: (41.2 ± 8.9 y) | Investigate the benefits obtained by wearing graduated compression stockings on running pace and maintenance of muscle performance during a real marathon race. | Race time, running pace, jump height, and mean muscle power | Marathon race | During | The highest pressure was at the foot and the malleolus and it decreased proximally, from 25 mmHg to 20 mmHg | No effect of compression on performance and muscle function |
| Argus et al., 2013 | 11 highly trained cyclists, M, (31 ± 6 y) | To evaluate the use of 3 recovery strategies compared with a passive control on repeated sprint-cycling performance in highly trained cyclists | Mean 30-s power output during sprint test | 3 maximal 30 s sprint separated by 20 min of recovery with compression garment | Between maximal sprints | Calf: 27 ± 6 mmHg  Upper thigh: 18 ± 2 mmHg | Compression improved recovery when there is only a short turnaround time between high-intensity cycling bouts |
| Armstrong et al., 2015 | 33 moderately trained runners, 10F and 23M, (38.5 ± 7.2 y) | To determine if lower limb compression garments influence functional recovery from distance running | TTE | Marathon race | During | Ankle: 30 to 40 mmHg  Calf: 21 to 28 mmHg | Compression improved graduated treadmill TTE two weeks after the event |
| Atkins et al., 2020 | 30 recreational basketball athletes, M, (22.5 ± 4.1 y) | To evaluate the effect of wearing lower-body compression garments following basketball-specific exercise on perceptual ratings of recovery and physical performance | Jump height and vertical impulse, repeated sprint test, and 505 agility test | The Basketball Exercise Simulation Test | Post-exercise for 15 hours | Ankle: 7.5 ± 3 mmHg;  Calf: 10 ± 2.5 mmHg;  Thigh: 8 ± 2 mmHg | Non-significant, trivial or unclear effects were evident for all performance measures between compression garment and control groups |
| Bahnert et al., 2012 | 45 AFL players, M, (23.3 ± 4.2 y) | To investigate the associations between post-game recovery modalities chosen by Australian Football League players and their subsequent physical and perceptual recovery, and game performances throughout a season. | CMJ | AFL match | After | NS | No effect |
| Ballman et al., 2019 | 12 basketball players, M, (20.3 ± 1.37 y) | To examine the effects of wearing lower body compression garments on anaerobic exercise performance | Mean and peak power, anaerobic capacity, total work | 2 x 30 second repeated WAnT | During | Ankle: 15 to 20 mmHg  Thigh: 6 to 10 mmHg | Lower body compression garments enhanced mean power output, anaerobic capacity, and total work during repeated WAnT cycling tests |
| Barwood et al., 2013 | 8 physically active, M, (21 ± 2 y) | To establish the thermal and performance effects of wearing graduated compression garments in a hot environment in contrast to control and sham treatment conditions | 5-km TT times and pacing strategy | 15 min running at 10 to 12 km/h followed by a 5 km TT | During | Compression group:  Calf: 20 ± 3 mmHg  Thigh: 11 ± 2 mmHg  Sham group:  Calf: 17 ± 4 mmHg  Thigh: 10 ± 2 mmHg | No effect on performance |
| Baum et al., 2020 | 12 participants, M, (21.5 ± 1.0 y) | This study investigated the effects of a novel lower-body compression garment on muscular power, strength, and endurance relative to a sham control garment in order to test the efficacy of added resistance in augmenting improvements in performance. | 1RM seated leg press;  Peak power, mean power, and jump height during CMJ | Four weeks of lower-body strength-power resistance training | During | NS | Compression improved lower limb peak and average power output, muscular strength, and jump height than those donning shams |
| Berry and McMurray, 1987 | Experiment 1: 6 college students, M,  (22.5 ± 5.4 y)  Experiment 2: 6 healthy college students, M, (21.4 ± 4.3 y) | The first study was designed to determine the effects of compression garments on TTE during a VO_2max_ test | Incremental exercise test | 15 min at treadmill speed set to elicit a heart rate of 130 beats per min, after which time the grade was increased every 2 min by 2.5% until exhaustion | During | Ankle: 18 mmHg, Calf: 8 mmHg | No effect on performance |
| Bieuzen et al., 2014 | 11 highly trained runners, M, (34.7 ± 9.8 y) | To examine the effect of wearing compression stockings on indices of EIMD during trail-running. Compression stockings were worn either during or after a trail run performed at competition pace by experienced off-road runners | CMJ and MVC using isokinetic dynamometer knee extension | The simulated trail race consisted of 3 laps of 5.2 km in mountainous terrain. Each lap was composed of a climbing segment (2200 m, average gradient 13%) followed by a downhill segment (3000 m, average gradient –9%) | During or after | 25 mmHg during running and 20 mmHg during recovery | The results presented here provide new evidence that wearing compression stockings during running can immediately benefit muscle recovery, as inferred from MVC and CMJ |
| Born et al., 2014 | Sub-study 1:  12 track and team sport athletes, F, (25.0 ± 3.0 y); | Sub-study 1: To assess the effects of compression garments with silicone stripes (which mimic kinesio taping) on repeated sprint performance | Time and power | 30 x 30-m sprints (1 sprint/min) | During | ~ 18 to 20 mmHg across the entire lower body | Performance of the repeated 30 x 30-m sprints was improved during the final third of the protocol. |
| Born et al., 2014 | 10 elite German ice speed skaters, 6F and 4M, (23 ± 7 y) | To investigate whether the application of compression to both legs in elite ice speed skaters improves 3000-m time. | 3000 m time | 3000-m race simulation | During | Thigh: 20.3 ± 2.3 mmHg  Calf: 24.4 ± 3.1 mmHg | Compression provided no effect on during 3000-m ice speed skating. |
| Broatch et al., 2018 | 20 recreationally active participants, 11F (25 ± 2 y), 9M (28 ± 6 y). | This study aims to assess the effects of lower-limb compression garments on exercise performance during a repeated sprint protocol with short rest intervals | Peak power and work (average and within each set) | 4 sets of 10 x 6-s maximal sprints, inter-spaced by 24 s of recovery between bouts and 2 min recovery between sets | During | Thigh: 11.7 ± 2.3 mmHg  Calf: 26.4 ± 6.4 mmHg  Ankle: 21.5 ± 8.2 mmHg | Lower-limb compression garments worn during repeated-sprint exercise improved repeated-sprint performance |
| Broatch et al., 2019 | 12 elite Australian volleyball athletes, F, (25 ± 2 y) | To determine the effects of wearing compression socks during long-haul travel on sports-specific performance, physiological, and haematological alterations in elite female volleyball athletes | CMJ height, mean velocity, relative power, and jump:dip ratio. | Air travel | During | Maximal calf: 23 ± 11 mmHg  Ankle: 19 to 22 (±8) mmHg | Sports compression socks maintained exercise performance when worn during ∼8.5 hours of air travel in elite female athletes |
| Brophy-Williams et al., 2016 | 12 well trained runners, M, (30.5 ± 8.1 y) | To assess the effect of wearing compression socks during a 1-h recovery period following a 5-km running TT on performance in a subsequent 5km TT | Performance decrement | A 5 km TT, a one hour recovery period, then a repeat of the warm-up and 5 km TT | Between time trials | Maximal calf: 23 ± 11mmHg  Upper ankle: 22 ± 8mmHg  Lower ankle: 19 ± 8mmHg | Participant belief in the product played a large role in determining whether a performance benefit occurred. |
| Brophy-Williams et al., 2019 | 12 runners, M, (30.5 ± 8.1 y) | Assess the effect of wearing compression socks during a 5km running TT on performance-based parameters. In addition, the capacity for compression socks to impact subsequent performance was also investigated. | 5 km TT performance | A 5 km TT, a one hour recovery period, then a repeat of the warm-up and 5 km TT | During the first warm up and TT. | Maximal calf: 37 ± 4 mmHg  Upper ankle: 31 ± 4 mmHg  Lower ankle: 23 ± 4 mmHg | Smaller decrement in repeated TT performance |
| Brown et al., 2020 | 45 rugby players, M, Custom-fitted compression group, n=13, (24 ± 6 y), Standard-fitted group n=16, (23 ± 3 y), Control group, n=16, (22 ± 4 y) | To evaluate the effects of CG applying different pressures on muscular recovery after EIMD in rugby players | MVC knee extension, CMJ, 30-m sprint time | 20 × 20-m sprints with 5-m deceleration  100 drop jumps | After | Custom fit garment group  Ankle: 32 ± 3 mmHg  Calf: 24 ± 4 mmHg  Thigh: 19 ± 3 mmHg  Standard size garment group  Ankle 11 ± 5 mmHg  Calf: 10 ±3 mmHg  Thigh: 7 ± 3 mmHg | Custom-fitted compression garments designed to apply higher pressures than commercially available garments were associated with improved strength recovery after EIMD in rugby players. |
| Burden and Glaister 2012 | 10 well-trained triathletes and cyclists, M, (34.6 ± 6.8 y) | To investigate the effects of both ionized and non-ionized compression tights on performance responses to, and short-term recovery from, sprint and endurance cycling | Power output-VO_2_ relationship. 30-s sprint: peak power output, mean power output, and fatigue index | 8 x 3–min stages on a cycle ergometer; 30 s at 150% of VO_2max_ power. Subjects then recovered for 3 min at 40% of VO_2max_ power before performing a maximal 30-s sprint | During | Control: Mid-thigh Anterior: 8 mmHg, Posterior: 6mm Hg. Mid-shank Anterior:11 mmHg, Posterior: 8 mmHg;  Nonionized and ionized: Mid-thigh Anterior: 15 mmHg, Posterior: 11 mmHg. Mid-shank Anterior: 21 mmHg, Posterior: 16 mmHg | Ionized compression tights had no effect on cycling performance of any type. |
| Carling et al., 1995 | 23 participants,  16F and 7M, (26 ± 4 y) | Evaluate the effect of applying external compression on DOMS and the accompanying manifestations of soreness, swelling, ROM, and isokinetic strength for a period of 4 days following induction of DOMS | Maximal elbow flexor force at angular velocity of 120°/sec | 70 eccentric contractions of the non-dominant elbow flexor muscles, opposing the motion of the dynamometer moving at 120˚/sec through 120˚ of motion | After | Sleeve pressure: 17 mmHg | No significant differences were present for either group. |
| Cerqueira et al., 2014 | 13 participants, sex NS, Compression group, n=7 (22 ± 1 y), Control group, n=6, (20 ± 1 y) | To assess the efficacy of compression sleeves worn for short-time periods (12 h), on recovery from the symptoms of EIMD in the upper arm muscles | Measures taken pre-, 24, 48, 72, 96 h after eccentric exercise: isometric strength | 30 repetitions of eccentric dumbbell curls which lasted 4 to 5 s | 12 h following | NS | No significant differences between groups. |
| Chatard et al., 2004 | 12 trained cyclists, M, (63 ± 3 y) | The purpose of this study was to determine whether or not elastic compression stockings can be used successfully by elderly sportsmen to increase performance recovery following maximal exercise. | Maximal power | 2 x all out 5-min cycloergometer separated by 80 min of rest | For 80 min between maximal attempts | Ankle: 44 hPa, Calf: 24 hPa, Mid-thigh: 17 hPa | This study demonstrates that elastic compression stockings have ergogenic properties when worn during an 80-min recovery period by older sportsmen. |
| Chaudhari et al., 2014 | 29 participants, 13F and 16M, (23.4 ± 4.8 y) | To test whether the directional compression afforded by the directional shorts reduces hip adductor activation | MVC and %MVC | 45° run-to-cut manoeuvres | During | NS | No effect on MVC |
| Choi and Hong 2019 | 7 participants, M, (21.4 ± 1.7 y) | To determine the differences in the EMG of the thigh among film-welded compression suits, film-free compression suit and a loose sportswear during isokinetic exercise of the knee. | Muscle fatigue at 60°/s, 180°/s, and 240°/s from the *rectus femoris, vastus lateralis, vastus medialis oblique, semitendinosus,* and *bicep femoris.* | 5 trials at 60°/s, 10 trials at 180°/s, and 30 trials at 240°/s | During | Compression suit pressure: 0.7 to 3.5 kPa  Film-welded suit on the compression suit: 1 to 5.3kPa | Wearing film-welded compression suits decreased muscle fatigue of *vastus lateralis, vastus medialis oblique* at each angular velocity, however, it did not support the fatigue of the hamstrings in almost all conditions of exercise. |
| Dascombe et al., 2011 | 11 well-trained middle distance runners and triathletes, M, (28.4 ± 10.0 y) | To determine the effectiveness of wearing an undersized lower body compression garment on performance parameters relating to endurance running. | 3 x TTE trials | Time to exhaustion test consisting of the participant running at 90% of VO_2max_ velocity until volitional exhaustion | During | Regular size, thigh: 13.7 ± 2.3 mmHg, Calf: 19.2 ± 3.2 mmHg  Undersize, thigh:  15.9 ± 2.6 mmHg, calf: 21.7 ± 4.3 mmHg | There were no significant effects of wearing undersized lower body compression garments on any of the measured performance parameters. |
| Dascombe et al., 2013 | 7 elite flat-water kayakers, 2F (25.0 ± 4.2 y) and 5M (21.8 ± 2.8 y) | To determine the effects of wearing upper body compression garments on selected performance responses during simulated flat-water kayaking in elite kayakers | Kayaking performance | Six-step incremental test and a subsequent 4min performance test on a kayak ergometer | During | NS | The data demonstrated no significant improvements in the selected performance measures during simulated flatwater kayaking in elite kayakers wearing compression |
| Davies et al., 2009 | 7 university netball academy athletes, F, (19.7 ± 0.5 y) | To investigate whether wearing compression tights for 48 h following plyometric exercise would lead to a decrement in sprinting and jumping performance | 5-10-20m sprint time, CMJ height, 505 agility test time | 5 x 20 drop jumps from a platform 60 cm high followed immediately by a maximal upward jump, with a 2-min rest period between sets | For 48 h afterwards | 15 mmHg from the lower to the upper legs | No performance benefit of compression |
| de Glanville and Hamlin 2012 | 14 trained multisport athletes, M, (33.8 ± 6.8 y) | To determine the effects of wearing commercially available graduated compression garments during prolonged recovery (24 h) on subsequent 40-km cycling TT performance in trained multisport athletes. | Mean power output, performance time, and pedal cadence | 40-km cycling performance | Post-exercise for 24 h | Upper ankle: 6.0 ± 2.4 mmHg, Upper calf: 14.7 ± 2.5 mmHg, Upper leg segment: 11.8 ± 2.5 mmH | Relative to a placebo garment, wearing a compression garment for a 24-h recovery period between successive 40-km TT substantially improved the performance in the second TT. |
| Del Coso et al., 2013 | 36 experienced triathletes, sex NS, Control, n=17, (35.8 ± 6.3 y) Compression, n=19, (35.0 ± 5.3 y) | To investigate the potential of compression stockings to preserve muscular performance during a half-ironman competition. | Swimming velocity, cycling velocity, running velocity, jump height, and lower-limb muscle power | Half-iron man | During | NS | The use of compression stockings did not improve total race time or the velocities in the swimming, cycling and running sectors of the triathlon. In addition, the triathletes wearing compression stockings presented similar post-race reductions in jump height and maximal lower-limb muscle power output |
| Doan et al., 2003 | 20 track athletes, 10F (19.2 ± 1.3 y) and 10M (20.0 ± 0.9 y) | To determine how custom-fit compression shorts affect athletic performance and to examine the mechanical properties of the shorts. Specific performance and mechanical tests were designed to assess the effect of the garment on muscle oscillation, jump power, skin temperature, impact absorption and elasticity. | 60-m sprint, max power and jump height during CMJ. | 60-m sprint; Muscle oscillation during CMJ; 60-s cycling intervals | During | NS | Greater vertical jump height was reported with the garment |
| Driller and Halson 2013 | 12 highly trained cyclists, M, (30 ± 6 y) | To investigate the effect of wearing lower body compression garments on performance during a 30-min endurance cycling test in highly trained cyclists. | Mean power | 15 min at 70% peak power, followed immediately by a 15-min TT | During | ~18 mmHg at ankle decreasing to ~10 mmHg at *gluteus maximus* | Wearing lower body compression garments during a 30-min cycling performance test resulted in a significantly higher mean power output and a possible benefit to exercise performance |
| Duffield and Portus, 2007 | 10 club level cricket players, M, (22.1 ± 1.1 y) | To compare the effects of three different types of full-body compression garments and a control condition on performance in intermittent, repeat-sprint and throwing performance in cricket players. | Throwing and repeat-sprint performance | 30 min repeat-sprint exercise protocol comprising 20 m sprints every minute, separated by submaximal exercise. Throwing tests included a pre-exercise and a post-exercise maximal distance test and accuracy throwing tests. | During and 24 h after | NS | Results indicated neither throwing nor repeat-sprint performance was improved by any garment, and minimal differences were evident between garments. |
| Duffield et al., 2008 | 14 rugby players, M, (19 ± 1 y) | To determine whether compression garments improve intermittent-sprint performance and aid performance on consecutive days | 20-m sprint time and peak power, and repeated max scrum efforts peak power | 4 x 15-min quarters of a simulated team game (exercise circuit) repeated across 2 days | During the simulated team games and for ~15 h afterwards | NS | Results indicated no significant differences in intermittent-sprint or explosive power performance on either of the 2 days of the high-intensity, simulated team-sport exercise. |
| Duffield et al., 2010 | 11 team sport athletes, sex NS (20.9 ± 2.7 y) | Examine the effect of wearing compression garments during and 24 h following high-intensity, intermittent-sprint and stretch shortening cycle activities on post-exercise performance and recovery of evoked and voluntary muscle performance. | MVC for knee extension/flexion between 15° and 80° (0° being full extension) at angular velocity of 60°/s. 20-m sprint times, distance travelled in 10 bounds and % decrements | 10-min exercise protocol of a 20-m sprint and 10 plyometric bounds every min | During and for 24 h after | NS | Compression garments had no effect on evoked or voluntary muscle performance during and/or up to 24 h following intermittent-sprint and stretch shortening cycle activity |
| Duffield et al., 2014 | 8 professional tennis players, M, (20.9 ± 3.6) | To investigate the effects of combining cold water immersion, compression garments, and sleep-hygiene recommendations on physical, physiological, and perceptual recovery after 2-a-day on-court training and match-play sessions | Shot volumes/error rate, CMJ height | Each respective on-court session involved 90 minutes of coach-led drills (including a 30-min warm-up) and 90 minutes of competitive match play | Between drill and match play (~3 h) and for 4 h match play | NS | Post-session compression garments exhibited large effects for increased CMJ, stroke rates, and time in play for the ensuing competitive match play. |
| Ehrstrom et al., 2018 | 13 trail runners, M, (38.6 ± 5.7 y) | To examine whether wearing high-pressure compression garments (>15 mmHg) during a 40-min treadmill downhill run on acute and delayed neuromuscular responses and running economy in well-trained trail runners accustomed to eccentric work | MVC | 40-min downhill running at -8.5˚ | During | Middle calf: 20 to 25 mmHg  Upper calf: 18 to 20 mmHg  Middle thigh: 16 to 18 mmHg  Lower thigh: 18 to 20 mmHg | No effect on MVC |
| Erten et al., 2016 | 20 junior athletes, 9F and 11M, (15.6 ± 0.5 y) | To assess the effects of compression stockings and electrostimulation on isokinetic strength values during the first 30 min of recovery | 0, 3rd, 5th, 15th, 30th min MVCs of right plantar flexion, right dorsal flexion, left plantar flexion, and left dorsal flexion | 30 min of treadmill running at 85% of anaerobic threshold | 30 min following exercise | 20 to 30 mmHg | Compression stocking and electrostimulation intervention effects on force generating capacity on the isokinetic device had statistically significant better improvements when compared to passive recovery. |
| Faulkner et al., 2013 | 11 trained runners, M, (23.7 ± 5.7 y) | To examine the effects of lower-limb compression on 400-m run performance | 400 m sprint time (100, 200, 300 and 400 m splits) | 400-m sprint | During | Long garment: 2 to 13 mmHg  Short garment: 4 to 21 mmHg | There were no significant differences in individual 100-m split times between conditions. |
| Ferguson et al., 2014 | 21 participants, M, (21 ± 1 y) | To examine the effects of neuromuscular electrical stimulation, compared to graduated compression socks on muscle strength | MVC of knee extensors and flexors | 2 x 45-min sections of continuous intermittent exercise separated by a 15-min rest period | At least 12 h post- the 1 h testing point | Ankle: 40 mmHg  Calf: 20 mmHg | No differences in the rate of recovery of muscle strength (MVC) |
| French et al., 2008 | 26 participants, M, (24.1 ± 3.2 y) | To evaluate compression garments as a regeneration strategy after EIMD | CMJ and repeat CMJ Height. 10 and 30-m sprint time. "M" agility test, time. 5-RM parallel back squat | 6 x 10 squats with 100% of body mass + 5 second eccentric repetition with the participants 1RM | For 12 h after exercise | Calf: 12 mmHg  Thigh:10 mmHg | Results indicate that compression garments demonstrated no clear benefit of performance enhancement and/or recovery characteristics |
| Fu et al., 2012 | 12 participants, M, (21.2 ± 1.4 y) | To examine the effects of compression levels on muscle strength, EMG and mechanomyography of the *rectus femoris* during isometric and isokinetic muscle actions at both low (60˚/s) and high (300˚/s) angular velocity | Peak torque during the MVC task, peak power, average power for the first five reps, and total work during the isokinetic tasks. The decrease in the peak muscle torque across the 25 repetitions was evaluated | Isometric testing consisted of 2 x 5 s of isometric MVCs of the quadriceps with the knee joint angle fixed at 30˚ of extension and hip joint angle set at approximately 90˚ of flexion. Isokinetic muscle actions at both low (60˚/s) and high (300˚/s) angular velocity | During | Medium compression: 66.4 N  High compression: 85.8 N | The results showed no enhancement effects with either medium or high compression levels on peak torque, peak/average power and work production of the quadriceps femoris |
| Geldenhuys et al., 2019 | 41 marathon runners, 12F and 29M, Compression group, n=20 (34 ± 4.8 y), Control group, n=21 (34± 6.4 y) | To determine the impact of below-knee compression on performance in runners before, during, and after an ultramarathon road race | Race performance | 56-km ultramarathon | During | NS | No effect on race performance |
| Gimenes et al., 2019 | 20 football players, sex NS, Compression group, n=10 (18.3 ± 0.5 y), Control group, n=10 (18.5 ± 0.5) | The effects of using compression stockings on the match-based physical performance indicators, heart rate responses, and perceptual measurements in under-20 soccer players during 2 matches separated by 72 hours | GPS outputs | Two football matches separated by 72 h | During | 20 to 30 mmHg | No effect on locomotor output |
| Goh et al., 2011 | 10 recreational runners, M, (29.0 ± 10.0 y) | To compare the effects of compression garments on running performance at ventilatory threshold and at VO_2max_ velocity in hot (32˚C) compared with cold (10˚C) ambient temperatures. | TTE | Running commenced on the treadmill at a velocity that elicited the subject’s pre-determined ventilatory threshold for 20 min, followed by a run to exhaustion at the subject’s individual VO_2max_ in 10˚C and 32˚C | During | Calf: 13.6 ± 3.4 mmHg  Thigh: 8.6 ± 1.9 mmHg | No performance differences were observed as a result of wearing compression garments in both hot and cold temperatures |
| Goto and Morishima 2014 | 9 participants, M, (21.0 ± 0.4 y) | To investigate the effects of wearing a compression garments for 24 h on the changes in muscular over time after resistance exercise. | 1RM for chest press and MVC for the knee extension | Six exercises for the upper body and three for the lower body muscles. Each exercise set comprised 10 repetitions involving five sets for bilateral leg press and bilateral knee extension and three sets for the remaining seven exercises | Worn for 24 h after resistance training | NS | Wearing a compression garments after strenuous resistance exercise promotes muscular strength recovery of the upper body and lower limb muscles |
| Goto et al., 2017 | 11 participants, M, (22.7 ± 0.9 y) | To determine the effect of compression garments during post-exercise periods after two repeated bouts of exercise on exercise performance | 10 x 6-s cycle ergometer, mean power output with 40% 1RM bench press, CMJ height, repeated jump height contact time and repeated jump index, | Repeated sprint cycling and resistance exercise | During recovery (4 h after sprints and 18 h after resistance exercise) | Thigh: 11.5 ± 0.6 hPa  Calf: 17.6 ± 1.8 hPa | No effect on muscle function |
| Govus et al., 2018 | 32 cross country national and junior level skiers, Seniors  9F (23.2 ± 2.6 y) and  12M (25.2 ± 3.6 y),  Juniors  5F (18.2 ± 0.8 y) and  6M (18.0 ± 0.6 y) | To determine whether or not compression garments and neuromuscular electrical stimulation accelerated the recovery of CMJ height before and 8, 20, 44 and 68 h after a cross-country sprint skiing competition in a cohort of elite senior and junior cross-country skiers. | CMJ | Cross-country sprint skiing competition | For ∼17 h following the post-competition period. | Lower-body compression, ankle: 14.6 ± 0.1 mmHg, achilles tendon: 13.6 ± 1.4 mmHg, calf: 13.7 ± 1.3 mmHg, tibial tuberosity: 7.6 ± 1.1 mmHg, patella: 8.3 ± 2.3 mmHg, mid-thigh: 5.3 ± 1.1 mmHg and 5 cm below crotch: 4.2 ± 2.8 mmHg | No effect |
| Hamlin et al., 2012 | 22 well-trained rugby union players, M, (20.1 ± 2.1 y) | To determine the effects of wearing either a compression garment or a similar-looking placebo garment over a 24-h recovery period on subsequent performance measures in well-trained male rugby union players | Repeated sprint mean and best time, fatigue decrement, 3km time | A series of exercise circuits designed to simulate a game of rugby | After | Compression: Sphyrion: 8.6 ± 2.6 mmHg, mid-calf: 13.4 ± 2.0 mmHg, and mid-trochanterion: and 9.0 ± 2.2 mmHg  Control: Sphyrion: 2.6 ± 1.2 mmHg, mid-calf: 5.0 ± 1.5 mmHg, and mid-trochanterion: 3.5 ± 0.9 mmHg | Wearing a compression garment for 24 h after high-intensity exercise was associated with better repeated sprint and endurance performance. |
| Hettchen et al., 2019 | 19 handball players, M, (31.3 ± 7.7 y) | To determine the effect of compression tights on relevant parameters of recovery applying a conscientious methodological and biometrical approach | Changes of maximum isokinetic hip and leg extensor strength as determined by an isokinetic leg press, and jump height | Two sets of 8-10 repetitions of lunges, unilateral calf raises, and squats were prescribed. Exercise to failure per exercise in the range of 8-10 reps. 60 seconds recovery between exercises | Compression was applied initially for 24 h and then 12 h intermitted by 12 h of non-use for a total of 96 h | 19.0 - 26.2 mmHg for the onset of the calf muscle; 16.3 - 23.5 mmHg for the highest calf circumference; 9.9 - 18.1 mmHg two fingerbreadths beneath the fossa popliteal; 7.7 - 14.3 mmHg at the mid-knee; 9.9 - 13.9 at mid-thigh and 8.0 - 12.2 for the region two fingerbreadths beneath the crotch | A positive effect of compressive tights on performance parameters after resistance type EIMD |
| Higgins et al., 2009 | 9 State league and representative netballers, F, (22.6 ± 4.6 y) | The purpose of this study was to examine effectiveness of compression garments on performance markers in a game-specific circuit for netball. | CMJ - flight times captured at 9th min of each quarter. 20-m sprint at 14th min of each quarter. GPS distances were recorded throughout the circuit | A circuit designed to simulate competitive netball. 4 x 15 min circuit per session to simulate the four quarters of a competitive game. The circuit comprised of six stations simulating game conditions that were repeated throughout each quarter. | During | NS | No significant effect of compression |
| Hill et al., 2014 | 24 recreational marathon runners, 7F and 17M, Compression group (47.7 ± 10.8 y), Sham ultrasound (41.1 ± 10.5 y) | To investigate the effects of wearing a commercially available, lower limb, compression garment on the recovery of strength following a marathon run. | Isometric MVC of knee extensors | Marathon run | For 72 h after exercise | Between 9.9 to 24.4 mmHg | There were no significant differences between groups for MVC |
| Hill et al., 2017 | 45 recreationally active participants, 19F and 26M, Low pressure group  (29.2 ± 4.7 y),  High group (32.7 ± 7.8 y),  Sham group (28.3 ± 4.1 y) | To assess whether garments exerting a higher degree of pressure are more effective in facilitating recovery compared to garments exerting a lower pressure | CMJ and MVC of the knee extensors | The muscle damaging protocol consisted of 100 drop jumps from a 0.6 m platform. Participants performed 5 s of 20 drop jumps, with 10 s between each jump and a 2 min rest period between sets. | For 72 h post exercise | Low: 8.1 ± 1.3 mmHg at the thigh and 14.8 ± 2.1 mmHg at the calf High: 14.8 ± 2.2 mmHg at the thigh and 24.3 ± 3.7 mmHg at the calf | High compression was more effective at improving muscle function than low compression and a sham treatment group. |
| Houghton et al., 2009 | 10 trained amateur field hockey players, M, (21 ± 2 y) | To investigate the effects of compression garments on performance in field hockey | Sprint times | The Loughborough intermittent shuttle test | During | NS | Similar increases in 15 m sprint times from stage 1 to stage 4 in both conditions |
| Jakeman et al., 2010 | 32 participants, F, (21.4 ± 1.7 y) | To determine whether a combined treatment involving sports massage and compression immediately after damaging exercise was an effective strategy to manage the symptoms of EIMD induced by strenuous plyometric exercise | Isokinetic muscle function, CMJ height and squat jump height. MVC knee extensor contractions through 80° ROM from full knee extension, at 60°/s | 10 x 10 plyometric drop jumps from a 0.6-m box. 60 secs rest between sets | For 12 h post-exercise | Calf: 17.3 mmHg  Thigh: 14.9 mmHg | Minimal performance differences between treatments were observed |
| Jakeman et al., 2010 | 17 participants, F, (21.4 ± 1.7 y) | To investigate the efficacy of complete lower limb compression clothing on recovery from the symptoms of EIMD following strenuous plyometric activity | Squat jump height, CMJ height, and isokinetic muscle function | 10 x 10 plyometric drop jumps from a 0.6-m box. 1-min rest between sets | For 12 h post-exercise | Calf: 17.3 mmHg  Thigh: 14.9 mmHg | The compression treatment reduced decrements in CMJ performance, squat jump performance, and knee extensor strength loss. |
| Kemmler et al., 2009 | 21 moderately-trained runners, M, (39.3 ± 10.7 y) | To determine the effect of below-knee stockings with constant compression on selected parameters of running performance in healthy male runners | Speed at aerobic and anaerobic thresholds. | Stepwise-speed incremental exercise test | During | Ankle: 24 mmHg  Calf: 18 to 20 mmHg | Compression stockings were effective for enhancing performance during sub-max and maximal running exercise. |
| Kerhervé et al., 2017 | 14 participants, M, (21.7 ± 3.0 y) | To determine if wearing calf compression sleeves during a prolonged running exercise (∼150 min) performed on trails with marked elevation gain and loss, had a measurable effect performance. | Total run time | 24 km run | During | 23 ± 2 mmHg | No effect on trail running performance |
| Kim et al., 2017 | 16 participants, M, Compression group, n=8 (24.3 ± 1.3 y), Control group, n=8 (23.1 ± 3.8 y) | To investigate how wearing compression garments after eccentric exercise using elbow flexor could alter the recovery of isometric strength. | Maximal isometric strength | Each eccentric muscle contraction was performed for 3 s, according to signals from the investigator, followed by a 12-s rest period. Each participant completed 2 x 25 reps with a 5-min rest period between sets | For 24 h after exercise | 5 to 10 mmHg | Compression garments effectively reduced DOMS and helped the recovery of isometric strength |
| Kraemer et al., 1996 | 36 college volleyball players, 18F (20.4 ± 3.09) and 18M (21.0 ± 3.1) | To determine the influence of compressive shorts on power production during maximal effort vertical jumping. | Mean force and power output | 10 repeated maximal CMJ | During | NS | Undersized compression garments improved mean force in singular CMJ efforts. Both regular and undersized garments improved mean force and power output compared to control. |
| Kraemer et al., 1998 | 20 participants, 10F (25.2 ± 3.8 y) and 10M (23.2 ± 4.8 y) | This study assessed whether opposing compression forces produced by commercially available "compression shorts" affect the repetitive force production capabilities of the thigh muscles during repetitive open- and closed-kinetic-chain exercise tests | Total work, torque across 150 repetitions of knee extension, number of repetitions with 70% of squat 1RM | MVC knee extension and flexion movements for the 3 sets of 50 reps at 180˚/sec.  As many squats as possible with 70% of 1RM | During | NS | Compression shorts do not contribute to any additional fatigue in repetitive high-intensity exercise tasks |
| Kraemer et al., 1998 | 20 athletic participants, 10F (21.3 ± 2.3) and 10M (22.3 ± 4.8y)  20 non-athletic controls, 10F (20.3 ± 2.4) and 10M (21.3 ± 2.3) | The purpose of this study was to determine  whether compression shorts affected vertical jump performance after different fatigue tasks  (i.e., endurance, strength, and power). | CMJ power | Endurance fatigue: 30 min run at 70% maximal heart rate  Strength fatigue: 4 x 10 leg press (10RM)  Power fatigue: 10 x 10 maximal effort jumps | During | NS | The compressive garment significantly enhanced mean power output in the jump test both before and after different fatigue tasks. |
| Kraemer et al., 2001 | 15 non-strength-trained participants, M, Compression group (22.3 ± 2.9 y), Control group (22.1 ± 3.3 y) | To determine whether a compression sleeve worn immediately after maximal eccentric exercise enhances recovery | 1RM elbow flexion at 60°/s | 2 sets of 50 repetitions at 60°/s on an isokinetic dynamometer with 3 min rest between sets. Every fourth repetition, the subject performed a MVIC at end range, followed by an eccentric contraction in which the subject resisted mechanically forced elbow extension | After | NS | Compression promoted recovery of force production. |
| Kraemer et al., 2001 | 20 non-strength-trained participants, F,  Compression sleeve group (21.3 ± 2.9 y),  Control group,  (21.1 ± 3.3 y) | To investigate whether constant compression via the use of a compressive arm sleeve would reduce the severity and duration of soreness associated with DOMS. | Elbow flexion strength via peak torque output at 60°/sec and power output | Dynamometer (60°/s). 2 sets of 50 repetitions with 3 minutes rest between sets. Every fourth repetition, the subject performed a maximal concentric contraction with an isometric hold followed by an eccentric contraction | After | 10 mmHg | Compression was found to promote faster recovery of force production |
| Kraemer et al., 2010 | 20 resistance trained subjects, 9F (23.1 ± 2.2 y) and 11M (23.0 ± 2.9 y) | To evaluate the influence of a whole-body compression garment on recovery from a typical heavy resistance training workout in resistance trained men and women | Movement reaction time: ruler drop - pinch method.  CMJ: peak power, mean power, max performance decrement.  Bench Throw and squat jump: peak force, peak power, peak velocity | 3 sets at 8-10RM of back squats, bench press, stationary lunge, bent-over row, Romanian dead lift, biceps curl, sit up, high pull from hang. | For 24 hours after exercise | NS | Improved bench throw, but no effect on CMJ or squat jump |
| Kraemer et al., 2016 | 19 recreationally-active participants, M, (23.1 ± 2.4 y) | To examine the impact of trans-American jet travel on physical performance and associated hormonal and sleep-related responses to gain insight into potential mechanistic contributions to any reductions in physical performance. Secondarily, to assess the impact of a return flight on recovery processes following a demanding physical activity and whether a compression garment intervention could ameliorate any of the tissue damage upon return to the original time zone. | Handgrip strength, CMJ, pro-agility, 40-yard sprint | Roundtrip trans-American jet travel | During | NS | The compression group demonstrated no significant physical performance differences from baseline testing and also showed significantly better performances than the control group at the corresponding time points. |
| Kumstát et al., 2018 | 8 participants, M, (27.1 ±2.3 y) | To investigate the comparative effect of using compression calf sleeves with active recovery and passive rest on immediate recovery and high intensity repeated cycling performance | Peak power, relative peak power, fatigue index, were evaluated for the 30, 20 and 10 s maximum cycling performance tasks | Three maximal bouts (30 s, 20s, and 10 s; the external loading was set at 7.0% of the individual’s body mass). 3 min recovery between efforts. | For 24 min between maximal effort bouts. | Ankle: 25 mmHg  Calf: 21 mmHg | No effect |
| Lee et al., 2017 | 12 participants, sex NS, (24.0 ± 2.2 y) | To assess the effect of different pressure levels on agility | Agility test | Two side steps | During | Garment 1:  Knees: 0.44 to 0.58 kPa  Thighs: 0.48 to 0.68 kPa  Garment 2:  Knees: 0.95 to 1.03 kPa Thighs: 0.53 to 0.71 kPa  Garment 3:  Knees: 1.67 to 2.12 kPa  Thighs: 0.80 to 1.14 kPa | Wearing compression pants with the highest-pressure level enhanced agility and response time |
| MacRae et al., 2012 | 12 recreationally trained cyclists, M, (26 ± 7 y) | To examine the effects of full-body compression garments on exercise performance. | Time/power/cadence during 6-km TT | 60-min fixed-load cycling at ~65% VO_2max_ and a 6-km TT | During | Correctly-sized group: 11 to 15 mmHg, over-sized group: 8 to 13 mmHg | No effect on performance. |
| Marqués-Jiménez et al., 2017 | 18 semi-professional football players, M, (24.7 ± 4.1 y) | To evaluate the influence of wearing different types of compression garments during matches and recovery after a friendly soccer match | CMJ, 10 and 20 m sprint, t-test, Yo-Yo IR2 at 24, 48 and 72 h post-match. GPS was also measured during the match | Soccer match | During the match and 7 h/day during 3 days post-match (players put them on each day after the testing session). | Stockings: 20–25 mmHg at ankle and 15–20 mmHg at calf  Tights: 25–30 mmHg at calf and 15–20 mmHg at thigh  Shorts: 15–20 mmHg at thigh | No effect |
| Martorelli et al., 2015 | 15 resistance trained participants, M, (23.1 ± 3.9 y) | To examine the effects of upper-body graduated compression sleeves on neuromuscular and metabolic responses during power training | Mean power, peak power, isometric strength, repetitions to failure, and average barbell velocity | Repetitions to failure test were performed with 50% of 1RM. | During | NS | No effect of compression sleeves on neuromuscular performance or metabolic responses. |
| Maton et al., 2006 | 15 participants, sex NS (32 ± 6 y) | To test if elastic compressive stockings increase muscle fatigability during sustained muscle contraction or if it improves recovery after fatigue | Static ankle dorsi-flexion force in the right limb | 3 sets, separated by 30 s of exerting a 50% ankle dorsi-flexion MVC force and to sustain this force level for as long as possible | During | Ankle: 23.6 mmHg  Calf: 14.3 mmHg | No effect of compression on endurance and recovery times |
| McDonnell et al., 2018 | 59 participants, 20F and 39M, (18 to 60 y) | To assess efficacy of two types of compression strategies (knee high graduated compression socks and knee high uniform compression socks) following exercise on functional recovery. | Sit and reach test and ankle flexibility, muscle strength with an isokinetic dynamometer in the hike trial and with a modified force platform in the run and calf exercises | Hike trial and trail run | Worn during the daytime for 4 days, including the day of the DOMS-inducing exercise | A Graduated compression: Ankle: 21 ± 0.7 mmHg, calf: 13.2 ± 1.9 mmHg; Uniform compression: Ankle: 20.5 ± 2.2 mmHg, calf: 21.0 ± 2.4 mmHg | No difference between the uniform and graduated socks |
| McMaster et al., 2017 | 10 well-trained rugby players, M, (21.0 ± 2.6 y) | To determine the effects of wearing a wrestling-style compression suit on repeated vertical CMJ, a horizontal loaded sled push and IMTP performance in well-trained rugby athletes. | IMTP peak force, CMJ peak velocity, 5-m 75-kg sled push time | IMTP peak force, CMJ, 5m 75kg sled push | During | 13 to 31 mmHg | Compression suit had a likely beneficial effect on IMTP (peak force), but no meaningful effect on vertical CMJ (peak velocity) or resisted horizontal sprint (5-m time) performance in well-trained male rugby athletes |
| Ménétrier et al., 2011 | 14 moderately endurance trained, M, (21 y) | To test whether calf compression sleeves improved running performance. | TTE | 15 min at rest, 30 min at 60 % maximal aerobic velocity, 15 min of recovery, a running TTE at 100 % maximal aerobic velocity, and a 30 min recovery period. The two running trials were carried out on a 12 % treadmill slope. | Before, during and after | Medial ankle: 15 mmHg  Gastrocnemius: 27 mmHg | No effect on TTE |
| Miyamoto et al., 2011 | 14 participants, M, (25.6 ± 3.7 y) | To examine the effects of elastic compression stockings on the torque generating capacity of the *triceps surae* muscle and EMG activity | MVC | Calf raises with 15 sets of 10 repetitions with 30 s recovery between sets | During | Ankle: 18 to 30 mmHg | The main finding was that elastic compression stockings had no effect on the decline of MVC torque regardless of the pressure intensity |
| Mizuno et al., 2016 | 18 participants, M, (21.9 ± 0.6 y) | To determine the effects of wearing a lower body compression garment for 24 h following running (either downhill or level) in terms of recovery of exercise performance. | CMJ, rebound jump (i.e., 5 repeated jumps), and drop jump | 30 min of downhill running | 24 h post exercise | Compression group: thigh: 11.5 ± 0.6 hPa, calf:17.6 ± 1.8 hPa  Control group: Thigh: 7.1 ± 1.3 hPa, calf: 11.5 ± 2.1 hPa | Recovery of CMJ height and rebound jump index were significantly improved by compression |
| Mizuno et al., 2017 | 8 participants, M, (23.4 ± 2.4 y) | To investigate the effect of wearing lower body compression garments exerting different pressure levels during prolonged running | CMJ height | 120 min of uphill running at 60% of VO_2max_ | During | High pressure garment: Thigh: 26.9 ± 3.3 mmHg; Calf: 29.2 ± 3.8 mmHg  Medium pressure garment: Thigh: 16.1 ± 2.0 mmHg; Calf: 17.9 ± 3.5 mmHg  Control garment: Thigh: 4.4 ± 1.2 mmHg; Calf: 3.0 ± 1.6 mmHg | The major finding of the present study was that the medium compression trial showed a significantly lower exercise-induced decrease in CMJ height compared with that of the high compression trial. |
| Mizuno et al., 2017 | 30 participants, M, compression thigh group, 10M (21.3 ± 0.4y), compression sock group, 10M (21.6±0.8y), control group, 10M (22.9±0.7y) | Examine the effects of the body coverage area of compression garments on the exercise performances during prolonged running | MVC of knee extension and plantar flexion, CMJ and 60-cm drop jump | 120 min of uphill running at 55% of VO_2max_ | During | Thigh compression group:  14.7± 0.6  Calf compression group:17.4 ± 0.5  Control group: Thigh: 3.0± 0.3 and calf: 1.8 ± 0.2 | The present findings revealed no significant effects of the body coverage area of the compression garments on the selected exercise performances (MVC and CMJ). |
| Montgomery et al., 2008 | 29 basketball players, M, (19.1 ± 2.1 y) | To evaluate the effectiveness of recovery strategies on physical performance during a 3-day tournament style basketball competition | Line drill, 20-m acceleration, agility, and vertical jump | 3-day mini-tournament involving one full 48 min game per day | For ~18 hours post-game | ~18 mmHg | No effect of compression on line-drill, 20-m acceleration, agility, or vertical jump |
| Négyesi et al., 2020 | 24 right-side dominant healthy adults, 12F and 12M, (25.5 ± 4 y) | Examine whether below-knee compression garment reduces fatigue-induced strength loss and join position sense errors in healthy adults. | Peak torque, MVC at 60° and 80° | 100 maximal isokinetic eccentric contractions at 30°/sec with the right-dominant knee extensors | During | NS | Below-knee compression garments reduced fatigue-induced strength loss at 80° knee joint position in healthy younger adults |
| Pavin et al., 2019 | 20 amateur soccer players, F, (20.6 ± 3.9 y) | To evaluate the effect of compression stocking use during an amateur female soccer match on match-induced fatigue indicators | T-test, heel-rise test, YoYoIE2 (48 h before match and post-match) | Soccer match | During | NS | Compression improved agility and lower limb muscular endurance performances following the match. |
| Pearce et al., 2009 | 8 healthy participants, M, (23-27 y) | To (1) Investigate time course changes in strength and visuo-motor tracking to confirm previous findings; (2) examining if sports compression garment are able to assist in the performance of a visuomotor tracking task following a bout of eccentric exercise, resulting in DOMS, at intervals up to 14 days post-exercise. | MVC force in biceps. | 35 maximal isokinetic eccentric extensions at 90˚/s | During | NS | No effect on MVC |
| Pereira et al., 2014 | 24 resistance trained, M, (24.1 ± 5.2 y) | To examine the effects of graduated compression sleeves on muscle performance during isokinetic intermittent high-intensity exercise. | Average torque, work, and power in the preacher curl isokinetic dynamometer | 4 sets of 10 maximal elbow flexion/extension at 120˚/sec. 60 sec separated sets. | During | NS | No effect on isokinetic performance during concentric or eccentric actions. |
| Pereira et al., 2014 | 22 resistance trained participants, M, (24.6 ± 5.1 y) | To examine the effect of graduated compression sleeves worn during exercise on muscle recovery in young resistance trained men. | Isometric peak torque (MVC) | 4 sets of 10 maximal elbow flexion/extension at 120˚/sec. 60 sec separated sets. | During | NS | No effect between groups for isometric peak torque or muscle activation |
| Pruscino et al., 2013 | 8 highly trained hockey players, M, (21.9 ± 2.3 y) | To investigate the efficacy of wearing commercially available, full-length, lower-body compression garments following a hockey-simulation exercise protocol to determine whether this strategy influenced the post-exercise recovery of muscle function in highly trained athletes | 5 rep CMJ and squat jump (vertical displacement and peak force). | Hockey simulation protocol | 24 hours after | Ankle: 19.1 mmHg, calf: 7.2 mmHg, thigh: 4.9 mmHg. | No effect |
| Ravier et al., 2018 | 18 handball players, M, (23.2 ± 5.0 y) | To investigate benefits of wearing full-leg length compression garments during conventional handball-specific circuit exercise on maximal and rapid muscle force characteristics immediately at the end and 24 h post-exercise. | Within-session: sprint performance and CMJ height. Post-session: isometric MVC and RFD | Handball-specific circuit exercise during three 12-min periods. Exercise was separated by four minutes of rest. | During exercise | Ankle: 15, medial gastrocnemius: 27, and vastus lateralis: 14 mmHg | No effect on sprint performance, jump performance, and muscle strength characteristics. However, immediately post-exercise, loss of MVC was attenuated with compression garments |
| Rider et al., 2014 | 10 Division III cross-country runners, 3F (18.7 ± 0.6 y) and 7M (21.0 ± 1.3 y) | To determine whether wearing below-the-knee graduated compression stockings with a minimum of 15 mmHg of pressure during a maximal treadmill run would induce performance changes among collegiate cross-country runners. | TTE | 5-km running TT | During | Ankle: 20 mmHg  Calf: 15 mmHg | TTE was longer without compression sleeves |
| Rugg and Sternlicht 2013 | 14 healthy participants, 6F and 8M, (28.2 ± 14.0 y) | The purpose of this study was to investigate if wearing graduated compression tights, compared with loose fitting running shorts, help improve and sustain CMJ height after submaximal endurance running. | CMJ height | 15 min of continuous running with 5 min performed at 50%, 70%, and 85% of heart rate reserve | During | Ankle: 18.0 mmHg  Calf: 12.6 mmHg  Thigh: 7.2 mmHg | Compression tights maintained and increased power output after submaximal endurance running when compared with loose fitting running shorts. |
| Šambaher et al., 2016 | 15 active participants, 8F (22.3 ± 1.5 y) and 7M (24.8 ± 4.2 y) | To examine the effects of compression garments on neuromuscular performance, blood lactate, and skin temperature before and after fatigue | Pre and post a fatiguing protocol: plantar flexor MVC | Drop jumps from 30 cm | During | Ankle: 20 to 30 mmHg | There were no significant ankle compression-related changes in MVC plantar flexor |
| Scanlan et al., 2008 | 12 well-trained cyclists, M, (20.5 ± 3.6 y) | To investigate the effects of wearing lower body compression garments on performance responses during endurance cycling. | Absolute and relative mean and peak power, cadence, total work | One-Hour TT and incremental exercise test | During | Posterior gluteus maximus: 9.1 ± 2.2mmHg  vastus lateralis: 14.9 ± 2.3mmHg calf: 17.3 ± 3.0 mmHg  ankle: 19.5 ± 3.4mmHg | Compression improved relative power output at anaerobic threshold (4.2%) and absolute power output at anaerobic threshold (5.7%) |
| Sear et al., 2010 | 8 amateur team-sport athletes, M, (20.6 + 1.2 y) | To determine the effects of wearing whole body compression garments on physical measures during a team sport–specific prolonged high intensity intermittent exercise protocol. | Distance covered and running speed | 45 min prolonged high intensity intermittent exercise | During | 5 to 17 mmHg | There was a likely benefit of wearing whole body compression garments on total distance covered, distance covered during low intensity activity, and the variable self-selected high-intensity running speed |
| Shimokochi et al., 2017 | 17 college students, M, (21.8 ± 1.8 y) | To investigate whether recovery from muscle fatigue, occurring after repeated high-intensity muscle activities with eccentric contractions, is accelerated by wearing a compression garment during sleep via the changes in muscle force output and muscle electrophysiological index | Isometric MVC of the knee performed immediately and 24 h post-exercise | 10 x 10 maximal isokinetic eccentric and concentric knee extensor contractions, with 30-s rest intervals between the sets. Angular velocity was set 90°/s and 60°/s for knee extensor concentric and eccentric muscle contractions, respectively | Post exercise during sleep | NS | ~10% higher degree of recovery on average was observed in MVC 24 h after the fatiguing protocol under the compression garment condition |
| Smale et al., 2017 | 15 well-trained cyclists, M, (28.1 ± 6.3 y) | Examine the effects of varying grades of compression garments during incremental cycling exercise on cycling performance in well-trained cyclists. | TT performance | 4 x 8 min increments of cycling at 30%, 50%, 70%, and 85% peak power and a 4 km TT | During | Medium-grade garment at the ankle: 21.8 ± 6.6, knee: 20.3 ± 6.6, and thigh: 15.4 ± 4.5  Low grade compression: ankle: 8.6 ± 2.7, knee: 14.9 ± 4.9, and thigh: 9.1 ± 3.1 | Compression garments did not produce a distinct advantage for physical performance |
| Sperlich et al., 2010 | 15 healthy runners and triathletes, M, (27.1 ± 4.8 y) | Three types of compression clothing (socks, tights, and whole-body compression) on well-trained athletes to assess effects on performance. | TTE | 15 min sub-maximal running at 70% of VO_2max_ speed. Thereafter, running speed was set at the highest speed achieved during incremental testing. | During | 20 mmHg | No differences between compression clothing and clothing without external pressure |
| Sperlich et al., 2013 | 12 elite alpine skiers, M, (26.0 ± 4.0 y) | To evaluate the effects of different levels of compression on the legs of highly trained alpine skiers subjected to passive vibration in the downhill tuck position. | MVC and RFD during extension/flexion of knee muscles. CMJ height | 3-min trials in a downhill tuck position involving application of passive vibration to the soles of both feet | During and five minutes after | Moderate compression  calf: 19.7 ± 3.7 mmHg,  thigh: 17.8 ± 1.9 mmHg;  High compression  calf: 39.5 ± 3.5 mmHg,  thigh: 34.0 ± 2.6 mmHg | Compression on the legs of elite alpine skiers performing simulated skiing for 3 min in the tucked position with passive vibration resulted in a deeper tuck without compromising jumping performance |
| Sperlich et al., 2014 | 10 well-trained endurance athletes, M, (25.0 ± 4.0 y) | To assess whether upper body compression garments improved double-polling sprint performance (3 x 3-min simulated sprints on a cross-country ski ergometer) by enhancing power output | Change in power output | 3 x 3-min simulated double polling sprints on a cross-country ski ergometer | During | Forearm: 21 ± 5mmHg, Triceps brachii:14 ± 3 mmHg, Bicep Brachii: 14 ± 2 mmHg, Latissimus dorsi: 9 ± 2 mmHg | No effect on performance |
| Struhár et al., 2018 | 10 well-trained runners, M, (24.8 ± 3.45 y) | Identify the effect of compression garment pressure distribution on physiological and perceptual measures of performance and recovery | TT performance and ankle strength | 8 km running on a treadmill with a 6% elevation rate at the intensity of 75% of personal VO_2max_ | During and 4 h post run | Low grade compression: ankle: 18 mmHg, knee: 15 mmHg  Medium grade compression: ankle: 25 mmHg, knee: 21 mmHg  High reverse grade compression: ankle: 18 mmHg, knee: 24 mmHg | A beneficial trend in the promotion of ankle muscle performance with the medium-grade graduated compression |
| Terbizan et al., 2018 | 30 participants, M,  Control (21.56 ± 2.55 y),  Knee high stockings (21.80 ± 2.53 y), Waist high tights (20.91 ± 1.92 y) | To compare the effects of waist-high compression garments and knee-high compression stockings for recovery from plyometric box drops. | Isokinetic strength of the knee extensors and ankle plantar flexors, and vertical jump height. at 24, 48, and 72 h post-exercise | 10 x 10 plyometric box drop jumps from 60 cm box. Up to 10 s were allowed between drops, and 1 min between sets. | Worn for 12 h post-exercise | NS | Compression garments did not minimise indices or have an effect on recovery |
| Toolis and McGawley et al., 2020 | 7 senior biathletes from the Swedish national team, 3F and 4M, (25.1 ± 3.1 y) | To assess the effects of wearing upper- and lower-body compression garments on laboratory-based roller-skiing performance in elite biathletes, using ski durations and techniques simulating the demands of biathlon racing. | TT (15 min F, 20 min M)  TTE (20 km/h F, 22 km/h M) | Roller-ski time trial simulating the skiing duration of a biathlon sprint race, followed by a time-to-exhaustion test designed to elicit exhaustion within ∼60 to 90 sec | During | Biceps: 7.4 ± 2.2 mmHg, Triceps: 7.9 ± 2.2 mmHg, Brachioradialis: 13.1 ± 4.5 mmHg, Rectus femoris: 13.3 ± 2.3 mmHg, Gastrocnemius: 19.9 ± 5.9 mmHg | No statistically significant effect on either TT or TTE performances. However, compression was shown to elicit a small beneficial effect on TT and TTE test performances |
| Treseler et al., 2016 | 19 physically active participants, F, (20 ± 1 y) | To examine the performance effects of wearing below-the-knee compression stockings after a 5-km running test in recreationally active women | TT performance | 5km TT | During | Ankle: 18 to 21 mmHg  Kne: 12.6 to 14.7 mmHg | No effect on 5-km performance time |
| Tsuruike and Ellenbecker. 2013 | 24 college athletes, M, 12 tennis players (19.8 ± 0.9 y) and 12 soccer players (19.9 ± 0.3 y) | To examine the isotonic contraction of external rotation of the glenohumeral joint in 2 different ranges of force output and determine the effect of both long sleeve compression garments and ongoing visual feedback information on muscular performance | MVC | 5 consecutive repetitions of both concentric and eccentric contractions at 20–30% of MVC followed by 40–50% of MVC | During | NS | At 40–50% of MVC, the subjects maintained consistent force outputs in consecutively repeated concentric and eccentric contractions for external rotation of the glenohumeral joint when they wore compression garments of the presence of feedback. In contrast, the subjects showed fluctuations in force outputs without compression garments. |
| Upton et al., 2017 | 19 club level rugby union players, M, (20.3 ± 1.7 y) | To evaluate the efficacy of compression garments for the recovery of strength, power and indices of muscle damage from a rugby specific, muscle damaging protocol. | Isometric MVC and CMJ height at baseline, post, 24 and 48 h post-exercise | 20 x 20-m maximal sprints with 10 m deceleration. | 48 hours post-exercise | Calf: 14 ± 4.1 mmHg Thigh: 8.5 ± 2.3 mmHg | There was no significant group effect for MVC or CMJ. |
| Varela-Sanz et al., 2011 | 16 well-trained runners, 3F (32.0 ± 4.58 y) and 13M (35.4 ± 6.61 y) | To assess the influence of beneath-knee gradual elastic compression stockings on performance at competitive velocities in a group of well-trained runners | TTE | Running to exhaustion at 1% incline and 105% of recent 10-km time (17 ± 2 km/h) | During | Ankle: 15 to 22 mmHg | Wearing gradual elastic compression stockings showed a tendency to improve endurance TTE |
| Venckunas et al., 2014 | 13 active healthy adults, F, (25.1 ± 4.2 y) | To evaluate the effect of lower body compression garments on running performance in a thermoneutral environment. | Time of max effort running laps. | 4 km was covered in 30 min + 400m sprint | During | Thigh: ~17mmHg, upper calf: ~19mmHg | No effect on running performance |
| Vercruyssen et al., 2017 | 12 competitive trail runners, sex NS (39.6 ± 4.6 y) | The influence of wearing compression garment vs. conventional running clothing on muscle contractile function and RE before and after trail running. | Trail performance | 18.4-km short distance trail runs | During | Ankle: 18 mmHg  Calf: 13 mmHg | There were no benefits from wearing compression garments on trail running performance. |
| Vercruyssen et al., 2014 | 11 trained runners, M, (34.7 ± 9.8 y) | To investigate the effects of wearing compression socks on performance indicators during prolonged trail running | CMJ, MVC, run time. | 15.6 km trail run | During | Calf: 18 mmHg | The findings suggest that competitive runners do not gain any performance benefits from wearing compression during prolonged off-road running |
| Wang et al., 2016 | 12 track and field athletes, M, (21.2 ± 1.4 y) | Explore the influence of compression on the muscle force and endurance of the quadriceps femoris by using a dynamometer | Muscle force and endurance measured by peak moment normalized by mass, peak power normalized, average power across the first five degrees, total work, work fatigue. | 25 consecutive maximal concentric muscle actions of the quadriceps at randomly ordered angular velocities of 60 and 300◦/s | During | NS | No effect on quadriceps performance |
| Wannop et al., 2015 | 10 competitive recreational athletes, M, (age NS) | To determine how systematically increasing upper leg compression and hip joint stiffness independently from one another affects vertical jumping performance | Jump height and time | Vertical jumping | During | NS | Compression improved vertical jumping performance |
| Willems and Webb, 2010 | 18 participants, M, (20 ± 1 y) | Examine the effect of wearing lower body compression garments during downhill running on recovery of jump height | Jump height | 5 x 8 min bouts of downhill running at 80% of VO_2max_ and a -10% gradient. 2-min static recoveries after each bout | During exercise | Calf: 18mmHg Thigh: 9mmHg | Wearing a graduated compression garment during eccentric exercise did not enhance functional recovery |
| Williams et al., 2020 | 10 trained university-level cyclists, M, (21.0 ± 2 y) | To assess the effects of varying levels of compression applied via lower-limb compression garments on multiday cycling performance at typical levels of EIMD associated with multiday exercise events | TT performance | High intensity protocol, 24h rest, then a 8km time trial | During | Low-pressure compression garment:  distal hem: 7 ± 3 mmHg,  calf: 7 ± 3 mmHg,  mid-thigh: 5 ± 2 mmHg,  head of femur: 5 ± 2 mmHg,  posterior superior iliac spine: 5 ± 1 mmHg  High-pressure compression garment:  distal hem: 11 ± 3 mmHg,  calf: 15 ± 3 mmHg,  mid-thigh: 10 ± 3 mmHg,  head of femur: 8 ± 2 mmHg,  posterior superior iliac spine: 6 ± 1 mmHg | 8-km TT performance was significantly improved with higher compression levels compared with a lower-level compression garments and loose-fitting clothing controls by ~6% |
| Zadow et al., 2020 | 46 marathon runners, Compression group, 4F and 19M (45.8 ± 10.0 y); Control group, 8F and 15M (41.9 ± 9.9 y) | To determine if wearing lower-body compression socks would reduce intestinal damage associated with running a marathon | Run time | Marathon | During | Ankle: 25 mm Hg | No effect on run time |
| Zhang et al., 2016 | 12 healthy track and field athletes, M, (21.2 ± 1.4 y) | Determine effectiveness of thigh compression apparel on 1) the force and endurance of the quadriceps and 2) the EMG amplitude and mean power frequency of the *rectus femoris, vastus lateralis,* and *vastus medialis* during repeated concentric muscle actions of dominant leg. | Isokinetic strength: 25 consecutive maximal concentric contractions of the quadriceps and hamstrings at randomly assigned 60 and 300˚/s. Peak momentum, total work, work fatigue | 25 maximal repeated isokinetic knee extensions at 60 and 300◦/s on a dynamometer | During | NS | Compression of the lower extremity did not significantly enhance strength production in a short period. |
| Zinner et al., 2017 | 12 handball players, M, (22 ± 4 y) | To investigate the effects of increasing the level of compression on recovery following repeated sprints. | 5 x 30m sprint performance (pre- and 48 h post) and jump height (pre- and 48 h post) | 30 x 30m sprints | 48 hours post exercise | From below the hip to the foot exerting mean pressures applied of 3 ± 1 mm Hg, 11 ± 1 mm Hg, 23 ± 2 mm Hg | Jump performance was ‘likely’ improved when wearing 0 mmHg of compression during recovery, compared to 10 mmHg and 25 mmHg. With regard to sprint performance no differences were evident between all levels of compression |

M = Male, F = Female, CMJ = Countermovement jump, NS = Not specified, TT = Time trial, GPS = Global positioning system, AFL = Australian Football League, WAnT = Wingate Anaerobic Test, YoYoIE2 = YoYo intermittent endurance test level 2, TTE = Time to exhaustion/fatigue, VO_2max_ = Maximal oxygen uptake, RFD = Rate of force development, MVC = Maximal voluntary contraction, EMG = Electromyography, DOMS = Delayed-onset of muscle soreness, ROM = Range of motion, IMTP = Isometric mid-thigh pull, 1RM = 1-Repetition maximum, EIMD = exercise-induced muscle damage.
