# Supplementary material for: Putting the Squeeze on Compression Garments: Current Evidence and Recommendations for Future Research: A Systematic Scoping Review
Source: Sports Med. 2021 Dec 6;52(5):1141–60. doi: 10.1007/s40279-021-01604-9 (PMC9023423; doi:10.1007/s40279-021-01604-9)
Supplement: Supplementary file 2 — Supplementary file2 (DOCX 65 kb) [file 40279_2021_1604_MOESM2_ESM.docx]

**Supplementary Table S2.** Details of studies and information relevant to biomechanical and neuromuscular outcomes.

| **Study** | **Cohort/ sample size (n), sex, age** | **Study purpose** | **Outcome Measures** | **Exercise Protocol** | **Compression worn during/after/both** | **Compression pressure – reported value or not stated** | **Key findings** |
| --- | --- | --- | --- | --- | --- | --- | --- |
| Angelakos et al., 2020 | 16 participants, 7F and 9M, (24.3 ± 2.9 y) | To examine the effects of compression garments of varying lengths (i.e., full leg, below the knee including the ankle and foot, and only calves) and tightness levels (wearing two of the same type or one) on stability and lower body joint alignment during a forward lunge | Lateral pelvic tilt, maximal knee valgus, maximal inversion/eversion, and mean minimum time to boundary of the centre of pressure | Three bodyweight, forward lunges | During | NS | Mean minimum time to boundary of the centre of pressure was significantly greater under ‘Heavy’ compression compared to ‘Light’ compression and control conditions.  No effects of compression level or length on lower limb kinematic variables during lunging |
| Areces et al., 2015 | 34 marathon runners, 4F and 30M,  Control group: (42.7 ± 7.8 y), Compression group: (41.2 ± 8.9 y) | Investigate the benefits obtained by wearing graduated compression stockings on running pace, prevention of muscle damage and maintenance of muscle performance during a real marathon race. | Drop of centre of gravity during the landing phase | Marathon race | During | 20 to 25 mmHg | No effect on centre of gravity |
| Barrs et al., 2018 | Experiment 1: 13 participants, 8F and 5M, (22.9 ± 3.0 y)  Experiment 2: 12 participants, 7F and 5M, (25.2 ± 2.3 y) | To examine whether compression apparel produces measurable modulation of sensory feedback and motor task performance in the arm | Experiment 1: H-reflex recruitment curves, 10% MVIC of the *flexor carpi radialis*, contraction + superficial radial nerve conditioning, contraction + distal median nerve conditioning. EMG of the *extensor carpi radialis, biceps* and *triceps brachii.*  Experiment 2: same as experiment 1 but during an ergometer test. | Experiment 1: electrical stimulation  Experiment 2: arm cycling at 60 revolutions per min | During | 10 to 20 mmHg | A customized compression sleeve worn around the elbow joint alters reflex excitability and improves reaching accuracy. This alteration in excitability occurs in cutaneous and muscle afferent sensory pathways and across multiple movement tasks |
| Bernhardt and Anderson, 2005 | 13 participants, 3F and 10M, (25.7 y) | Examine issues of performance and proprioception at the hip with the use of elasticized compression shorts that offer considerably more compression and resistance to movement. | Active ROM (flexion/hyperextension/abduction), joint angle replication (flexion /hyperextension/abduction) and stork balance test | Tests of ROM, jumping, balance, sprinting, agility, and a shuttle test. | During | NS | Active ROM during hip flexion was reduced with compression |
| Book et al., 2016 | 14 participants, 5F and 7M, (27.3 ± 6.4 y) | To determine how applied external compression to the lower leg influences local and/or central hemodynamic responses as an indicator of the potential for improved athletic performance when using graduated compression stockings. | Muscle activity via EMG (*gastrocnemius*) | 5-min of plantar flexion  exercise, followed by standing in a natural posture for a 5-min recovery period | During and 5 min after | Pressure difference from ankle to knee of ~10 mmHg | No effect on muscle activity |
| Born et al., 2014 | 12 track and team sport athletes, F, (25.0 ± 3.0 y); Sub-study 2: 12 track and team sport athletes, F,  (23 ± 2 y) | There were 2 aims; 1) To assess the effects of compression garments with silicone stripes (which mimic kinesio taping) on repeated sprint performance; 2) to identify the physiological, biomechanical, and perceptual effects of compression garments with silicone stripes | Hip flexion angle, step length, step frequency, and EMG of 5 muscles (*gluteus maximus, rectus femoris, vastus lateralis, biceps femoris, and gastrocnemius)* | 30 x 30-m sprints (one sprint per minute) | During | ~18 to 20 mmHg across the entire lower body | Compression garment significantly reduced hip flexion angle. During the final 10 sprints, compression with silicone strips increased step length, without altering step frequency, and enhanced EMG activity in the *rectus femoris* muscle only |
| Broatch et al., 2021 | 42 participants, 21F and 21M, (27 ± 4 y) | To assess the effects of commercially available sports compression socks on somatosensory ability at the ankle joint, specifically inversion/eversion movement. | Somatosensory discrimination during ankle inversion/eversion movements | 4 sets of 50 ankle inversion/eversion movements to varying degrees (between 10.5 and 14.5˚ from the horizontal) | During | Calf: ~23 mmHg  Ankle: ~20 mmHg | Wearing compression may amplify sensory input in a way that enhances somatosensation for individuals with poor somatosensation, but overloads input and impairs somatosensation of those with good somatosensation. Compression also no effect on somatosensation when participants were split based on ankle instability |
| Broatch et al., 2020 | 27 recreational-active, M  Study 1, n=13 (22 ± 3.0 y),  Study 2, n=14 (27 ± 5.0 y) | To investigate the effectiveness of three different commercially-available lower-limb sports compression garments in reducing muscle displacement, soft-tissue vibrations, and muscle activation during running at different speeds | Study 1: muscle displacement and acceleration  Study 2: muscle displacement, muscle vibrations (calculated from acceleration), EMG of *Vastii* and *Gastrocnemii* | Study 1: 4-min treadmill running bouts (2 min at 12 km/h and 15 km/h)  Study 2: 9-min treadmill running bouts (3 min at 8 km/h, 10 km/h, and 12 km/h) | During | 6 pressure sites all measured in mmHg: a) 5 cm proximal to medial malleolus, b) 5 cm proximal to a), c) maximal calf girth, d) thigh 10 cm below land- mark, e) midthigh, f) 5 cm proximal to landmark e.  2XU Tights: a) 13.2 ± 2.9, b) 17.2 ± 6.2, c) 21.8 ± 6.0, d) 12.0 ± 2.2, e) 12.1 ± 2.3, f) 10.7 ± 2.9  Nike Pro Zonal Tights: a) 9.1 ± 2.5, b) 14.6 ± 4.9, c) 21.5 ± 5.1, d) 11.3 ± 2.1, e) 12.9 ± 2.7, f) 12.7 ± 2.1  Under Armor Charged Tights: a) 7.7 ± 3.1, b) 11.4 ± 4.7, c) 18.9 ± 6.3, d) 13.3 ± 3.1, e) 13.2 ± 3.1, f) 12.6 ± 2.9 | Compression tights worn during submaximal treadmill running reduced markers of muscle displacement and soft-tissue vibrations in the lower limb. Compression-induced reductions in muscle displacement also corresponded to a reduction in muscle activation |
| Carling et al., 1995 | 23 participants,  16F and 7M, (26 ± 4 y) | Evaluate the effect of compression on DOMS and the accompanying manifestations of soreness, swelling, range of motion, and isokinetic strength for a period of 4 days following induction of DOMS | Elbow extensor ROM | 70 eccentric contractions of the elbow flexor muscles, dynamometer test (120˚/s through 120˚ of motion) | After | Sleeve pressure: 17 mmHg | No significant differences were present for either group |
| Cavanaugh et al., 2015 | 12 participants, 5F (23.7 ± 4.3 y) and 7M (24.1 ± 5.7 y). | To investigate the effect of a knee compression sleeve and kinesio tape on balance before and after fatigue | Y-Balance test: Distance in cm reached by the non-dominant foot; Drop Jump Landing: Vertical GRF and the excursion of centre of pressure; EMG: *vastus lateralis, vastus medialis, and biceps femoris* | 4 sets of unilateral Bulgarian squats with body weight to failure with 1 min rest intervals | During | NS | No effect of compression on balance outcomes after the fatiguing intervention. No effect of compression on EMG signals from drop jump landing |
| Chaudhari et al., 2014 | 29 participants, 13F and 16M, (23.4 ± 4.8 y) | To test whether the directional compression reduces hip adductor activation | EMG of *adductor longus* during a 45˚ unanticipated side-step run-to-cut manoeuvres | 45° run-to-cut manoeuvres | During | NS | The directional compression shorts altered adductor activation during run-to-cut manoeuvres and demonstrated reduced adductor activation in the directional condition |
| Cheng and Xiong 2019 | 16 participants, M, (22.5 ± 0.9 y) | To explore the effects of compression stockings on metabolic cost, muscle activation, kinematics and joint kinetics during walking | EMG of the *tibialis anterior, gastrocnemius lateralis,* and *soleus,* joint angles, joint moments, and joint powers of the hip, knee and ankle, step frequency, step length, step frequency variability, and step length variability | Participants walked at a speed of 5 km/h for 6 min.  Participants walked along a 10 metre level walkway following a metronome. | During | 30 to 40 mmHg | Compression stockings have trivial effects on the kinematics and muscle activation for most participants. The joint kinetics of the ankle may become adjusted as a consequence of the pressure exerted by compression stockings |
| Choi and Hong 2019 | 7 participants, M, (21.4 ± 1.7 y) | To determine the differences in the EMG of the thigh among film-welded compression suits, film-free compression suit and a loose sportswear during isokinetic exercise of the knee. | Mean muscle activity and maximum muscle activity at 180°/s, and 240°/s from the *rectus femoris, vastus lateralis, vastus medialis oblique, semitendinosus,* and *bicep femoris.* | A set of five trials at an angular velocity of 60°/s, 10 trials at 180°/s, and 30 trials at 240°/s | During | Compression suit pressure: 0.7 to 3.5 kPa  Film-welded suit on the compression suit: 1 to 5.3kPa | Wearing film-welded compression suits enhanced mean muscle activity of *vastus lateralis* and *vastus medialis oblique* at each angular velocity, however, it did not support the mean muscle activity of the hamstrings in almost all conditions of exercise |
| de Britto et al., 2017 | 27 participants, F, (23 ± 4 y) | To investigate the influence of a compressive garment on knee kinematics during jump-landing tasks performed by physically active women | Knee valgus angle at initial ground contact (left columns) and peak knee valgus angle (right columns) during the landing phase in the five jump-landing, knee flexion angle at initial ground contact, and peak knee flexion angle | Forward jump, forward jump with countermovement, 20 cm drop jump with countermovement, 40 cm drop jump with countermovement, and vertical jump | During | Size 44: 11.3 mmHg  Size 46: 10.5 mmHg  Size 48: 8.3 mmHg | Compressive shorts lead to smaller knee flexion and valgus angles in jump-landing tasks |
| Del Coso et al., 2013 | 36 experienced triathletes, Sex NS, Control group, n=17, (35.8±6.3y),  Compression group, n=19, (35.0±5.3y) | To investigate the potential of compression stockings to prevent muscular damage and to preserve muscular performance during a half-ironman competition. | Second peak of GRF | Half-iron man | During | NS | No effect on GRF |
| Doan et al., 2003 | 20 track athletes,  10F (19.2 ± 1.3 y) and 10M (20.0 ± 0.9 y) | To determine how custom-fit compression shorts affect athletic performance and to examine the mechanical properties of the shorts. Specific performance and mechanical tests were designed to assess the effect of the garment on muscle oscillation, jump power, skin temperature, impact absorption and elasticity. | Hip and knee ROM, muscle oscillation and GRF during counter-movement jump | 60-m sprint; countermovement jump; 60 s intervals cycling | During | NS | The compression garment was related to decreased muscle oscillation. The elasticity of the garment provides increased flexion and extension torque at the end range of extension and flexion, respectively. Lastly, hip joint ROM decreased slightly during a 60 m sprint |
| Duffield et al., 2010 | 11 team sport athletes, sex NS, (20.9 ± 2.7 y) | Examine the effect of wearing compression garments during and 24 h following high-intensity, intermittent-sprint and stretch shortening cycle activities on post-exercise performance and recovery of evoked and voluntary muscle performance. | Peak twitch force data elicited from electrical stimulation. | 10-min exercise protocol of a 20-m sprint and 10 plyometric bounds every min | During and for 24 h after | NS | Compression garments had no effect on evoked or voluntary muscle performance during and/or up to 24 h following intermittent-sprint and stretch shortening cycle activity |
| Ehrstrom et al., 2018 | 13 trail runners, M, (38.6 ± 5.7 y) | To examine whether wearing high-pressure compression garments during a 40-min treadmill downhill run on acute and delayed neuromuscular responses and running economy | Stride frequency, voluntary activation, M_wave_ peak-to-peak amplitude, potentiated twitch torque, low-frequency doublet force, high-frequency doublet force, and low-to-high doublet frequency ratio | 40-min downhill running at –8.5˚ decline | During | 15 to 20 mmHg for quadriceps and calves | The use of high-pressure compression garments during downhill running induces beneficial effects on soft-tissue vibrations, as well as acute and delayed neuromuscular responses in well-trained off-road runners. |
| French et al., 2008 | 26 participants, M, (24.1 ± 3.2 y) | To evaluate contrast bathing and contrast garments as regeneration strategies after exercise-induced muscle damage | ROM for hip flexion/extension/abduction, knee flexion/extension, and ankle dorsiflexion | 6 x 10 squats with 100% of body mass + 5 second eccentric repetition with the participants one-repetition maximum | For 12 hours after exercise | Calf: 12 mmHg  Thigh: 10 mmHg | No effect on ROM |
| Fu et al., 2012 | 12 participants, M, (21.2 ± 1.4 y) | To examine the effects of compression levels on muscle strength, EMG and MMG of the *rectus femoris* during isometric and isokinetic muscle actions at both low (60˚/s) and high (300˚/s) angular velocity | EMG + MMG from *rectus femoris* (dominant leg) | Two sets of 5 secs of MVIC of the quadriceps with the knee joint angle fixed at 30˚ of extension and hip joint angle set at ~ 90˚ of flexion. Isokinetic muscle actions at both low (60˚/s) and high (300˚/s) angular velocity | During | Medium: 66.4 N  High: 85.8 N | The high level of compression exerted on the *rectus femoris* lowered muscle activation and induced a higher EMG mean power frequency at 60˚/s, whereas no significant compression effect was found in the MMG responses. |
| Ghai et al.2018 | 44 participants, 18F and 26M, (22.7 ± 6.9 y) | Investigate the effects of below-knee compression garments on knee joint proprioception under conditions of high and low conscious attention | Repositioning of the dominant and non-dominant leg | Return leg to a previously identified target position (30 or 60˚) | During | 10 to 15 mmHg | Below-knee compression garments may improve proprioception of the knee, regardless of leg dominance. Secondary tasks that direct attention away from proprioceptive judgments may also improve proprioception, regardless of the presence of compression |
| Gupta et al., 2015 | 38 participants, M, (22.1 ± 2.8 y) | To determine the effect of compression garments on spatiotemporal and leg mechanical characteristics during single-leg hopping to volitional exhaustion | Flight phase, loading phase, contact phase, vertical displacement of the centre of mass during flight phase, vertical displacement of the centre of mass during loading phase, normalised peak vertical GRF, and normalised vertical leg stiffness | Single-leg hopping to volitional exhaustion | During | NS | Compression garments did not have any significant effect on spatiotemporal characteristics or vertical stiffness during single-leg hopping to exhaustion. |
| Hasan et al., 2016 | 12 participants, M, 6 trained (15.7 ± 0.7 y), 6 untrained (15.2 ± 1.1 y) | To investigate the effects of wearing textured insoles and clinical compression socks on kicking performance | Initial ball velocity, maximum foot velocity, foot velocity at ball contact, ankle ROM, hip ROM, knee ROM, and planting foot placement | 20 instep kicks with maximum effort | During | 20 to 30 mmHg | Wearing textured and compression materials enhanced performance in key variables, such as the maximum velocity of the instep kick and increased initial ball velocity, among advanced learners compared to the use of non-textured and compression materials. |
| Heiss et al., 2018 | 15 participants, 7F and 8M, (25 ± 6 y) | To investigate the influence of commercially available sport compression garments on the development of exercise-induced intramuscular oedema | ROM of the ankle. | All participants performed 5 sets of 30 repetitions of calf raises and rested 10 sec between each set with 25% of their body weight during the exercise. | The compression sock was worn continuously for 60 h after eccentric exercise and was removed for the first time for follow-up examination | NS | No effect on ROM |
| Hintzy et al., 2019 | 12 participants, M, (25.3 ± 3.6 y) | Examine the effects of different levels of thigh compression in shorts on both vibration and muscle activity of the thigh during cycling with superimposed vibrations. | Acceleration and vibration of the longitudinal axis of the right *vastus lateralis* muscle; EMG from the right *vastus lateralis* | Four sets of 18-minute cycling test | During | Up to 15 mmHg | Compressing the muscle belly with compression shorts (pressure at least 6 mmHg) allows the reduction of muscle vibration on the thigh during cycling with superimposed vibrations. As a consequence, the *vastus lateralis* presents lower activity when wearing compression shorts. |
| Kerhervé et al., 2017 | 14 participants, M, (21.7 ± 3.0 y) | To determine if wearing calf compression sleeves during a prolonged running exercise affected local muscle tissue oxygenation, running pattern, muscle power capability, performance, and subjective perception of muscle fatigue, pain and soreness. | Biomechanical running pattern (i.e., contact time, aerial time, stride frequency, duty factor, lower limb, and vertical stiffness) | 24 km run | During | 23 ± 2 mmHg | Wearing compression sleeves compared to control during trail running modified running pattern and muscle mechanical capabilities during hopping via increased leg stiffness |
| Kraemer et al., 1998 | Proprioception Study: 12 participants, 6F and 6M (21.3 ± 2.9 y)  Oscillation Study: 10 participants, 5F and 5M (22.0 ± 2.5) | To investigate the influence of a compression garment on joint position sense at the hip and muscle movement velocity upon landing impact. | Hip joint position sense  Quadriceps muscle movement | Proprioception Study: Hip angle replication at 30, 45, 60, and 90˚  Oscillation Study: 3 x 6 maximal jumps | During | NS | The compressive garment enhanced joint position sense at the hip at 45° and 60° of flexion. A compression garment also significantly reduced the vertical velocity of muscle movement upon landing |
| Kraemer et al., 2001 | 15 non-strength-trained participants, M, Compression group (22.3 ± 2.9 y), Control group (22.1 ± 3.3 y) | To determine whether a compression sleeve worn immediately after maximal eccentric exercise enhances recovery | Resting-elbow angle | 2 sets of 50 repetitions at 60°/s on an isokinetic dynamometer with 3 min rest between sets. Every fourth repetition, the subject performed a MVIC at end range, followed by an eccentric contraction in which the subject resisted mechanically forced elbow extension | After | NS | Compression sleeves prevented loss of elbow extension |
| Kraemer et al., 2001 | 20 non-strength-trained participants, F,  Compression sleeve group (21.3 ± 2.9 y),  Control group,  (21.1 ± 3.3 y) | To investigate whether constant compression via the use of a compressive arm sleeve would reduce the severity and duration of soreness associated with DOMS. | Elbow ROM | Dynamometer (60°/s). 2 sets of 50 repetitions with 3 minutes rest between sets. Every fourth repetition, the subject performed a maximal concentric contraction with an isometric hold followed by an eccentric contraction | After | 10 mmHg | Compression was found to prevent the loss of elbow extension at rest in the exercised arm |
| Kraemer et al., 2016 | 19 recreationally-active participants, M, (23.1 ± 2.4 y) | To examine the impact of trans-American jet travel on physical performance and associated hormonal and sleep-related responses to gain insight into potential mechanistic contributions to any reductions in physical performance. Secondarily, to assess the impact of a return flight on recovery processes following a demanding physical activity and whether a compression garment intervention could ameliorate any of the tissue damage upon return to the original time zone. | Quickboard (measure of quickness and reaction) | Roundtrip trans-American jet travel | During | NS | The compression group demonstrated no significant physical performance differences from baseline testing and also showed significantly better performances than the control group at the corresponding time points |
| Kurz and Anders 2018 | 22 recreationally-active participants, M, (25.3 ± 2.8 y) | To identify the effect of lower leg compression sleeves on muscle activation cost during submaximal non-fatiguing locomotion | EMG of five lower leg muscles (*tibialis anterior, fibularis longus, lateral and medial head of gastrocnemius, and soleus*).  Cumulative muscle activity per distance travelled | Each participant ran on a treadmill at four different speeds (ordered sequence of 2.8, 3.3, 2.2 and 3.9 m/s). | During | Distal: 4.0 kPa  Proximal: 2.7 kPa | Application of lower leg compression during treadmill running for short distances reduced cumulative muscle activity per distance travelled levels. The findings suggest an optimized muscle activation cost of lower leg muscles while wearing compression sleeves during sub-maximal dynamic effort that has the ability to postpone muscle fatigue during endurance running. |
| Kuster et al., 1999 | 36 participants post ACL reconstruction, 12F and 24M, (31.7 ± 9.9) | To examine the possibility of increased muscle coordination after ACL reconstruction through the wearing of a compression sleeve. | 10-cm standing drop jump and peak forces during landing phase, adjusting phase, and balancing phase. | 10-cm standing drop jump from an elevated platform onto a force plate, to land on one leg, and thereafter maintain a one-legged balance for 25 s | During | NS | A compression sleeve improved the total integration of the balance control system and muscle coordination |
| Lee et al., 2016 | 7 participants, M, (20 to 29 y) | To analyse relationship between different design factors of compression garments and influence on single-legged drop landing motions. | Angular velocity, ROM, oscillation of the lower limbs, and GRF | Three trials of a 40-cm single-legged (dominant leg) drop landing | During | NS | Subtle manipulation of the level, location, or the method of pressurization significantly changes the stability of joints and the performance of exercise. |
| Lee et al., 2017 | 12 participants, sex NS, (24.0 ± 2.2 y) | To 1) assess the changes in basic motor skills and brain reaction that result from wearing compression pants; 2) to assess the effects of pressure levels on perception and recognition | Movement-related cortical potentials | Two side steps | During | Garment 1:  Knees: 0.44 to 0.58 kPa  Thighs: 0.48 to 0.68 kPa  Garment 2:  Knees: 0.95 to 1.03 kPa Thighs: 0.53 to 0.71 kPa  Garment 3:  Knees: 1.67 to 2.12 kPa  Thighs: 0.80 to 1.14 kPa | Wearing compression pants with the highest-pressure level increased the movement related cortical potentials |
| Lucas-Cuevas et al., 2015 | 40 recreational runners, 20F and 20M, (28.4 ± 5.9y) | To analyse the effect of 3 weeks of training with graduated compressive stockings and non-compressive stockings and muscle fatigue on stride kinematics, impact acceleration and perception of comfort during running | Impact acceleration parameters measured for 15 s every 5 min, stride length, and stride frequency | After 3 weeks of training testing occurred during 30 min running at 80 % of the individual’s maximal aerobic speed | During training and testing | Ankle: 24 mmHg  Calf: 21 mmHg | Longer use of compression garments led to a significant reduction in impact acceleration during running compared to placebo garments. Moreover, compressive stockings were able to reduce the rate of increase in the acceleration parameters as fatigue developed |
| Lucas-Cuevas et al., 2017 | 36 runners, 15F (29.17 ± 3.8 y) and 21M (28.14 ± 4.46 y) | To analyse the influence of graduated compression stockings on the perception of comfort and muscle activation of the lower leg during high intensity running | EMG of the *tibialis anterior*, *peroneus longus, gastrocnemius medialis, and gastrocnemius lateralis* | 20-min run at 75% of their maximal aerobic speed at 1% slope on a treadmill | During | Ankle: 24 mmHg  Calf: 21 mmHg | The use of graduated compression stockings reduced *gastrocnemius* muscle activity during the 5 min of the run, but their effect was temporary. |
| Mancini et al., 2020 | 14 healthy participants, 9F and 5M, (46.3 ± 7.7 y) | To investigate the effects of elastic stockings on H-reflex from soleus muscle under rest and after a walking program | M_wave_ Latency, M_wave_ amplitude, H-reflex latency, H-reflex amplitude, H-reflex threshold, Sural nerve amplitude, Sural nerve sensory conduction velocity | Walking 150 m | During | Ankle: 23 to 32 mmHg | No effects were reported on the peripheral nervous system |
| Maton et al., 2006 | 15 participants, Sex NS, (32 ± 6 y) | To test if elastic compressive stockings increase muscle fatigability during sustained muscle contraction or if it improves recovery after fatigue | Surface EMG recorded on 4 leg and thigh muscles. | Three sets of 50% ankle dorsal flexion until fatigue, separated with 30 s of rest | During | Ankle: 23.6 mmHg  Calf: 14.3 mmHg | No effect on EMG |
| Michael et al., 2014 | 12 participants, F, (24 ± 7.2 y) | To assess the effectiveness of wearing compression garments on the balance ability of elite athletes, and to investigate the effects of compression garment sizing on postural control | Centre of mass and pressure path lengths (sagittal plane, frontal plane and average sway) and overall stabilisation time | Single leg balance task for up to 60 s | During | NS | Well-fitted compression improved balance time and decreased postural sway variability compared with conventional shorts in the eyes closed condition.  No effect of loose-fitted compression.  No effect of compression garments on static balance and postural control with eyes open |
| Mills et al., 2015 | 8 participants, M, (24.1 ± 3.0 y) | To measure the effects of compression garments and Kinesio Tape on lower extremity joint kinematics during a lunge prior to and following an exercise bout. | Maximal lateral pelvic tilt, maximal knee valgus, and maximal ankle eversion/inversion during each lunge. | Three rotational lunges, 10 min running at 70% of maximal intensity, then three rotational lunges | During | NS | No effect of compression on joint kinematics prior to exercise. However, compression maintained joint alignment following exercise |
| Miyamoto et al., 2011 | 14 participants, M, (25.6 ± 3.7 y) | To examine the effects of elastic compression stockings on the torque generating capacity of the *triceps surae* muscle and EMG activity | EMG of the medial *gastrocnemius and soleus*, triplet torque, M-Wave, peak torque and associated mean power frequency and root mean square, and MVIC | 15 sets of 10 calf raises with 30 s recovery between sets | During | Ankle: 18 to 30 mmHg | No effect of compression on the decline of MVIC torque regardless of the pressure, whereas the extent of reduction of the evoked triplet torque was smaller when wearing compression stocking with a high compression pressure |
| Miyamoto and Kawakami 2015 | 15 participants, M, (25.2 ± 2.6 y) | To examine the effect of pressure profiles of compression stockings on muscle fatigue level of the lower leg muscles induced by running exercise, and to test the pressure profiles against the development of muscle fatigue. | Muscle transverse relaxation time (T2) immediately after exercise in the *medial* and *lateral gastrocnemius, soleus,* and *tibialis anterior* | Running on a treadmill set at 0- inclination for 34.5 min including 4.5-min warm-up, 1.5 min at 6 km, 1.5 min at 8 km, 1.5 min at 10 km, and 30 min at 12 km | During | Graduated low pressure compression condition, gastrocnemius: 14mmHg and ankle:18 mmHg  Graduated high pressure compression condition, gastrocnemius: 21 mmHg and ankle: 27 mmHg  Uniform pressure distribution condition, gastrocnemius = 21 mmHg; Ankle =21 mmHg  Localized pressure at the gastrocnemius region condition, gastrocnemius: 21 mmHg and ankle: 10 mmHg | No effect |
| Moreno‐Pérez et al., 2020 | 16 middle‐distance endurance athletes, 2F and 14M, (33.4 ± 6.3 y) | To evaluate changes in EMG activity with the use of gradual compression stockings on middle‐distance endurance athletes | EMG of the *vastus lateralis, vastus medialis, rectus femoris, biceps femoris, gastrocnemius,* and *soleus* | 10 min at 3% above second ventilatory threshold; 1 km at velocity of maximal oxygen uptake | During | 15 to 20 mmHg | No effect on EMG |
| Négyesi et al., 2020 | 24 right-side dominant healthy adults, 12F and 12M, (25.5 ± 4 y) | Examine whether below-knee compression garment reduces fatigue-induced strength loss and joint position sense errors in healthy adults. | Joint position sense and ROM | 100 maximal isokinetic eccentric contractions at 30°/sec with the right-dominant knee extensors | During | NS | No effect on joint position sense errors or ROM |
| Pearce et al., 2009 | 8 participants, M, (23-27 y) | To examine if sports compression garments assist in the performance of a visuomotor tracking task following a bout of eccentric exercise, resulting in DOMS, at intervals up to 14 days post-exercise | Visuomotor tracking performance. | 35 maximal isokinetic eccentric extensions at 90◦/s | During | NS | Wearing compression garments positively affected performance during a tracking task |
| Pereira et al., 2014 | 22 resistance trained participants, M, (24.6 ± 5.1 y) | To examine the effect of graduated compression sleeves worn during exercise on muscle recovery in young resistance trained men. | Muscle activation (surface EMG) | 4 sets of 10 maximal elbow flexion/extension at 120˚/sec. 1 min separated sets | During | NS | No significant differences between groups for isometric peak torque, muscle activation, or echo intensity across 96 h post exercise |
| Perrey et al., 2008 | 8 participants, M, (26 ± 4 y) | To assess the adaptation response to DOMS while wearing graduated compression stockings | Neuromuscular measures (M_wave_, peak twitch, and maximal voluntary torque) | Subjects exercised by walking on a motorized treadmill for 30-min at a constant velocity of 1 m.s-1 with a negative grade of -25%. and a load of 12% of body mass | Graduated compression stockings were worn 5 h per day at 2 h, 24 h, 48 h and 72 h | NS | No effect of compression on strength and functional declines |
| Ravier et al., 2018 | 18 handball players, M, (23.22 ± 4.97 y) | To investigate benefits of wearing full-leg length compression garments during conventional handball-specific circuit exercise on maximal and rapid muscle force characteristics immediately at the end and 24 hours post-exercise. | Ground contact time. | Handball-specific circuit exercise during three 12-min periods. Exercise was separated by four mins of rest. | During exercise | Ankle: 15 mmHg  Medial gastrocnemius: 27 mmHg  Vastus lateralis: 14 mmHg | No effect on ground contact time |
| Šambaher et al., 2016 | 15 active participants, 8F (22.3 ± 1.5 y) and 7M (24.8 y ± 4.32 y) | To examine the effects of compression garments on neuromuscular performance, blood lactate, and skin temperature before and after fatigue | Evoked muscle contractile properties through EMG; Jump kinetics and kinematics | Drop jumps from 30cm | During | Ankle: 20 to 30mmHg | Twitch half-relaxation times were positively affected by ankle compression. There were no significant ankle compression-related changes in peak twitch force, time to peak twitch, and calf muscle EMG at any time points. Ankle compression was able to reduce peak GRF from the highest drop jump (50 cm). However, there were no changes between conditions for jump height, take-off velocity, or contact time |
| Shimokochi et al., 2017 | 17 college students, M, (21.8 ± 1.8 y) | To investigate whether recovery from muscle fatigue, occurring after repeated high-intensity muscle activities with eccentric contractions, is accelerated by wearing a compression garment during sleep via the changes in muscle force output and muscle electrophysiological index | EMG of the knee | Subjects performed 10 sets of 10 repetitions of maximal isokinetic eccentric and concentric knee extensor contractions, with 30-s rest intervals between the sets. Angular velocity was set 90°/s and 60°/s for knee extensor concentric and eccentric muscle contractions, respectively | Post exercise during sleep | NS | ~10% higher degree of recovery was observed in contraction force 24 h after the fatiguing protocol under the compression garment condition |
| Song et al., 2015 | 11 right-handed golfers, M, (46.3 ± 16.0 y) | To investigate the effects of wearing a compression top on golfers’ trunk and club motions when executing a full swing | Angular rotational difference between the shoulders and the hip at the top of backswing measured in degrees, angular rotational difference between the shoulders and the hip during the down swing, and peak velocity | 10 full golf club swings | During | NS | Wearing a compression top underneath golf T-shirts slightly restricted golfers’ trunk motion early in the downswing. Wearing the compression top affected angular rotational difference between the shoulders and the hip during the downswing but not during the backswing |
| Sperlich et al., 2013 | 12 elite alpine skiers, M (26 ± 4 y) | To evaluate the effects of different levels of compression on the legs of highly trained alpine skiers subjected to passive vibration in the downhill tuck position. | Single leg eyes-closed balance test, knee joint angle, and acceleration. | 3-min trials in a downhill tuck position involving application of passive vibration to the soles of both feet | During and 5 min after | Moderate compression: Calf: 19.7 ± 3.7 mmHg  Thigh: 17.8 ± 1.9 mmHg; High compression:  Calf: 39.5 ± 3.5 mmHg Thigh: 34.0 ± 2.6 mmHg | Compression on the legs of elite alpine skiers performing simulated skiing for 3 min in the tucked position with passive vibration resulted in a deeper tuck position. These changes occurred without compromising maximal leg strength or balance |
| Stickford et al., 2015 | 16 trained runners, M, (22.4 ± 3.0 y) | Investigate the impact of wearing lower-leg compression sleeves on the running economy and mechanics of highly trained distance runners. | Contact and toe-off time points, foot and ground contact time, swing time, step frequency, step length, and gait variability, and mechanical parameters of the spring-mass model during running, including vertical displacement, max ground-reaction force, vertical stiffness, leg spring stiffness | Four minute stages at each of three constant submaximal speeds of 233, 268, and 300 m/min on a motorized treadmill | During | Calf: 15 to 20 mmHg | Lower-leg compression sleeves did not alter leg stiffness or running mechanics during submaximal running. Those who improved running economy while wearing compression had lower measures of gait variability, particularly at the slowest speed |
| Varela-Sanz et al., 2011 | 16 well trained runners, 3F (32.0 ± 4.6 y) and 13M (35.4 ± 6.6 y) | To assess the influence of beneath-knee gradual elastic compression stockings on running economy and performance at competitive velocities in a group of well-trained runners | Kinematic running technique data including contact time, flight time, height, power generated, frequency and stride length | Running to exhaustion at 1% incline and 105% of recent 10-km time (17 ± 2 km/h) | During | Ankle: 15 to 22 mmHg | Neither running economy nor kinematics demonstrated any difference between conditions at competition pace or at 105% of the best 10-km run, respectively. |
| Wang et al., 2016 | 12 track and field athletes, M, (21.2 ± 1.4 y) | Explore the influence of compression on (a) the muscle force and endurance of the quadriceps femoris by using a dynamometer and (b) the EMG amplitude and mean power frequency of the *rectus femoris*, *vastus lateralis*, and *vastus medialis* during repeated concentric muscle actions of the dominant leg. | EMG amplitude of the *rectus femoris, vastus lateralis, vastus medialis* 60 and 300˚/s isokinetic knee extensions | 25 consecutive maximal concentric muscle actions of the quadriceps at randomly ordered angular velocities of 60 and 300˚/s | During | NS | EMG amplitude was significantly lower in compression at 60 and 300˚/s. Additionally, EMG frequency of the *rectus femoris* and *vastus lateralis* was significantly higher in compression at 60˚/s |
| Wannop et al., 2016 | 10 competitive recreational athletes, M, (>18 y) | To determine how systematically increasing upper leg compression and hip joint stiffness independently from one another affects vertical jumping performance | Passive hip moment, peak hip flexion angle, peak hip extension angular velocity, peak knee flexion angle, peak knee extension angular velocity, peak ankle dorsiflexion angle, and peak ankle plantar flex angular velocity. | Vertical jumping | During | NS | The increase in jump height for the compression apparel condition was due to increased hip joint ROM and a trend of increasing the jump time. |
| Zhang et al., 2019 | 16 right-side dominant participants, 8F and 8M, (25.5 ± 2.6 y) | To determine the effects of compression garment position on healthy adults’ knee joint position sense acuity | Joint repositioning | The dynamometer was programmed to move the participant’s leg attached to the lever arm passively at 4°/s toward the target angle, which was then held for 5 s before the dynamometer’s lever arm with the subject’s leg attached to it, returned to the initial starting position. Following a 5 s interval the participant attempted to actively reposition the leg at the same joint angle | During | NS | Subjects had less joint repositioning error in the below knee condition when compared to control. No differences were observed between dominant or non-dominant legs |
| Zamporri and Aguinaldo, 2018 | 23 college athletes, F, (19.6 ± 1.3 y) | To explore the effects of compression garments on lower body 3D motion, specifically investigating both kinematic and kinetic changes at the knee and hip joints during a drop vertical jump in female collegiate athletes | Hip frontal-plane ROM, knee frontal-plane ROM, peak hip abduction, peak knee valgus, peak hip abduction moment, and peak knee valgus moment | 2 sets of 3 drop vertical jumps | During | NS | A significant reduction in frontal-plane hip motion as a result of wearing compression garments in female collegiate athletes performing a drop vertical jump |
| Zhang et al., 2016 | 12 track and field athletes, M, (21.2 ± 1.4 y) | Determine effectiveness of thigh compression apparel on 1) the force and endurance of the quadriceps and 2) the EMG amplitude and mean power frequency of the *rectus femoris*, *vastus lateralis*, and *vastus medialis* during repeated concentric muscle actions of dominant leg. | EMG of the *rectus femoris, vastus lateralis,* and *vastus medialis* | 25 maximal repeated isokinetic knee extensions at 60 and 300˚/s on a dynamometer | During | NS | Wearing a compression garment decreased the EMG amplitude and increased the EMG frequency |

M = Male, F = Female, ROM = Range of motion; GRF = Ground reaction force, NS = Not-specified, EMG = Electromyography, MVIC = Maximal voluntary isometric contraction, ACL = Anterior cruciate ligament, DOMS = Delayed-onset muscle soreness, MMG = Mechanomyography, H-reflex = Hoffmann reflex.
