# Supplementary material for: Putting the Squeeze on Compression Garments: Current Evidence and Recommendations for Future Research: A Systematic Scoping Review
Source: Sports Med. 2021 Dec 6;52(5):1141–60. doi: 10.1007/s40279-021-01604-9 (PMC9023423; doi:10.1007/s40279-021-01604-9)
Supplement: Supplementary file 3 — Supplementary file3 (DOCX 91 kb) [file 40279_2021_1604_MOESM3_ESM.docx]

**Supplementary Table S3.** Details of studies and information relevant to blood and salivary outcomes.

| **Study** | **Cohort/ sample size, sex, age** | **Study purpose** | **Outcome Measures** | **Exercise Protocol** | **Compression worn during/after/both** | **Compression pressure – reported value or not stated** | **Key findings** |
| --- | --- | --- | --- | --- | --- | --- | --- |
| Ali et al., 2011 | 12 well-trained runners, 3F and 9M, (33 ± 10 y) | Examine the effects of wearing different grades of graduated compression garments on 10-km running performance | La | 10km running time trial | During | Low condition: Ankle 15 mmHg Knee 12 mmHg  Medium condition:  Ankle: 21 mmHg Knee: 18 mmHg   High condition:  Ankle: 32 mmHg Knee: 23 mmHg | There was no effect of wearing different graduated compression garments on La when compared with a non-CG control |
| Ali et al., 2010 | 10 triathletes, 1F and 9M, (36.0±10.0y) | To examine the physiological and perceptual responses to wearing graduated compression stockings during fast-paced running | La, CK and Mb | 90% of 10 km personal best speed at 1% incline for 40 mins on treadmill | During | Low CG: 12 - 15 mmHg  High CG: 23 - 32 mmHg | Runners experienced no benefits while wearing CG on blood markers of muscle damage |
| Areces et al., 2015 | 34 marathon runners, 4F and 30M,  Control group: (42.7 ± 7.8 y), Compression group: (41.2 ± 8.9 y) | Investigate the benefits obtained by wearing graduated compression stockings on running pace, prevention of muscle damage and maintenance of muscle performance during a real marathon race | Sodium, chloride, potassium, calcium, Mb, CK, LDH, enzymes and blood oxygen saturation | Marathon race | During | The highest pressure was at the foot and the malleolus, and it decreased proximally, from 25 mmHg to 20 mmHg | Blood markers were the same in the compression group and control group |
| Argus et al., 2013 | 11 highly trained cyclists, M, (31 ± 6 y) | To evaluate the use of 3 recovery strategies compared with a passive control on repeated sprint-cycling performance in highly trained cyclists | La | 3 maximal 30 second sprint separated by 20 min of recovery with compression garment | Between maximal sprints | Lower calf: 27 ± 6 mmHg Upper thigh: 18 ± 2 mmHg | Compression had little benefit in decreasing La following maximal sprinting |
| Beaven et al., 2013 | 16 professional rugby players, M, (25 ± 3 y) | To assess the effectiveness of compression garments and an electrostimulation device at assisting recovery of professional rugby players during a preseason training period. | Saliva: Cortisol and testosterone levels, CK. | Preseason training | During | NS | The combination of an electrostimulation device with a compression garment had no effect on saliva or blood markers. |
| Berry & McMurray, 1987 | Experiment 1: 6 fit college students, M,  (22.5 ± 5.4 y)  Experiment 2: 6 healthy college students, M, (21.4 ± 4.3 y) | The first study was designed to determine the effects of GCS on maximal oxygen consumption, time to exhaustion during a test for VO_2max_ and blood lactate levels during recovery from a test of VO_2max_. The second study was designed to evaluate retention of lactate by manipulation of the CG at the end of exercise. | La | 15 min at treadmill speed set to elicit a heart rate of 130 bpm, after which time the grade was increased every 2 min by 2.5% until exhaustion | During | Ankle: 18 mmHg Calf: 8 mmHg | There was a decrease in venous La concentration while wearing CG during both exercise and recovery. |
| Bieuzen et al., 2014 | 11 highly trained runners, M, (34.7 ± 9.8 y) | To examine the effect of wearing compression stockings on indices of EIMD during trail-running. Compression stockings were worn either during or after a trail run performed at competition pace by experienced off-road runners | CK, IL-6 | The simulated trail race consisted of 3 laps of 5.2 km (total distance: 15.6 km) in mountainous terrain. | During | Running condition: 25 mmHg Recovery condition: 20 mmHg | Compression had little benefit in decreasing blood markers following trail running |
| Born et al., 2014 | 10 elite German ice speed skaters, M, (23 ± 7 y) | To investigate whether the application of compression improves muscle oxygenation and blood volume, ratings of perceived exertion; blood lactate concentration; and, 3000-m time. | La | 3000-m race simulation | During | Thigh 20.3 ± 2.3 mmHg Calf: 24.4 ± 3.1 mmHg | Compression provided no effect on blood La |
| Born et al., 2014 | Sub-study 1:  12 track and team sport athletes, F, (25.0 ± 3.0 y); Sub-study 2: 12 track and team sport athletes, F,  (23 ± 2 y) | There were 2 aims; 1) To assess the effects of compression garments with silicone stripes (which mimic kinesio taping) on repeated sprint performance; 2) to identify the physiological, biomechanical, and perceptual effects of compression garments with silicone stripes | La | 30 x 30-m sprints (one sprint per minute) | During | Entire lower body: ~18 – 20 mmHg | La level unaffected by compression during 30 x 30 m sprints |
| Broatch et al., 2017 | 20 recreationally active, 11F (25 ± 2 y) and 9M (28 ± 6 y) | This study aims to assess the effects of lower-limb compression garments on markers of blood flow, oxygen kinetics, and exercise performance during a repeated sprint protocol with short rest intervals | La, pH, bicarbonate, base excess | 4 sets of 10 x 6-s maximal sprints, inter-spaced by 24 s of recovery between bouts and 2 min recovery between sets | During | Thigh: 11.7 ± 2.3 mmHg  Calf: 26.4 ± 6.4 mmHg  Ankle: 21.5 ± 8.2 mmHg | Lower-limb compression garments worn during RSE had no impact on blood markers |
| Broatch et al., 2019 | 12 elite Australian volleyball athletes, F, (25 ± 2 y) | To determine the effects of wearing compression socks during long-haul travel on sports-specific performance, physiological, and haematological alterations in elite female volleyball athletes | Tissue factor pathway inhibitor, thrombin-antithrombin complex, and D-Dimer | Air travel | Worn during travel | Max calf girth: 23 ± 11 mmHg  Ankle: between 19 and 22 (±8) mmHg | Compression had no effect on indicators of blood coagulation. |
| Brophy-Williams et al., 2016 | 12 well trained runners, M, (30.5 ± 8.1 y) | To assess the effect of wearing compression socks during a one-hour recovery period following a 5km running time trial on performance in a subsequent 5km time trial | La | A 5-km TT, then a 1-hour recovery intermission before a second warm-up and 5-km TT. | Between time trials | Max calf girth: 23 ± 11mmHg  Upper ankle: 22 ± 8mmHg  Lower ankle: 19 ± 8mmHg | Compression had no benefit on La following 5km TT |
| Brophy-Williams et al., 2018 | 12 runners, M, (30.5 ±8.1 y) | Assess the effect of wearing compression socks during a 5km running time trial on physiological, perceptual and performance-based parameters, including subsequent performance. | La | A 5 km TT, a 1-hour recovery period, then a repeat of the warm-up and 5 km TT | During the first warm up and time trial. | Max calf girth: 37 ± 4 mmHg  Upper ankle: 31 ± 4 mmHg  Lower ankle: 23 ± 4 mmHg | No difference of compression on La |
| Brown et al., 2020 | 45 Recreational rugby players, M, 13 custom fit (24.0 ± 6.0 y), 16 standard size (23.0 ± 3.0 y) and 16 control (22.0 ± 4.0 y) | To evaluate the effects of compression garments applying different pressures on muscular recovery after EIMD in rugby players | CK | 20 x 20m sprints, 100 drop jumps | 48 hours post exercise | Custom fit  Ankle: 32 ± 3 mmHg  Calf: 24 ± 4 mmHg  Thigh: 19 ± 3 mmHg  Standard size  Ankle 11 ± 5 mmHg  Calf: 10 ±3 mmHg  Thigh: 7 ± 3 mmHg | Custom-fitted CG designed to apply higher pressures than commercially available garments were associated reduced levels of CK |
| Chatard et al., 2004 | 12 trained cyclists, M, (63 ± 3 y) | To determine whether compression stockings affect performance recovery and leg pain following maximal exercise. | La, Hct | All out five-minute cycloergometer followed by 80-min rest and then another all out five minutes | For 80 minutes between maximal attempts | The average pressure exerted by the CG, was 44 hPa at the ankle, 24 hPa at the calf, and 17 hPa at mid-thigh which represented a 40% pressure decrease between the ankle and the mid-thigh – manufacturer reported | Compression stockings decreased La and haematocrit, and increased La removal measured during the recovery period |
| Choi et al., 2019 | 38 baseball players, M, (19.6 ± 0.2 y) | To examine the effects of wearing nanodiamond- and nanoplatinum-coated fibre garments at night on the physical and psychological stress of Japanese male baseball players induced by strenuous training over a two-week period | salivary cortisol | Strenuous training over a two-week period | Participants were asked to wear the garments before bedtime and for at least 10 h (9:00 pm–07:00 am) every night for two weeks | NS | Nanodiamond- and nanoplatinum-coated fibres in compression garments worn at night significantly decreased salivary cortisol levels |
| Dascombe et al., 2011 | 11 well-trained middle-distance runners and triathletes, M, (28.4 ± 10.0 y) | To determine the effectiveness of wearing an undersized lower body compression garment on physiological and performance parameters relating to endurance running. | La | Time to exhaustion test consisting of the participant running at 90% of VO_2max_ velocity until volitional exhaustion | During | Regular size:  Thigh: 13.7 ± 2.3 mmHg Calf: 19.2 ± 3.2 mmHg  Undersize Thigh: 15.9 ± 2.6 mmHg Calf: 21.7 ± 4.3 mmHg | No effects of compression on La |
| Dascombe et al., 2013 | 7 elite flat-water kayakers, 2F (25.0 ± 4.2 y) and 5M (21.8 ± 2.8 y) | To determine the effects of wearing upper body compression garments on performance during simulated flat-water kayaking and physiological responses during simulated kayaking | La | Participants completed a six-step incremental test and a subsequent 4min performance test on a kayak ergometer | During | NS | The data demonstrated no significant improvements in La measures during simulated flatwater kayaking in elite kayakers wearing the CG |
| Davies et al., 2009 | 7 female netball players and 4 male basketball players, 7F (19.7 ± 0.5 y) and 4M (26.3 ± 5.1 y) | To investigate whether wearing compression tights for 48 hours following plyometric exercise would attenuate muscle damage markers and muscle soreness, and sprinting and jumping performance | CK, LDH | 5 sets of 20 drop jumps from a platform 60 cm high followed immediately by a maximal upward jump, with a 2-minute rest period between sets | For 48 hours afterwards | Graduated pressure of ~15 mmHg from the lower to the upper legs | Compression tights during recovery reduced CK levels in females only, but no effects of compression on LDH |
| Del Coso et al., 2013 | 36 experienced triathletes, Sex NS, Control group, n=17, (35.8 ± 6.3 y),  Compression group, n=19, (35.0 ± 5.3 y) | To investigate the potential of compression stockings to prevent muscular damage and to preserve muscular performance during a half-ironman competition. | Blood osmolality, Glucose, Sodium, Chloride, Potassium, Calcium, Mb, CK | Half-iron man | During | NS | Triathletes wearing compression stockings presented comparable blood biomarkers with the control group |
| Driller and Halson 2013 | 12 highly trained male cyclists, M, (30 ± 6 y) | To investigate the effect of wearing lower body compression garments on performance during a 30-minute endurance cycling test. A further aim of the study was to determine various physiological and perceptual responses when wearing compression garments during the cycle test. | La | 15 minutes at a workload equal to 70% PPO, followed immediately by a 15-minute time trial. | During | ~18 mmHg at the *medial malleolus* decreasing to ~10 mmHg at the *gluteus maximus* | Wearing lower body compression garments during a 30-minute cycling performance test resulted in a lower La |
| Duffield and Portus, 2015 | 10 club cricket players, M (22.1 ± 1.1 y) | To compare the effects of three different types of full-body compression garments and a control condition on performance in intermittent, repeat-sprint and throwing performance in cricket players. | La, pH, oxygen saturation of Hb, partial pressure of oxygen, CK | 30 min repeat-sprint exercise protocol comprising 20 m sprints every minute, separated by submaximal exercise. Throwing tests included a pre-exercise and a post-exercise maximal distance test and accuracy throwing tests. | During and 24 hours after | NS | Reduced CK values 24 h after exercise in the compression garment condition.  No effects of compression on La, pH or oxygen saturation of Hb. |
| Duffield et al., 2008 | 14 rugby players, M, (19 ± 1 y) | To determine whether compression garments improve intermittent-sprint performance and aid performance or self-reported (perceptual) recovery from high-intensity efforts on consecutive days | La, CK | 4 x 15-min quarters of a simulated team game (exercise circuit) repeated across two days | During the simulated team games and for ~15 hours afterwards | NS | Compression had little benefit in decreasing biomarker concentrations |
| Duffield et al., 2010 | 11 rugby players, Sex NS, (20.9 ± 2.7 y) | Examine the effect of wearing compression garments during and 24 h following high-intensity, intermittent-sprint and stretch shortening cycle activities on post-exercise performance and recovery of evoked and voluntary muscle performance. | La, pH, aspartate transaminase, CK, CRP | 10-min exercise protocol of a 20-m sprint and 10 plyometric bounds every min | During and for 24 hours after | NS | Compression had little benefit in decreasing biomarker concentrations after high-intensity, intermittent-sprint and stretch shortening cycle activities |
| Duffield et al., 2014 | 8 professional tennis players, M, (20.9 ± 3.6 y) | To investigate the effects of combining cold water immersion, compression garments, and sleep-hygiene recommendations on physical, physiological, and perceptual recovery after 2-a-day on-court training and match-play sessions | Lactate | Each respective on-court session involved 90 minutes of coach-led drills (including a 30-min warm-up) and 90 minutes of competitive match play | Between drill and match play (~3 hours) and for 4 hours match play | NS | The combined use of cold-water immersion and compression garments had no effect on La. |
| Faulkner et al., 2013 | 11 trained runners, M (23.7 ± 5.7 y) | To examine the effects of lower-limb compression on 400-m run performance and physiological and perceptual indicators of intensity and performance | La | 400m sprint | During | Long garment: 2.0 - 13.2 mmHg  Short length garment: 3.7-20.7 mmHg | There were no significant differences in blood lactate profiles between conditions. |
| Ferguson et al., 2014 | 21 healthy participants, M,  (21 ± 1 y) | To examine the effects of neuromuscular electrical stimulation, compared to graduated compression socks on muscle soreness, strength, and markers of muscle damage and inflammation following intense intermittent exercise | CK, LDH, IL-6, CRP | Two 45-min sections of continuous intermittent exercise separated by a 15-min rest period.  3 × 20 metres at walking pace, 1 × 20 metre maximal sprint, 4 s recovery, 3 × 20 metres at a running speed corresponding to 75 % VO_2max_, 3 × 20 metres at a running speed corresponding to 100 % VO_2max_ | At least 12 h post the 1 h testing point | Ankle: 40 mmHg  Calf: 20 mmHg | Compression has no effect on blood markers of muscle damage. |
| French et al., 2008 | 26 participants, M, (24.12 ± 3.2 y) | To evaluate contrast bathing and contrast garments as regeneration strategies after EIMD | CK, Mb | 6 x 10 squats with 100% of body mass + 5 second eccentric repetition with the participants 1RM | For 12 hours after exercise | Calf: 12 mmHg Thigh:10 mmHg | Compression had little benefit in decreasing biomarker concentrations following resistance exercise |
| Gill et al., 2006 | 23 elite rugby players, M, (25.0 ± 3.0 y) | To investigate the effectiveness of four recovery interventions on CK recovery profiles in professional rugby players following competitive matches | CK | Rugby match | ~12 hours after | NS | Compression was a more effective post-match recovery intervention than passive recovery for reducing level of CK |
| Glanville and Hamlin 2012 | 14 trained multisport, M, (33.8 ± 6.8 y) | To determine the effects of wearing commercially available graduated compression garments during prolonged recovery (24hours) on subsequent 40-km cycling time trial performance in trained multisport athletes. | La | 40-km cycling performance | Post-exercise for 24 hours | Upper ankle: 6.0 ± 2.4 mmHg, Upper calf: 14.7 ± 2.5 mmHg, Upper leg segment: 11.8 ± 2.5 mmHg | Wearing a compression garment for a 24-hour recovery period between successive 40-km time trials had no influence on La |
| Goto and Morishima, 2014 | 9 participants, M, (21.0 ± 0.4 y) | To investigate the effects of wearing a compression garments for 24 h on the changes in muscular strength and blood parameters over time after resistance exercise. | Insulin-like growth factor-1, free testosterone. Mb, IL-6, IL-1 | Six exercises for the upper body and three for the lower body muscles. Each exercise set comprised 10 repetitions involving five sets for bilateral leg press and bilateral knee extension and three sets for the remaining seven exercises | Worn for 24 hours after resistance training | NS | Compression had no benefit on blood markers following full body resistance exercises |
| Goto et al., 2017 | 11 participants, M, (22.7 ± 0.9 y) | To determine the effect of compression garments during post-exercise periods after two repeated bouts of exercise on exercise performance, muscle damage, and inflammatory responses | La, Glucose, Mb, CK, IL-6, Leptin | Repeated sprint cycling and resistance exercise | The subjects wore the prescribed garments throughout the whole recovery period (4 h after experiment 1 and approximately 18 h after experiment 2), except during two repeated bouts of exercise (60 min for each exercise). | Thigh: 11.5 ± 0.6 hPa  Calf: 17.6 ± 1.8 hPa | Compression had no benefit on blood markers following repeated sprint exercise |
| Govus et al., 2018 | 32 cross country national and junior level skiers, Seniors  9F (23.2 ± 2.6 y) and  12M (25.2 ± 3.6 y),  Juniors  5F (18.2 ± 0.8 y) and  6M (18.0 ± 0.6 y) | To determine whether compression garments and neuromuscular electrical stimulation accelerated the recovery of blood biomarkers of muscle damage, countermovement jump height and perceived muscle pain before and 8, 20, 44 and 68 h after a cross-country sprint skiing competition in a cohort of elite senior and junior cross-country skiers. | CK, urea | Cross-country sprint skiing competition | For ∼17 h following the post-competition period. | Lower-body compression: Ankle: 14.6 ± 0.1 mmHg *Achilles tendon*: 13.6 ± 1.4 mmHg Calf: 13.7 ± 1.3 mmHg T*ibial tuberosity*: 7.6 ± 1.1 mmHg P*atella*: 8.3 ± 2.3 mmHg Mid-thigh: 5.3 ± 1.1 mmHg 5 cm below crotch: 4.2 ± 2.8 mmHg | Compression had no benefit on blood markers following cross country sprint skiing |
| Hamlin et al., 2012 | 22 rugby union players, M, (20.1 ± 2.1 y) | To determine the effects of wearing either a compression garment or placebo garment over a 24-hour recovery period on subsequent physiological and performance measures | CK, La | A series of exercise circuits designed to simulate a game of rugby | After | Compression:  *Sphyrion*: 8.6 ± 2.6 mmHg Mid-calf: 13.4 ± 2.0 mmHg M*id-trochanterion*: 9.0 ± 2.2 mmHg  Control:  *Sphyrion*: 2.6 ± 1.2 mmHg, Mid-calf: 5.0 ± 1.5 mmHg,  *Mid*-*trochanterion*: 3.5 ± 0.9 mmHg | Compression had little benefit in decreasing biomarker concentrations following a simulated rugby game |
| Heiss et al., 2018 | 15 participants, 7F and 8M (25 ± 6 y) | To investigate the influence of commercially available sport compression garments on the development of exercise-induced intramuscular oedema | CK | All participants performed 5 sets of 30 repetitions of calf raises and rested 10 sec between each set with 25% of their body weight during the exercise | The compression sock was worn continuously for 60 h after eccentric exercise and was removed for the first time for follow-up examination | NS | Wearing conventional sports compression garments during the recovery phase had no significant effect on reducing CK |
| Hettchen et al., 2019 | 19 handball players, M, (31.3 ± 7.7 y) | To determine the effect of compression tights on relevant parameters of recovery | CK, Mb | Two sets of 8-10 repetitions of lunges, unilateral calf raises, and squats were prescribed. Exercise to failure per exercise in the range of 8-10 reps. 60 seconds recovery between exercises | Compression was applied initially for 24 h and then 12 h intermitted by 12 h of non-use for a total of 96 h | 19.0 - 26.2 mmHg for the onset of the calf muscle; 16.3-23.5 mmHg for the highest calf circumference; 9.9 - 18.1 mmHg two fingerbreadths beneath the *fossa popliteal*; 7.7 – 14.3 mmHg at the mid-knee; 9.9 - 13.9 at mid-thigh and 8.0 - 12.2 mmHg for the region two fingerbreadths beneath the crotch | Compression had no benefit on blood markers following lower body resistance exercise |
| Higgins et al., 2009 | 9 Netballers, F, (22.6 ± 4.6 y) | the purpose of this study was to examine effectiveness of compression garments on physiological and performance markers in a game-specific circuit for netball. | La | A circuit designed to simulate competitive netball. 4 x 15 min circuit per session to simulate the four quarters of a competitive game. The circuit comprised of six stations simulating game conditions that were repeated throughout each quarter | During | NS | No interactive effect of compression garments La. |
| Hill et al., 2014 | 24 recreational marathon runners, 7F and 17M, Compression group (47.7 ± 10.8 y),  Sham ultrasound (41.1 ± 10.5 y) | To investigate the effects of wearing a commercially available, lower limb, compression garment on the recovery of strength, soreness and indices of muscle damage following a marathon run. | La, CK, CRP | Marathon run | For 72 hours after exercise | Between 9.9 - 24.4 mmHg | No effect of compression on La, CK or CRP |
| Hill et al., 2017 | 45 recreationally active participants, 19F and 26M, Low pressure group  (29.2 ± 4.7 y),  High group (32.7 ± 7.8 y),  Sham group (28.3 ± 4.1 y) | To assess whether garments exerting a higher degree of pressure are more effective in facilitating recovery compared to garments exerting a lower pressure | CK, CRP, Mb | The muscle damaging protocol consisted of 100 drop jumps from a 0.6 m platform. Participants performed 5 sets of 20 drop jumps, with 10 seconds between each jump and a 2 min rest period between sets | For 72 hours post exercise | Low condition Thigh: 8.1 ± 1.3 mmHg  Calf: 14.8 ± 2.1  High condition Thigh: 14.8 ± 2.2 mmHg  Calf: 24.3 ± 3.7 mmHg | Compression had no benefit on blood markers following drop jumps |
| Houghton et al., 2007 | 12 trained amateur field hockey players,  M, (21[19-23] y) | To investigate the effects of compression garments on thermoregulation in field hockey players. | La | The Loughborough intermittent shuttle test | During | NS | La concentrations were not different between trials |
| Jakeman et al., 2010 | 32 physically active participants, F, (21.4 ± 1.7 y) | To determine whether a combined treatment involving sports massage and compression immediately after damaging exercise was an effective strategy to manage the symptoms of EIMD induced by strenuous plyometric exercise | CK | 10 x 10 plyometric drop jumps from a 0.6-m box. One minute rest between sets | For 12 hours post-exercise | Calf: 17.3 mmHg  Thigh 14.9 mmHg | Compression had little benefit in decreasing biomarker concentrations following plyometric drop jumps |
| Jakeman et al., 2010 | 17 physically active participants, F, (21.4 ± 1.7 y) | To investigate the efficacy of complete lower limb compression clothing on recovery from the symptoms of EIMD following strenuous plyometric activity | CK | 10 x 10 plyometric drop jumps from a 0.6-m box. One minute rest between sets | For 12 hours post-exercise | Calf: 17.3 mmHg Thigh: 14.9 mmHg | Compression had little benefit in decreasing biomarker concentrations following plyometric drop jumps |
| Kemmler et al., 2009 | 21 moderately trained runners, M, (39.3 ± 10.7 y) | To determine the effect of below-knee stockings with constant compression on selected parameters of running performance in healthy male runners | La | Stepwise-speed incremental running test | During | Ankle: ~24 mmHg, Calf: ~18 - 20 mmHg | Compression stockings were not effective for reducing La during submaximal and maximal running exercise. |
| Kim et al., 2017 | 16 participants, M, 8 control, (23.13 ± 3.76 y), 8 compression, (24.25 ± 1.28 y) | To investigate wearing compression garments after eccentric exercise using elbow flexor and effects on DOMS and inflammatory response | CK, TNF-α | Each eccentric muscle contraction was performed for 3 sec. Each participant completed two sets, performing 25 reps per set with a 5-min rest period between sets | 24 hours after exercise | 5–10 mmHg | Compression garments effectively did not cause statistically different effects on CK or TNF- α |
| Kraemer et al., 2001 | 15 healthy non-strength-trained, M  Compression group, 8 M, (22.3 ± 2.9 y)  Control group, 7M, (22.1 ± 3.3 y) | To determine whether a compression sleeve worn immediately after maximal eccentric exercise enhances recovery | CK | 2 isokinetic dynamometer set for passive motion at 60°/s. 2 x 50 rep with 3 min rest between sets. Every 4^th^ rep a maximal concentric contraction with an isometric hold at end range followed by an eccentric contraction | After | NS | Compression sleeves attenuated rise of CK levels post exercise |
| Kraemer et al., 2001 | 20 non-strength-trained participants, F,  Compression sleeve group (21.3 ± 2.9 y),  Control group,  (21.1 ± 3.3 y) | To investigate whether constant compression via the use of a compressive arm sleeve would reduce the severity and duration of soreness associated with DOMS. | CK, cortisol, LDH, | Two sets of 50 repetitions on a dynamometer (60°/s) with 3 minutes rest between sets. Every fourth repetition, the subject performed a maximal concentric contraction with an isometric hold followed by an eccentric contraction | After | 10 mmHg | Compression sleeves attenuated rise of CK levels post exercise but had no impact on blood biomarkers |
| Kraemer et al., 2010 | 20 resistance trained subjects, 9F (23.1 ± 2.2 y) and 11M (23.0 ± 2.9 y) | To evaluate the influence of a whole-body compression garment on recovery from a typical heavy resistance training workout | .  CK, LDH, clinical chemistries. | Three sets at 8-10RM of back squats, bench press, stationary lunge, bent-over row, Romanian dead lift, biceps curl, sit up, high pull from hang. | For 24 hours after exercise | NS | Whole-body compression garment did produce more rapid recovery of CK and LDH when compared with the use of a non-compression treatment condition over a 24-hour recovery time frame. No effects of compression on clinical chemistries |
| Kraemer et al., 2016 | 19 recreationally trained, M  Control group, 9M, (23.2 ± 2.3 y)  Full body compression group, 10M, (23.1 ± 2.4 y) | To examine the impact of trans-American jet travel on physical performance and associated hormonal and sleep-related responses to gain insight into potential mechanistic contributions to any reductions in physical performance. Secondarily, to assess the impact of a return flight on recovery processes following a demanding physical activity and whether a compression garment intervention could ameliorate any of the tissue damage upon return to the original time zone | melatonin, epinephrine, norepinephrine, myoglobin, creatine kinase, total testosterone, cortisol. | Roundtrip trans-American jet travel | During | NS | The compression group demonstrated significant attenuations in markers of muscle damage immediately following the simulated event, as well as immediately following the eastbound return trip, and 24 h following arrival on the east coast of the USA. |
| Kumstát et al., 2018 | 8 physically active, M, (27.1 ± 2.3 y) | To investigate the comparative effect of using compression calf sleeves with active recovery and passive rest on immediate recovery and high intensity repeated cycling performance | La, acid-base balance | Three maximal bouts (30-20-10 s; the external loading was set at 7.0% of the individual’s body mass). 3 min recovery between efforts. | For 24 minutes between maximal effort bouts. | Ankle: 25 mmHg Calf: 21 mmHg | Compression had no benefit on blood markers following maximal cycling |
| Kupchak et al., 2016 | 19 resistance trained, M  Control group, 9M, (23.2 ± 2.3 y)  Full body compression group, 10M, (23.1 ± 2.4 y) | Determine the coagulant and fibrinolytic responses from a full-body, muscle-damaging workout followed by a transcontinental flight in physically active young adults | tissue Plasminogen Activator, Plasminogen Activator Inhibitor, D-Dimer, activated Partial Thromboplastin Time, Thrombin–Antithrombin Complex, Prothrombin Fragment, Mb, CK | Transcontinental flight | During | NS | Wearing a full-body compression garment limited the degree of coagulation post-exercise and post-flight. |
| Leicht et al., 2020 | 30 active participants, 15F and 15M, (19.8 ± 1.9 y) | To examine the impact of lower body compression garments on cardiac autonomic control of heart rate prior to, during and following submaximal exercise. | La | Cycling at a moderate intensity equivalent to 70% age-predicted maximum heart rate | During and after | NS | Minimal impact of compression garments on La during and following submaximal exercise in healthy adults |
| Leoz-Abaurrea et al 2016 | 10 recreational runners, M, (23.0 ± 3.0 y) | To analyse the physiological responses of heat dissipating upper body compression garments during a running performance test to exhaustion | La | 45-min run at 60% of the peak treadmill speed followed by a TTE run at 80% of the peak treadmill speed | During | *Bicep brachii*: 2.9 ± 1.5mmHg *Triceps*: 3.0 ± 1.0 mmHg *Pectoralis major*: 2.0 ± 0.5 mmHg *Latissimus dorsi*: 1.4 ± 0.5mmHg | Blood La was higher during recovery in the compression trial |
| Leoz-Abaurrea et al 2016 | 16 untrained participants, 4F and 12M, (21.3 ± 5.7 y) | To determine the effects of upper body compression garments on thermoregulatory responses during cycling in a controlled laboratory thermoneutral environment (~23°C). A secondary aim was to determine the cardiovascular and perceptual responses when wearing the garment. | pH, Hct, Na+ plasma, and K+ plasma | Cycling at a fixed workload (~50% VO_2peak_) with four bouts of 14 minutes at 40 ºC with each bout separated with a minute active recovery | During | NS | Wearing an upper body compression garment had no effect on biomarkers in a thermoneutral environment when compared to a similar control garment. |
| Lovell et al., 2011 | 26 semi-professional rugby league players, M, (21.6 ± 2.5 y) | To examine the effect of compression garments on active post-exercise recovery after a bout of high-intensity exercise | blood pH, La, | A 6-stage submaximal treadmill test which consisted of 5-minute stages at 6 km/h, 10 km/h, approximately 85% of VO_2max_, and 6 km/h as a recovery stage followed by approximately 85% of VO_2max_ and 6 km/h | During | Ankle: 20 ± 2 mmHg  Calf: 15 ± 2 mmHg | Compression garments reduced La during recovery and at 10km/h during submaximal treadmill running.  No effects of compression on pH |
| Marqués-Jiménez et al., 2017 | 18 semi-professional football players, M, (24.7 ± 4.07 y) | To evaluate the influence of wearing different types of compression garments during matches and recovery after a friendly soccer match | CK, LDH, glutamic oxalacetic transaminase, glutamic–pyruvic transaminase, gamma glutamyl transpeptidase, | Soccer match | Each participant in the experimental condition played the match wearing one type of graduated compression garment (Compressport®, Genève, Switzerland) and kept wearing them 7 h/day during 3 days post-match (players put them on each day after the testing session). | Compression stockings:  Ankle: 20–25 mmHg  Calf: 15–20 mmHg    Compression tights: Calf: 25–30 mmHg  Thigh:15–20 mmHg  compression shorts: Thigh: 15 – 20 mmHg | There is a positive but not significant effect of compression garments on attenuating CK, glutamic oxalacetic transaminase and gamma glutamyl transpeptidase, responses 72 h post-match.  CG had a trivial relative effect attenuating LDH increments during recovery  No effect of compression on glutamic–pyruvic transaminase |
| Marqués-Jiménez et al., 2017 | 18 semi-professional football players, M, (25.2 ± 3.0 y) | To evaluate the influence of wearing different types of compression garments during matches and recovery after a friendly soccer match | La, arterial oxygen sat of Hb | Soccer match | Each participant in the experimental condition played the match wearing one type of graduated compression garment and kept wearing them 7 h/day during 3 days post-match | Compression stockings: Ankle: 20–25 mmHg  Calf: 15–20 mmHg  Compression tights: Calf: 25 – 30 mmHg at calf, Thigh: 15–20 mmHg  Compression shorts: Thigh: 15–20 mmHg | Using compression garments slightly increased La during and after soccer matches |
| Martínez Navarro et al., 2020 | 32 recreational ultra-endurance athletes, 13F and 19M, (41 ± 6 y) | To analyse the effect on DOMS, muscle damage, inflammatory response, and renal function of wearing a full-body compression garment for 24 h immediately after a 107-km ultra-trail | Creatinine, glomerular filtration rate, CK, LDH, CRP | 107km Ultra trail race | Worn for 24 h post | 10–15 mmHg | Full body compression garments are not useful for reducing muscle damage and inflammatory response after an ultra-trail race |
| Martorelli et al., 2015 | 15 resistance trained, M, (23.1 ± 3.9 y) | To examine the effects of upper-body graduated compression sleeves on neuromuscular and metabolic responses during power training. | La | Repetitions to failure test were performed with 50% of 1RM. | During | NS | No effects of compression on La. |
| Mizuno et al., 2016 | 18 participants, M, (21.9 ± 0.6 y) | To determine the effects of wearing a lower body compression garment for 24 h following running (either downhill or level) in terms of recovery of exercise performance, muscle damage, inflammatory markers in the blood, and subjective muscle soreness and fatigue. | Blood glucose, La, CK, Mb, cortisol, CRP, IL-6 | 30 minutes of downhill running | 24 hours post exercise | Compression group: Thigh:11.5 ± 0.6 hPa Calf: 17.6 ± 1.8 hPa  Control group:  Thigh: 7.1 ± 1.3 hPa,  Calf: 11.5 ± 2.1 hPa | Compression had no benefit on blood markers following 30 min of downhill running |
| Mizuno et al., 2017 | 30 physically active participants  Compression thigh group, 10M,  (21.3 ± 0.4 y)    Compression sock group 10M, (21.6 ± 0.8 y)  Control group, 10M, (22.9 ± 0.7 y) | Examine the effects of the body coverage area of compression garments on the exercise performances and muscle damage during prolonged running | Blood glucose, La, Mb, free fatty acids, CRP, IL-6 | 120min of uphill running at 55% of ˙VO_2max_ | During | Compression group Thigh: 14.7 ± 0.6 mmHg  Calf: 17.4 ± 0.5 mmHg  Control group:  Thigh: 3.0 ± 0.3 mmHg Calf: 1.8 ± 0.2 mmHg | Increase in Mb concentration induced by prolonged running was significantly attenuated with thigh compression, with no influence of compression on glucose, free fatty acid, CRP or IL-6. |
| Mizuno et al., 2017 | 8 participants, M, (23.4 ± 2.4 y) | To investigate the effect of wearing lower body compression garments exerting different pressure levels during prolonged running on exercise-induced muscle damage and the inflammatory response. | blood glucose, IL-6 La, CK, Mb | 120 min of uphill running at 60% of VO_2max_ | During | High pressure garment:  Thigh: 26.9 ± 3.3 mmHg Calf: 29.2 ± 3.8 mmHg  Medium pressure garment:  Thigh: 16.1 ± 2.0 mmHg  Calf: 17.9 ± 3.5 mmHg  Control garment:  Thigh: 4.4 ± 1.2 mmHg  Calf: 3.0 ± 1.6 mmHg | Increased plasma IL-6 concentration during 120 min of running was also less in the medium pressure garment trial than in the control trial, with no effect of compression on other blood markers |
| Montgomery et al., 2008 | 29 basketball players, M, (19.1 ± 2.1 y) | To (1) investigate the time course of muscle damage markers and inflammatory cytokines during basketball tournament play and (2) assess whether cold water immersion and compression recovery strategies ameliorate any post-game increases of these biomarkers, compared with traditional refuelling and stretching routines | Mb, IL-6, IL-10, CK, Fatty-acid binding protein | 3-day mini-tournament involving one full 48 min game per day | For ~18 hours post-game | ~18 mmHg | Compression had little benefit in decreasing biomarker concentrations measured 6 h after a game |
| Okamoto et al., 2012 | 10 healthy participants, M (29.8 ± 5.9 y) | Investigate the acute effect of brisk walking with and without graduated compression garments on vascular endothelial function and oxidative stress | La. Derivatives of reactive oxygen metabolites, biological antioxidant potential. | Walking on a motor-driven treadmill at 5.9–6.7 km/h for 30 min at an intensity of ~60% of heart rate reserve | During | Ankle: 25 mmHg  Calf: 17 mmHg | No effects of compression on La or derivatives of reactive oxygen metabolites.  CG increases biological anti-oxidant potential. |
| Pereira et al., 2014 | 22 resistance trained participants, M, (24.6 ± 5.1 y) | To examine the effect of graduated compression sleeves worn during exercise on muscle recovery in young resistance trained men. | CK | Four sets of 10 maximal elbow flexion/extension at 120°/s. 1 minute separated sets | During | NS | No significant differences between groups for CK levels across 96 hours post exercise. |
| Pruscino et al., 2013 | 8 highly trained hockey players, M, (21.9 ± 2.3 y) | To investigate the efficacy of wearing a full-length, lower-body compression garment following a hockey-simulation and post-exercise biochemical response and recovery of muscle function | La, IL1-β, IL-6, TNF- α, CRP, CK | Hockey simulation protocol | 24 hours after | Ankle:19.1 mmHg Calf: 7.2 mmHg Thigh: 4.9 mmHg. | None of the blood markers of recovery indicates any benefit of wearing compression garments post-exercise. |
| Rider et al., 2014 | 10 runners,  3F (18.7 ± 0.6 y) and 7M (21 ± 1.3 y) | To determine whether wearing below-the-knee graduated compression stockings e during a maximal treadmill run would induce physiological changes among collegiate cross-country runners. | La | 5-km running time trial | During | Ankle: 20 mmHg Calf: 15 mmHg | Wearing below knee CG during a maximal treadmill run had no influence on La |
| Rimaud et al., 2010 | 8 endurance trained participants, M, (27.1 ± 0.9 y) | To investigate effects of wearing compression stockings during exercise | La | Volitional test to exhaustion | During and 60 min after | Maximum of 22 mmHg at the calf and 12 mmHg at the ankle | Compression during exercise lead to higher La values  Compression during recovery reduced La and increased La removal ability |
| Šambaher et al., 2016 | 15 active participants, 8F (22.3 ± 1.5 y) and 7M (24.8 ± 4.32 y) | To examine the effects of compression garments on neuromuscular performance, blood lactate, and skin temperature before and after fatigue | La | Drop jumps from 30cm | During | Graduated compression Ankle: 20 – 30 mmHg | Compression had no benefit on La following drop jumps |
| Scanlan et al., 2008 | 12 well trained cyclists, M, (20.5 ± 3.6 y) | To investigate the effects of wearing lower body compression garments on physiological and performance responses during endurance cycling. | La | 1 hour Time Trial and Incremental Test | During | *Posterior gluteus maximus*: 9.1 ± 2.2 mmHg  *Vastus Lateralis*: 14.9 ± 2.3mmHg Calf: 17.3 ± 3.0 mmHg  Ankle: 19.5 ± 3.4mmHg | Wearing lower body compression garments had no influence on La |
| Sear et al., 2010 | 8 amateur team sport athletes, M, (20.6 ± 1.2 y) | To determine the effects of wearing whole body compression garments on physical and physiological measures during a team sport–specific prolonged high intensity intermittent exercise protocol. | La | 45 min prolonged high intensity intermittent exercise | During | Full Body: Range 5 – 17 mmHg | No effects of compression on La |
| Smale et al., 2017 | 15 well-trained cyclists, M, (28.1 ± 6.3 y) | Examine the effects of varying grades of compression garments during incremental cycling exercise on cerebral artery blood flow velocity and cognitive performance | La | Four 8 min increments of cycling at 30%, 50%, 70%, and 85% maximal power output and a 4 km time-trial. | During | Medium-grade garment: Ankle: 21.8 ± 6.6 mmHg Knee: 20.3 ± 6.6 mmHg Thigh: 15.4 ± 4.5 mmHg  Low grade compression: Ankle: 8.6 ± 2.7 mmHg Knee: 14.9 ± 4.9 mmHg Thigh: 9.1 ± 3.1 mmHg | No effect on La at any time point |
| Sperlich et al., 2010 | 15 healthy runners and triathletes, M, (27.1 ± 4.8 y) | To compare effects of three types of compression clothing (socks, tights, and whole-body) in well-trained athletes on s physiological responses and performance | oxygen saturation, partial pressure of oxygen | Sub-maximal running speed for 15 min. Thereafter, running speed was set at the highest speed achieved during incremental testing. | During | Aimed for 20mmHg | No differences between compression clothing and clothing without external pressure. |
| Sperlich et al., 2013 | 12 elite alpine skiers, M, (26.0 ± 4.0 y) | To evaluate the effects of different levels of compression on the legs of highly trained alpine skiers subjected to passive vibration in the downhill tuck position. | La | 3-min trials in a downhill tuck position involving application of passive vibration to the soles of both feet | During and five minutes after | Moderate compression: Calf: 19.7 ± 3.7 mmHg Thigh: 17.8 ± 1.9 mmHg  High compression: Calf: 39.5 ± 3.5mmHg Thigh: 34.0 ± 2.6mmHg | Compression on the legs of elite alpine skiers performing simulated skiing for 3 min in the tucked position with passive vibration had no effect on La |
| Sperlich et al., 2013 | 10 well-trained endurance athletes, M, (25 ± 4 y) | To assess whether upper body compression garments improve double-poling sprint performance by measuring power output and selected metabolic, cardio-respiratory, hemodynamic and perceptual parameters | La, pH, partial pressure of oxygen, arterial oxygen saturation; | Three 3-min simulated double polling sprints on a cross-country ski ergometer | During | Forearm: 21 ± 5mmHg  m. triceps brachii: 14 ± 3 mmHg  m. biceps brachii: 14 ± 2 mmHg  m. latissimus dorsi: 9 ± 2 mmHg | Upper-body compression during three 3-minute sessions of double polling sprint by well-trained athletes revealed lowering of La, with no changes in pH |
| Struhár et al., 2018 | 10 well trained runners, M, (24.8 ± 3.45 y) | Identify the effect of CGs pressure distribution with graduated low grade compression garments, medium grade compression garments and high reverse grade compression garments on physiological and perceptual measures of performance and recovery | CK | 8 km running on a treadmill with a 6% elevation rate at the intensity of 75% of personal VO_2max_ | During and 4 hours post run | Low CG:  Ankle: 18 mmHg Knee: 15 mmHg  Medium CG: Ankle: 25 mmHg Knee: 21 mmHg  High CG: Ankle: 18 mmHg Knee: 24 mmHg | A beneficial trend in the clearance of CK activity with the medium-grade graduated compression |
| Taylor et al., 2018 | 42 runners, F  Oral contraceptive plus compression Sock, 14F (35.8 ± 6.5 y)  Oral contraceptive group,  15F (36.3 ± 7.5 y)  Control group, 12F (41.8 ± 5.4 y) | Primary aim: Investigate exercise-induced haemostatic activation in women using oral contraceptives, flying cross-country, and running the 2015 Boston Marathon  Secondary aim: to investigate the influence of compression socks on exercise-induced haemostatic activation by enrolling an additional group of oral contraceptive-using female athletes who wore compression socks during outbound and return flights | Thrombin–antithrombin complex; d-dimer; clot breakdown factor tissue plasminogen activase; Hct | 2015 Boston Marathon | Flights before and after marathon | NS | The use of compression socks in women on oral contraceptives resulted in a greater increase in d-dimer after exercise, although with no obvious clinical symptomology |
| Toolis & McGawley et al., 2020 | 7 senior biathletes from the Swedish national team, 3F and 4M, (25.1 ± 3.1y) | To assess the effects of wearing upper- and lower-body compression garments on laboratory-based roller-skiing performance in elite biathletes, using ski durations and techniques simulating the demands of biathlon racing. | La | Roller Ski time trial followed by a test of time to exhaustion | During | Biceps: 7.4 ± 2.2 mmHg  Triceps: 7.9 ± 2.2 mmHg  *Brachioradialis*: 13.1 ± 4.5 mmHg  *Rectus* *femoris*: 13.3 ± 2.3 mmHg  *Gastrocnemius*: 19.9 ± 5.9 mmHg | There were no significant differences in the La between interventions. |
| Turgay Erten et al., 2016 | 20 athletes, 9F volleyball (15.9 ± 0.3y) and 11M basketball (15.27 ± 0.5y) | To assess the effects of compression stockings and electrostimulation on La values and isokinetic strength values during the first 30 min of recovery after 30 min of aerobic running on a treadmill at 85% of heart rate. | La | 30 minutes of treadmill running at 85% of anaerobic threshold | 30 minutes following exercise | Calf: 20 – 30 mmHg | Compression had no benefit on La following 30 min of treadmill running |
| Upton et al., 2017 | 19 club level rugby union players, M, (20.3 ± 1.7 y) | To evaluate the efficacy of compression garments for the recovery of strength, power and indices of muscle damage from a rugby specific, muscle damaging protocol. | CK | Twenty 20m maximal sprints with 10m deceleration. | 48 hours post-exercise | Calf: 14 ± 4.1 mmHg Thigh: 8.5 ± 2.3 mmHg | The compression group had lower CK values than sham, as demonstrated by a significant time by group effect |
| Varela-Sanz et al., 2011 | 16 well-trained runners, 3F (32.00 ± 4.58 y) and 13M (35.41 ± 6.61 y) | To assess the influence of below knee compression stockings on running economy and performance at competitive velocities | La | Experiment 1: (4 x 6min at 1/2 marathon pace)  Experiment 2: running as long as possible on a treadmill at a gradient of 1% and at a speed of 105% of the athlete’s recent 10-km time (average speed of 17 6 2 kmh21) until exhaustion. | During | NS | Wearing gradual elastic compression stockings during a time limit test at competition pace had no impact on La. |
| Vercruyssen et al., 2012 | 11 trained runners, M, (34.7 ± 9.8 y) | To investigate the effects of wearing compression socks on performance indicators and physiological responses during prolonged trail running | La | 15.6 km trail run | During | Calf: 18 mmHg | Competitive runners do not gain any benefits on La from wearing CS during prolonged off-road running |
| Williams et al., 2020 | 10 trained university-level cyclists, M, (21.0 ± 2 y) | To assess the effects of varying levels of compression applied via lower-limb compression garments on multiday cycling performance at typical levels of exercise induced muscle damage associated with multiday exercise events | La, CK, Mb | High intensity protocol, 24h rest, then an 8km time trial | During | Low-pressure compression garment:  distal hem: 7 ± 3 mmHg,  Calf: 7 ± 3 mmHg,  Mid-thigh: 5 ± 2 mmHg,  Head of femur: 5 ± 2 mmHg,  *Posterior superior iliac spine*: 5 ± 1 mmHg  High-pressure compression garment:  Distal hem: 11 ± 3 mmHg,  Calf: 15 ± 3 mmHg,  Mid-thigh: 10 ± 3 mmHg,  Head of femur: 8 ± 2 mmHg,  *Posterior superior iliac spine*: 6 ± 1 mmHg | High level compression reduced La at 30 and 60 min post-exercise.  No differences observed for other blood markers |
| Zadow et al., 2020 | 46 marathon runners  Compression group, 4F and 19M, (45.8 ± 10.0 y)  Control group, 8F and 15M, (41.9 ± 9.9 y) | to determine if wearing lower-body compression socks would reduce intestinal damage associated with running a marathon | intestinal fatty acid–binding protein | Marathon Race | During | Ankle: 25 mm Hg | Wearing compression socks when running a marathon significantly reduces circulating levels of intestinal damage marker intestinal fatty acid–binding protein |
| Zaleski et al., 2015 | 20 Runners  Compression group, 5F and 5M (36.9 ± 8.4 y),  Control group, 5F and 5M (35.5 ± 8.0 y) | To examine the influence of wearing compression socks during a marathon run on CK levels, before, immediately after, and the day following a marathon, among recreational athletes running the 2013 Hartford Marathon | CK, Tissue plasminogen activator; thrombin–antithrombin complex; D-dimer; Hct | Marathon run | During | Ankle: 19 – 25 mmHg | No statistical differences between the compression sock group and control for CK |
| Zinner et al., 2017 | 12 handball players, M, (22 ± 4 y) | To investigate the effects of increasing the level of compression on recovery following repeated sprints. | CK, Urea, CRP | 30 x 30m sprints | 48 hours post-exercise | From below the hip to the foot exerting  mean pressures applied of 3 ± 1 mmHg, 11 ± 1 mmHg, 23 ± 2 mmHg | Plasma concentrations of CK and urea were ‘likely’ to ‘very, very likely’ reduced after 10 mmHg of compression, with no effect of compression on CRP |

M = Male, F = Female, NS = Not specified, CG = Compression garment, CS = Compression sleeve, Hct = haematocrit, Hb = haemoglobin, RM = Repetition maximum, TT = Time trial, PPO = Peak power output, VO_2max_ = Maximal oxygen uptake, VO_2peak_ = Peak oxygen uptake, CK = Creatine kinase, La = Lactate, LDH = Lactate dehydrogenase, IL-6 = Interleukin-6, CRP = C=reactive protein, La = Lactate, Mb = Myoglobin, IL-10 = Interleukin-10, IL1-β = Interleukin 1-β, TNF-α = Tumor necrosis factor-α, EIMD = Exercise induced muscle damage, DOMS = Delayed onset muscle soreness.
