# Supplementary material for: Putting the Squeeze on Compression Garments: Current Evidence and Recommendations for Future Research: A Systematic Scoping Review
Source: Sports Med. 2021 Dec 6;52(5):1141–60. doi: 10.1007/s40279-021-01604-9 (PMC9023423; doi:10.1007/s40279-021-01604-9)
Supplement: Supplementary file 4 — Supplementary file4 (DOCX 72 kb) [file 40279_2021_1604_MOESM4_ESM.docx]

**Supplementary Table S4.** Details of studies and information relevant to cardiovascular and haemodynamic outcomes.

| **Study** | **Cohort/ sample size (n), sex, age** | **Study purpose** | **Outcome Measures** | **Exercise Protocol** | **Compression worn during/after/both** | **Compression pressure – reported value or not stated** | **Key findings** |
| --- | --- | --- | --- | --- | --- | --- | --- |
| Ali et al., 2007 | Experiment 1: 14 healthy participants, M, (22 ± 0.4 y)  Experiment 2: 14 healthy participants, M, (23 ± 0.5 y) | To examine the influence of wearing graduated compression stockings on several physiological and perceptual responses during and after exercise. | Experiment 1: HR  Experiment 2: HR | Experiment 1: two multi-stage intermittent shuttle running tests with 1 h recovery between tests.  Experiment 2: continuous 10-km road run. | During | 18 – 22 mmHg | Compression provided no effect on HR in both experiments |
| Ali et al., 2010 | 10 triathletes, 1F and 9M, (36.0 ± 10.0 y) | To examine the physiological and perceptual responses to wearing graduated compression stockings during fast-paced running | HR | 90% of 10 km personal best speed at 1% incline for 40 mins on treadmill | During | Low CG: 12 - 15 mmHg High CG: 23 - 32 mmHg | Runners experienced no physiological benefits while wearing CG in terms of reduced HR. |
| Ali et al., 2011 | 12 well-trained runners, 3F and 9M, (33 ± 10 y) | Examine the effects of wearing different grades of graduated compression garments on 10-km running performance | HR | 10km running time trial | During | Low condition:  Ankle: 15 mmHg Knee: 12 mmHg  Medium condition: Ankle: 21  Knee: 18mmHg  High condition: Ankle: 32 mmHg  Knee: 23 mmHg | There were no benefits from wearing compression garments on HR following a short distance trail running |
| Armstrong et al., 2015 | 33 moderately trained runners, 10F and 23M, (38.5 ± 7.2 y) | To determine if lower limb compression garments influence functional recovery from distance running | HR | Marathon race | During | Below knee compression socks: Ankle: 30 – 40 mmHg Calf: 21 – 28 mmHg | Wearing below-knee compression socks for 48 hours after marathon running had no effect on HR |
| Barwood et al., 2013 | 8 physically active, M, (21 ± 2 y) | To establish the thermal and performance effects of wearing graduated compression garments in a hot environment in contrast to control and sham treatment conditions | HR. | 15 minutes running at 10-12km/h followed by a 5 km time trial | During | Calf: Compression: 20 ± 3 mmHg Sham, 17 ± 4 mmHg  Thigh: Compression: 11 ± 2 mmHg Sham: 10 ± 2 mmHg | No evidence that wearing either an appropriately fitting (compression) or loose fitting (sham condition) graduated compression garment alters HR |
| Bieuzen et al., 2014 | 11 highly trained runners, M, (34.7 ± 9.8 y) | To examine the effect of wearing compression stockings on indices of EIMD during trail-running. Compression stockings were worn either during or after a trail run performed at competition pace by experienced off-road runners | HRmax | The simulated trail race consisted of 3 laps of 5.2 km (total distance: 15.6 km) in mountainous terrain. | During | Running condition: 25 mmHg Recovery condition: 20 mmHg | No effect on HRmax |
| Book et al. 2016 | 12 healthy, 5F and 7M, (27.3 ± 6.4 y) | To determine how applied external compression to the lower leg influences local and/ or central hemodynamic responses as an indicator of the potential for improved athletic performance when using GCS. | Arterial blood pressure, SV, Q, HR, Peripheral blood velocity, Vessel diameter, Blood pressure, Muscle oxygenation, tHb, blood volume | 5 min plantar flexion exercise | During and 5 min after | Pressure difference from ankle to knee ~ 10mmHg | The use of compression had no effect on central hemodynamics, or popliteal arterty blood velocity.  However, blood volume distribution was altered with compression. |
| Born et al., 2014 | Sub-study 1:  12 track and team sport athletes, F, (25.0 ± 3.0 y)  Sub-study 2: 12 track and team sport athletes, F,  (23 ± 2 y) | There were 2 aims; 1) To assess the effects of compression garments with silicone stripes (which mimic kinesio taping) on repeated sprint performance; 2) to identify the physiological, biomechanical, and perceptual effects of compression garments with silicone stripes | HR, tissue saturation index, HbO_2_, HHb, tHb | 30 x 30-m sprints (one sprint per minute) | During | ~18 – 20 mmHg across the entire lower body | There were no significant differences in physiological measures between conditions |
| Born et al., 2014 | 10 elite German ice speed skaters, M, (23 ± 7 y) | To investigate whether the application of compression improves muscle oxygenation and blood volume, ratings of perceived exertion; blood lactate concentration; and, 3000-m time. | HR, Muscle oxygenation, oxygen uptake, HbO_2_, HHb, tHb | 3000-m race simulation | During | Thigh: 20.3 ± 2.3 mmHg  Calf: 24.4 ± 3.1 mmHg | Compression provided no effect on selected cardiorespiratory and circulatory parameters during 3000-m ice speed skating. |
| Boucourt et al. 2015 | 11 healthy athletes, Sex NS, (29.6 ± 2.8 y) | To assess the changes in tissue oxygen saturation with calf compression sleeves before, during, and after an incremental cycling exercise | Tissue oxygen saturation | 15 min of cycling, with three minutes at 40, 80, 120, 160, 200w | During and after | Ankle: 14mmHg Calf: 28mmHg | Calf compression sleeves increased tissue oxygen saturation at rest (before and after exercise) and at low intensities during cycling |
| Bringard et al., 2006 | Test 1: 6 trained runners, M, (31.2 ± 5.4 y)  Test 2: 6 trained runners, M, (26.7 ± 2.9 y). | Experiment 1: To examine the effect of wearing compression tights compared to wearing shorts and classic tights on aerobic energy cost of running at various submaximal running intensities.  Experiment 2: evaluate the effects of wearing compression tights on the excess in VO2 during prolonged submaximal exercise. | Exp 1: HR | Exp 1: continuous incremental exercise test to voluntary exhaustion  Exp 2: 15 min run at 80% VO_2max_ | During | NS | Compression had no effect on HR |
| Broatch et al., 2017 | 20 recreationally active participants, 11F (25.0 ± 2.0 y) and 9M (28.0 ± 6 y) | This study aims to assess the effects of lower-limb compression garments on markers of blood flow, oxygen kinetics, and exercise performance during a repeated sprint protocol with short rest intervals | Muscle blood flow, mVO_2_, HR | 4 sets of 10 x 6-s maximal sprints, inter-spaced by 24 s of recovery between bouts and 2 min recovery between sets | During | Thigh: 11.7 ± 2.3 mmHg Calf: 26.4 ± 6.4 mmHg Ankle: 21.5 ± 8.2 mmHg | Lower-limb compression garments worn during repeated sprint exercise improved muscle blood flow and HR measures |
| Broatch et al., 2019 | 12 elite Australian volleyball athletes, F, (25 ± 2 y) | To determine the effects of wearing compression socks during long-haul travel on sports-specific performance, physiological, and haematological alterations in elite female volleyball athletes | Systolic BP, HR, oxygen saturation | Air travel | Worn during travel | Max calf girth: 23 ± 11 mmHg  Ankle: Between 19 and 22 (±8) mmHg | Compression enhanced measures of systolic BP, HR and oxygen saturation |
| Chatard et al., 2004 | 12 trained cyclists, M, (63 ± 3 y) | To determine whether compression stockings affect performance recovery and leg pain following maximal exercise. | HR | All out five-minute cycloergometer followed by 80-min rest and then another all out five minutes | During | Ankle: 44  Calf: 24 hPa  Mid-thigh:17 hPa which represented a 40% pressure decrease between the ankle and the mid-thigh | There were no benefits from wearing compression garments on HR |
| Coza et al., 2012 | 16 physically active participants, M, (26.3 ± 5.1 y) | To to quantify the effects compression on the gastrocnemius medialis muscle energy use during short-term activity and gastrocnemius medialis tissue oxygenation at the beginning of exercise. | Tissue oxygen index, HbO_2_, HHb, tHb, oxymyoglobin, dexoymyoglobin, tissue oxygenation depletion rate. | 40 heel raises per min for 2 min | During | NS | Externally applied compression does not have a significant impact on physiological measures during a dynamic task. |
| Dascombe et al., 2011 | 11 well-trained middle-distance runners and triathletes, M, (28.4 ± 10.0 y) | To determine the effectiveness of wearing an undersized lower body compression garment on physiological and performance parameters relating to endurance running. | HR, HbO_2_, HHb, Tissue Oxygenation Index, tHb | Time to exhaustion test consisting of the participant running at 90% of VO_2max_ velocity until volitional exhaustion | During | Regular size:  Thigh: 13.7 ± 2.3 mmHg Calf: 19.2 ± 3.2 mmHg  Undersize:  Thigh: 15.9 ± 2.6 mmHg Calf: 21.7 ± 4.3 mmHg | At slower running velocities (8-10 kmh) CG conditions significantly increased muscle blood flow and O2 utilization. During the faster running velocities (>12 kmh), CG conditions significantly increased the HHb concentration within the vastus lateralis, which coincided with a decrease in HR and tissue oxygen index. |
| Dascombe et al., 2013 | 7 elite flat-water kayakers, 2F (25.0 ± 4.2 y) and 5M (21.8 ± 2.8 y) | To determine the effects of wearing upper body compression garments on performance during simulated flat-water kayaking and physiological responses during simulated kayaking | blood flow, HbO_2_, HHb, tHb Tissue Saturation Index, HR. | Participants completed a six-step incremental test and a subsequent 4min performance test on a kayak ergometer | During | NS | No significant improvements in the selected physiological measures during simulated flatwater kayaking in elite kayakers wearing the upper body CG. |
| Dorey et al. 2018 | 10 healthy participants. 4F and 6M, (23.0 ± 2.0 y) | To test that knee‐high compression socks are effective at minimizing post‐exercise reductions in stroke volume, cardiac output, and cerebral blood flow velocity during a 15‐minute passive 60° head up tilt in a group of young, healthy, normotensive adults. | CBV, Q, HR, MAP, Total vascular conductance | 60 min of moderate-intensity (60% VO_2peak_) cycling exercise | During | Compression socks: 30 ‐ 40 mmHg | Compression socks attenuated the reductions in SV, Q, CBV, as well as, minimized the increase in HR. |
| Driller and Halson 2013 | 12 highly trained male cyclists, M, (30 ± 6 y) | To investigate the effect of wearing lower body compression garments on performance during a 30-minute endurance cycling test. A further aim of the study was to determine various physiological and perceptual responses when wearing compression garments during the cycle test. | HR | 15 minutes at a workload equal to 70% PPO, followed immediately by a 15-minute time trial. | During | ~18 mmHg at the medial malleolus decreasing to ~10 mmHg at the gluteus maximus | There were no significant differences in measures between conditions. |
| Duffield and Portus, 2007 | 10 club cricket players, M, (22.1 ± 1.1 y) | To compare the effects of three different types of full-body compression garments and a control condition on performance in intermittent, repeat-sprint and throwing performance in cricket players. | HR | 30 min repeat-sprint exercise protocol comprising 20 m sprints every minute, separated by submaximal exercise. Throwing tests included a pre-exercise and a post-exercise maximal distance test and accuracy throwing tests. | During and 24 hours after | NS | No effect of CG on HR |
| Duffield et al., 2010 | 11 rugby players, M, (20.9 ± 2.7 y) | Examine the effect of wearing compression garments during and 24 h following high-intensity, intermittent-sprint and stretch shortening cycle activities on post-exercise performance and recovery of evoked and voluntary muscle performance. | HR | 10-min exercise protocol of a 20-m sprint and 10 plyometric bounds every min | During and for 24 hours after | NS | No effect of CG on HR |
| Duffield et al., 2014 | 8 professional tennis players, M, (20.9 ± 3.6 y) | To investigate the effects of combining cold water immersion, compression garments, and sleep-hygiene recommendations on physical, physiological, and perceptual recovery after 2-a-day on-court training and match-play sessions | HR | Each respective on-court session involved 90 minutes of coach-led drills (including a 30-min warm-up) and 90 minutes of competitive match play | Between drill and match play (~3 hours) and for 4 hours match play | NS | Post-session cold water immersion and compression garments exhibited large positive effects for HR |
| Ehrstrom et al., 2018 | 13 trail runners, M, (38.6 ± 5.7 y) | To examine whether wearing high-pressure compression garments during a 40-min treadmill downhill run on acute and delayed neuromuscular responses and running economy | HR | 40-min downhill running at –8.5 deg decline | During | 15–20 mmHg for quadriceps and calves | The use of high-pressure compression garments during downhill running had no effect on HR response |
| Faulkner et al., 2013 | 11 trained runners, M (23.7 ± 5.7 y) | To examine the effects of lower-limb compression on 400-m run performance and physiological and perceptual indicators of intensity and performance | HR | 400m sprint | During | Long garment: 2.0 - 13.2 mmHg  Short length garment: 3.7-20.7 mmHg | There were no significant differences in HR between conditions. |
| Fujii et al., 2017 | Nine, M, (24.7 ± 2.0 y) | To examine whether stocking-mediated graduated compression augments cutaneous vasodilation but not sweating during exercise in the heat. | HR, MAP and forearm blood flow | Participants performed cycling at 60% peak oxygen uptake at a pedalling rate of 60rpm for 45 min or until body core temperature reached ~1.5°C above baseline resting values | During | Ankle: 26.4 ± 5.3 mmHg  Calf: 17.5 ± 4.4 mmHg  Thigh: 6.1 ± 2.0 mmHg | Graduated compression induced increases in cutaneous vasodilation during exercise in the heat, but no effect on HR or MAP |
| Gimenes et al., 2019 | 20 football players, Sex NS, 10 compression (18.3 ± 0.5 y) and 10 control (18.5 ± 0.5 y) | the effects of using compression stockings on the match-based physical performance indicators, HR responses, and perceptual measurements in under-20 soccer players during 2 matches separated by 72 hours | HR mean and peak | Two football matches separated by 72 hours | During | Estimated between 20-30 mmHg | No difference in HR outcomes between conditions |
| Glanville and Hamlin 2012 | 14 trained multisport, M, (33.8 ± 6.8 y) | To determine the effects of wearing commercially available graduated compression garments during prolonged recovery (24hours) on subsequent 40-km cycling time trial performance in trained multisport athletes. | HR and BP | 40-km cycling performance | Post-exercise for 24 hours | Upper ankle: 6.0 ± 2.4 mmHg Upper calf: 14.7 ± 2.5 mmHg Upper leg segment: 11.8 ± 2.5 mmHg | Compression provided no effect on physiological measures post-exercise |
| Hamlin et al., 2012 | 22 rugby union players, M, (20.1 ± 2.1 y) | To determine the effects of wearing either a compression garment or placebo garment over a 24-hour recovery period on subsequent physiological and performance measures | HRmean and HRmax | A series of exercise circuits designed to simulate a game of rugby | After | Compression:  *Sphyrion*: 8.6 ± 2.6 mmHg Mid-calf: 13.4 ± 2.0 mmHg *Mid-trochanterion*: and 9.0 ± 2.2 mmHg  Control:  *Sphyrion*: 2.6 ± 1.2 mmHg, Mid-calf: 5.0 ± 1.5 mmHg *Mid-trochanterion*: 3.5 ± 0.9 mmHg | Wearing a compression garment for 24 hours after high-intensity exercise had no influence on HR measures |
| Higgins et al., 2009 | 9 Netballers, F, (22.6 ± 4.6 y) | the purpose of this study was to examine effectiveness of compression garments on physiological and performance markers in a game-specific circuit for netball. | HR | A circuit designed to simulate competitive netball. 4 x 15 min circuit per session to simulate the four quarters of a competitive game. The circuit comprised of six stations simulating game conditions that were repeated throughout each quarter. | During | NS | Compression provided no effect on HR during exercise |
| Hintzy et al., Ahead of print | 12 participants, M, (25.3 ± 3.6 y) | Examine the effects of different levels of thigh compression in shorts on both vibration and muscle activity of the thigh during cycling with superimposed vibrations. | HR | Four sets of 18-minute cycling test | During | 0, 2, 6 and 15-mmHg for depending on condition. | There were no benefits from wearing compression garments on HR |
| Houghton et al., 2007 | 12 trained amateur field hockey players,  M, (21[19-23] y) | To investigate the effects of compression garments on thermoregulation in field hockey players. | HR | The Loughborough intermittent shuttle test | During | NS | Similar HR between trials. |
| Hu et al. 2020 | 10 novice runners, M, (21.5 ± 1.4 y) | To determine if novice runners experienced augmented recovery by wearing lower limb graded compression garment (GCG) following a running program designed to induce overtraining. | HRV | 2 week exercise (2 miles and increased by 0.5 miles every other day) | 4-5 h post exercise | Ankle: 26 mmHg  Glute: 8 mmHg | Compression may counteract some deleterious effects from overtraining (absent of adequate rest/recovery) while attenuating its effects on vagally-mediated HRV |
| Kemmler et al., 2009 | 21 moderately trained runners, M, (39.3 ± 10.7 y) | To determine the effect of below-knee stockings with constant compression on selected parameters of running performance in healthy male runners | HR | Stepwise-speed incremental running test | During | Ankle: ~24mmHg Calf: ~18-20mmHg | Compression provided no effect on physiological measures during exercise |
| Kerhervé et al., 2017 | 14 participants, M, (21.7 ± 3.0 y) | To determine if wearing calf compression sleeves during a prolonged running exercise affected local muscle tissue oxygenation, running pattern, muscle power capability, performance, and subjective perception of muscle fatigue, pain and soreness. | muscle oxygenation, HR, Muscle blood flow and mVO_2_ | 24 km run | During | 23 ± 2 mmHg | Compression sleeves improved muscle oxygenation and muscle blood flow |
| Leicht et al., 2020 | 30 active participants, 15M and 15F, (19.8 ± 1.9 y) | To examine the impact of lower body compression garments on cardiac autonomic control of heart rate prior to, during and following submaximal exercise. | HR and HRV | Cycling at a moderate intensity equivalent to 70% age-predicted maximum heart rate | During and after | NS | CG had minimal impact on physiological responses during and following submaximal exercise in healthy adults. |
| Leoz-Abaurrea et al 2015 | 13 untrained participants, M, (21 ± 6 y) | To investigate whether a heat dissipating upper body compression garment can mitigate thermoregulatory strain better than non-compression garments during cycling in hot (i.e., 40 degree) temperatures. | HR | Cycling at a fixed workload (~50% VO_2peak_) with 4 bouts of 14 minutes at 40 ºC with each bout separated with a minute active recovery | During | NS | Upper body compression garment impaired HR responses during recovery. |
| Leoz-Abaurrea et al 2016 | 10 recreational runners, M, (23.0 ± 3.0 y) | To analyse the physiological responses of heat dissipating upper body compression garments during a running performance test to exhaustion | HR | 45-min run at 60% of the peak treadmill speed followed by a time to exhaustion run at 80% of the peak treadmill speed | During | *Biceps brachii*: 2.9 ± 1.5 mmHg Triceps: 3.0 ± 1.0 mmHg *Pectoralis major*: 2.0 ± 0.5 mmHg *Latissimus dorsi*: 1.4 ± 0.5 mmHg | Upper body compression garment impaired HR responses during exercise. |
| Leoz-Abaurrea et al 2016 | 16 untrained participants, 4F and 12M, (21.3 ± 5.7 y) | To determine the effects of upper body compression garments on thermoregulatory responses during cycling in a controlled laboratory thermoneutral environment (~23°C). A secondary aim was to determine the cardiovascular and perceptual responses when wearing the garment. | HR | Cycling at a fixed workload (~50% VO_2peak_) with four bouts of 14 minutes at 40 ºC with each bout separated with a minute active recovery | During | NS | Compression had no effect on HR |
| Leoz-Abaurrea et al., 2017 | 12 trained individuals, M, (66.0 ± 2.0 y) | To evaluate the effects of an upper body compression garment vs. a control garment on thermoregulatory responses in trained older adults in a temperate environment. | HR and MAP | Cycling trial consisted of 4 bouts at a fixed load (50% peak power output) for 14 min, with each separated with a minute rest | During | Arm: ~1-3 mmHg of compression | Results showed that wearing an upper body CG had no effect on HR or MAP |
| Leoz-Abaurrea et al 2017 | 20 recreational road cyclists, 4F and 16M, Compression group (21.4 ± 4.4 y), Control group (19.9 ± 2.5 y) | To evaluate the effects of a heat dissipating upper body compression garment on thermoregulatory, cardiovascular, and perceptual responses during continuous cycling at a moderate intensity in a hot environment. | HR | Cycling trial consisted of 30 minutes at a moderate intensity (~50% V̇O_2max_). | During | NS | The group of participants who wore the UBCG finished the 30-minute exercise bout at 40ºC with significantly greater cardiovascular strain |
| Lovell et al., 2011 | 26 semi-professional rugby league players, M, (21.6 ± 2.5 y) | To examine the effect of compression garments on active post-exercise recovery after a bout of high-intensity exercise | HR | A 6-stage submaximal treadmill test which consisted of 5-minute stages at 6 km/h, 10 km/h, approximately 85% of VO_2max_, and 6 km/h as a recovery stage followed by approximately 85% of VO_2max_ and 6 km/h | During | Ankle: 20 ± 2 mmHg Calf: 15 ± 2 mmHg | HR was lower when wearing compression garments during active recovery after a bout of high-intensity running |
| MacRae et al., 2012 | 12 recreationally trained cyclists, M, (26 ± 7 y) | To examine the effects of full-body compression garments on cardiovascular and thermoregulatory function during rest and exercise, and on exercise performance | Q, SV, TPR, left ventricle rejection time, BP, vasodilation response and HR | 60-min fixed-load cycling at ~65% VO_2max_ and a 6-km time trial. | During | Correctly-sized group: 11 – 15 mmHg Over-sized group: 8 – 13 mmHg | Full-body CG did not significantly augment SV during rest or prolonged dynamic exercise. These garments caused increases in skin temperature and exercising cardiac output without affecting core temperature, SV, arterial blood pressure |
| Marqués-Jiménez et al., 2017 | 18 semi-professional football players, M, (25.2 ± 3.0 y) | To evaluate the influence of wearing different types of compression garments during matches and recovery after a friendly soccer match | HR | Soccer match | Each participant in the experimental condition played the match wearing one type of graduated compression garment and kept wearing them 7 hours per day for 3 days post-match | Compression stockings: Ankle: 20 – 25 mmHg  Calf: 15–20 mmHg  Compression tights:  Calf: 25–30 mmHg  Thigh: 15–20 mmHg  Compression shorts: Thigh: 15–20 mmHg | Compression garments did not alter HR responses when worn during and post a soccer match. |
| Ménétrier et al., 2011 | 14 moderately trained endurance participants, M, (21.9 ± 0.7 y) | To test whether calf compression sleeves increase calf oxygen saturation and running performance. | Calf oxygen saturation, HR | 30 min at 60 % maximal aerobic velocity, 15 min of recovery, a running time to exhaustion at 100 % maximal aerobic velocity | Before, during and after | Ankle: 15 mmHg Calf: 27 mmHg | Compression sleeves increased calf oxygen saturation at rest and during recovery from exercise.  No change in HR |
| Ménétrier et al., 2015 | 15 endurance trained, M, (22.5 ± 0.7 y) | To assess the effect of compression stockings and contrast water therapy on muscle leg blood flow following a high-intensity interval training session. | Femoral diameter and artery cross-sectional area, Time-averaged mean velocity, peak systolic velocity and end diastolic velocity, HR, resistance index. | Cycling, 9 x 5 min intervals (4 min at 50% of peak power output followed by 1 min at 80% of PPO). | After | Thigh: 14 mmHg Calf: 27 mmHg Ankle: 15 mmHg | During immediate recovery of a high intensity exercise, compression garments result in higher femoral artery blood flow than passive recovery. |
| Mizuno et al., 2017 | 30 physically active participants  Compression thigh group, 10M,  (21.3 ± 0.4 y)    Compression sock group 10M, (21.6 ± 0.8 y)  Control group, 10M, (22.9 ± 0.7 y) | Examine the effects of the body coverage area of compression garments on the exercise performances and muscle damage during prolonged running | HR | 120min of uphill running at 55% of VO_2max_ | During | Thigh compression group:  14.7 ± 0.6 mmHg  Calf compression group: 17.4 ± 0.5 mmHg  Control group: Thigh: 3.0 ± 0.3 mmHg Calf: 1.8 ± 0.2 mmHg | The present findings revealed no significant effects of the body coverage area of the compression garments on HR |
| Mizuno et al., 2017 | 8 participants, M, (23.4 ± 2.4 y) | To investigate the effect of wearing lower body compression garments exerting different pressure levels during prolonged running on exercise-induced muscle damage and the inflammatory response. | HR | 120 min of uphill running at 60% of VO_2max_ | During | High pressure garment: Thigh: 26.9 ± 3.3 mmHg Calf: 29.2 ± 3.8 mmHg  Medium pressure garment: Thigh: 16.1 ± 2.0 mmHg Calf: 17.9 ± 3.5 mmHg  Control garment:  Thigh: 4.4 ± 1.2 mmHg Calf: 3.0 ± 1.6 mmHg | The medium pressure showed a significantly smaller increase in HR compared with that in the control trial |
| Neves Pavin et al. 2018 | 20 amateur soccer players, F, (20.6 ± 3.9 y) | To evaluate the effect of compression stocking use during an amateur female soccer match on match-induced fatigue indicators | HR | Soccer match | During | NS | Wearing compression garments during a soccer match did not alter HR.  . |
| Nguyen et al. 2019 | Study 1: 8 healthy, 3F and 5M, (25.1 ± 3.8 y)  Study 2: 14 healthy, 7F and 7M, (24.7 ± 4.5 y) | To investigate the underlying mechanism of cardiovascular effects associated with compression garments through exercise and recovery based on ECG signals | HR, QTcorrected intervals, ST intervals | 10-min running on 0% grade at 6 km/h of a treadmill. Following every 2 min of running, the treadmill was speeded up by 1 km/h. The tests remained until the velocity of 11 km/h was finished | During and 2 hours post-exercise | NS | CG showed a positive influence in non-athletes based on the quicker recovery in HR, ST, and QTcorrected. |
| Oficial-Casado et al. 2020 | 10 runners, M, (35.0 ± 5.0 y) | To (1) quantify venous return after submaximal running effort using sports compression socks, and (2) analyse how venous return flow is influenced by medium or high compression socks compression. | Popliteal vein mean blood flow | 30 min at 75% of their maximum aerobic speed | During | NS | The type of compression applied by the compression socks does not influence the volume of venous popliteal vein blood return flow from the legs before or after running. |
| Okamoto et al., 2012 | 10 healthy participants, M (29.8 ± 5.9 y) | Investigate the acute effect of brisk walking with and without graduated compression garments on vascular endothelial function and oxidative stress | HR. brachial artery diameter. Brachial artery flow-mediated dilation. Brachial blood velocity. Hyperaemic response. Brachial vascular conductance. Shear rate. BP | Walking on a motor-driven treadmill at 5.9–6.7 km/h for 30 min at an intensity of ~60% of heart rate reserve | During | Ankle: 25mmHg  Calf: 17mmHg | GCSs suppresses the decrease in brachial artery flow mediated dilation.  No effect on HR |
| Piras and Gitta 2017 | 10 athletes, M, (21.6 ± 1.6 y) | To investigate the effects of a whole-body compression garment after a swimming performance on hemodynamic parameters and on autonomic nervous system activity on subsequent 90 minutes of recovery | BP, HRV, baroreflex sensitivity, SV, Q, TPR and HR | 400m freestyle | For 90 minutes following exercise | Forearm: ∼13 mmHg  Upper arm: ∼10 mmHg Chest: ∼6 mmHg Calf ∼15 mmHg Thigh: ∼8 mmHg Hip: ∼5 mmHg | Compression garments had an effect on the pattern of autonomic function recovery and hemodynamic variables. |
| Priego et al., 2015 | 20 recreational runners,  7F and 13M,  (28.1 ± 5.4 y) | To analyse the effects of running with and without GCS for three weeks on cardiorespiratory parameters in runners | HR | Running test: 30 minutes at 80% of maximal aerobic speed | During | Ankle: 24mmHg  Calf: 21mmHg | No effect on HR was observed after three weeks of running with GCS. |
| Priego Quesada et al., 2015 | 44 runners, 29 M and 15 F, (29.3 ± 5.8 y) | To analyse the effects of running in a moderate environment with and without GCS on skin temperature in runners. | HR | 20min at 75% maximal aerobic speed | During | Ankle: 20–25 mmHg Knee: 15–10 mmHg | Running with graduated compression stockings did not modify HR |
| Pruscino et al., 2013 | 8 highly trained hockey players, M, (21.9 ± 2.3 y) | To investigate the efficacy of wearing a full-length, lower-body compression garment following a hockey-simulation and post-exercise biochemical response and recovery of muscle function | HR | Hockey simulation protocol | 24 hours after | Ankle:19.1 mmHg Calf: 7.2 mmHg Thigh: 4.9 mmHg | CG did not influence HR |
| Rennerfelt et al. 2019 | 20 runners, 10F and 10M, (27.0 [22‒35] y) | To examine the effect of wearing exercise compression stockings on the anterior compartment pressure, oxygenation of the tibialis anterior muscle, and early blood biomarkers change for muscle damage during a 10-km treadmill run in healthy subjects | Tissue oxygenation index and BP | 10-km treadmill run | During | Ankle: 25mmHg | Wearing exercise compression stockings during and following a 10-km treadmill run reduced muscle tissue oxygenation in the anterior compartment of healthy runners |
| Rider et al., 2014 | 10 runners,  3F (18.7 ± 0.6 y) and 7M (21 ± 1.3 y) | To determine whether wearing below-the-knee graduated compression stockings e during a maximal treadmill run would induce physiological changes among collegiate cross-country runners. | HR | 5-km running time trial | During | Ankle: 20 mmHg Calf: 15 mmHg | Compression provided no effect on physiological measures |
| Rimaud et al., 2010 | 8 endurance trained participants, M, (27.1 ± 0.9 y) | To investigate effects of wearing compression stockings during exercise | ECG, HR and BP | Volitional test to exhaustion | During and 60 min after | Maximum of 22mmHg at the calf and 12mmHg at the ankle | Compression had no influence physiological measures during and post-exercise |
| Rivas et al., 2016 | 13 endurance trained runners,  3F and 10M, (20.9 ± 2.5 y) | To determine if commercially available below the knee lower leg compressions would improve resting/submaximal/maximal exercise test cardiorespiratory measurements (heart rate, breathing rate, ventilation, oxygen uptake), lactate metabolism, and perception of exercise intensity during running in endurance-trained athletes | HR | The incremental graded exercise test consisted of baseline rest and submaximal intensities at 23%, 70%, 75%, 85% and then a progressive increase to 100% VO_2max_ | During | Ankle: 12 – 15 mmHg, Calf: 9 – 12 mmHg | Compression provided no effect on HR |
| Rugg and Sternlicht 2013 | 14 healthy participants, M, (28.2 ± 14.0 y) | To investigate if wearing graduated compression tights, compared with loose fitting running shorts, improves and sustain countermovement jump height after submaximal endurance running | HR. | 15 minutes of continuous running with 5 minutes performed at each of the following intensities: 50%, 70%, and 85% of heart rate reserve | During | Ankle: 18.0 mmHg Calf: 12.6 mmHg Thigh: 7.2 mmHg | There were no significant differences in HR between conditions. |
| Scanlan et al., 2008 | 12 well trained cyclists, M, (20.5 ± 3.6 y) | To investigate the effects of wearing lower body compression garments on physiological and performance responses during endurance cycling. | HR, Muscle oxygenation and muscle oxygenation economy | 1 hour Time Trial and Incremental Test | During | *Posterior gluteus maximus*: 9.1 ± 2.2 mmHg *Vastus Lateralis*: 14.9 ± 2.3 mmHg Calf: 17.3 ± 3.0 mmHg Ankle: 19.5 ± 3.4 mmHg | Compression provided no effect on physiological measures during exercise |
| Sear et al., 2010 | 8 amateur team sport athletes, M, (20.6 ± 1.2 y) | To determine the effects of wearing whole body compression garments on physical and physiological measures during a team sport–specific prolonged high intensity intermittent exercise protocol. | HR, HbO_2_, HHb and tHb  tissue oxygenation index | 45 min prolonged high intensity intermittent exercise | During | Full Body: 5 – 17 mmHg | Likely increase in both the average and post-sprint tissue oxygenation index in the whole body CG condition |
| Smale et al., 2017 | 15 well-trained cyclists, M, (28.1 ± 6.3 y) | Examine the effects of varying grades of compression garments during incremental cycling exercise on cerebral artery blood flow velocity and cognitive performance | CBV, MAP and HR | Four 8 min increments of cycling at 30%, 50%, 70%, and 85% maximal power output and a 4 km time-trial. | During | Medium-grade garment: Ankle: 21.8 ± 6.6 mmHg  Knee: 20.3 ± 6.6 mmHG Thigh: 15.4 ± 4.5 mmHG  Low grade compression: Ankle: 8.6 ± 2.7 mmHg Knee: 14.9 ± 4.9 mmHg Thigh: 9.1 ± 3.1 mmHg | Compression provided no effect on selected circulatory parameters. |
| Sperlich et al. 2013 | 6 physically active, M, (22.0 ± 2.0 y) | To 1) study skeletal muscle blood flow and glucose uptake during recovery from high intensity exercise, and 2) investigate whether compression clothing enhances skeletal muscle blood flow and glucose uptake. | Muscle blood flow | Ramp cycling test | After | Thigh: 36.7 ± 4.1 mmHg | When applying 37 mmHg external compression to the thigh muscle, blood flow during recovery is decreased |
| Sperlich et al., 2013 | 10 well-trained endurance athletes, M, (25 ± 4 y) | To assess whether upper body compression garments improve double-poling sprint performance by measuring power output and selected metabolic, cardio-respiratory, hemodynamic and perceptual parameters | HR, SV, Q, tissue saturation, HbO_2_, HHb, tHb, | Three 3-min simulated double polling sprints on a cross-country ski ergometer | During | Forearm: 21 ± 5mmHg  m. triceps brachii: 14 ± 3 mmHg  m. biceps brachii: 14 ± 2 mmHg  m. latissimus dorsi: 9 ± 2 mmHg | Upper-body compression revealed no influence on hemodynamic responses examined. |
| Sperlich et al., 2013 | 12 elite alpine skiers, M, (26.0 ± 4.0 y) | To evaluate the effects of different levels of compression on the legs of highly trained alpine skiers subjected to passive vibration in the downhill tuck position. | HbO_2_, HHb, tHb, Tissue Saturation Index and HR | 3-min trials in a downhill tuck position involving application of passive vibration to the soles of both feet | During and five minutes after | Moderate compression: Calf: 19.7 ± 3.7 mmHg Thigh: 17.8 ± 1.9mmHg  High compression: Calf: 39.5 ± 3.5 mmHg Thigh: 34.0 ± 2.6 mmHg | Greater deoxygenation of the vastus lateralis but no differences in whole-body oxygen consumption or other physiological measures with CG. |
| Toolis and McGawley et al., 2020 | 7 senior biathletes from the Swedish national team, 3F and 4M (25.1 ± 3.1 y) | To assess the effects of wearing upper- and lower-body compression garments on laboratory-based roller-skiing performance in elite biathletes, using ski durations and techniques simulating the demands of biathlon racing. | HR | Roller Ski time trial followed by a test of time to exhaustion | During | Biceps: 7.4 ± 2.2 mmHg Triceps: 7.9 ± 2.2 mmHg *Brachioradialis*: 13.1 ± 4.5 mmHg Rectus femoris: 13.3 ± 2.3 mmHg Gastrocnemius: 19.9 ± 5.9 mmHg | Compression was shown to elicit a moderate effect of a lowered HR at rest, post warm-up, and post time trial |
| Treseler et al., 2016 | 19 physically active participants,  F, (20 ± 1 y) | To examine the physiological and perceptual responses to wearing below-the-knee compression stockings after a 5-km running test in recreationally active women | HR | 5km time trial | During | Ankle: 18 – 21 mmHg Below the knees: 12.6 –14.7 mmHg | Compression stockings did not significantly influence HR. |
| Varela-Sanz et al., 2011 | 16 well-trained runners, 3F (32.00 ± 4.58 y) and 13M (35.41 ± 6.61 y) | To assess the influence of below knee compression stockings on running economy and performance at competitive velocities | Experiment 1: HR  Experiment 2: HR | Experiment 1: (4 x 6min at 1/2 marathon pace)  Experiment 2: running as long as possible on a treadmill at a gradient of 1% and at a speed of 105% of the athlete’s recent 10-km time (average speed of 17 6 2 kmh21) until exhaustion. | During | NS | Percentage of HRmax reduced during a time limit test at competition pace with CG |
| Venckunas et al., 2014 | 13 active participants, F, (25.1 ± 4.2 y) | To evaluate the effect of lower body compression garments on the cardiovascular function in response to a running session in a thermoneutral environment | HR, BP, tissue oxygenation, arterial blood flow, peak venous emptying rate and venous reserve volume. | 4 km was covered in 30 min and 400m sprint | During | Thigh: ~17 mmHg Upper calf: ~19 mmHg | Venous emptying rate was enhanced with CG at 30 min post exercise. No other differences were found both between conditions |
| Vercruyssen et al., 2012 | 11 trained runners, M, (34.7 ± 9.8 y) | To investigate the effects of wearing compression socks on performance indicators and physiological responses during prolonged trail running | Oxygenation and HR | 15.6 km trail run | During | 18 mmHg applied to the calf | No benefit from wearing CS on oxygenation or HR measures during prolonged off-road running |
| Vercruyssen et al., 2016 | 12 runners, M, (39.6 ± 4.6 y) | To examine the influence of wearing compression garment vs. conventional running clothing on muscle contractile function and running economy before and after a short distance trail running. | HR | 18.4-km short distance trail runs | During | Ankle: 18 mmHg Calf: 13 mmHg Short-tight: 7.5 mmHg | There were no benefits from wearing compression garments on HR following short distance trail running. |
| Webb and Willems, 2010 | 18 non-physically active, M, (20 ± 1 y) | Examine the effect of wearing lower body compression garments during downhill running on recovery of jump height and delayed onset muscle soreness. | HR | 5 x 8 min bouts of downhill running performed at 80% of VO_2max_ at a -10% gradient. 2 min static recoveries followed each bout | During exercise | Calf: 18 mmHg Thigh: 9 mmHg | Results indicate that CG demonstrated no benefit on HR |
| Williams et al., 2020 | 10 trained university-level cyclists, M, (21.0 ± 2 y) | To assess the effects of varying levels of compression applied via lower-limb compression garments on multiday cycling performance at typical levels of exercise induced muscle damage associated with multiday exercise events | HR | High intensity protocol, 24h rest, then an 8km time trial | During | Low-pressure compression garment:  *distal hem*: 7 ± 3 mmHg Calf: 7 ± 3 mmHg Mid-thigh: 5 ± 2 mmHg *Head of femur*: 5 ± 2 mmHg *Posterior superior iliac spine*: 5 ± 1 mmHg  High-pressure compression garment: Distal hem: 11 ± 3 mmHg Calf: 15 ± 3 mmHg Mid-thigh: 10 ± 3 mmHg *Head of femur*: 8 ± 2 mmHg *Posterior superior iliac spine*: 6 ± 1 mmHg | No benefit of CG on HR |

M = Male, F = Female, NS = Not specified, CG = Compression garments, CS = Compression sleeve, GCS = Graduated compression stocking, HR = Heart rate, HRV = Heart rate variability, HRmean = Mean heart rate, HRmax = Max heart rate, PPO = Peak power output, VO_2max_ = Maximal oxygen uptake, VO_2peak_ = Peak oxygen uptake, ECG = Electrocardiogram, BP = Blood pressure , Q = Cardiac output, SV = Stroke volume, TPR = Total peripheral resistance, HbO_2_ = Oxyhaemoglobin, HHb = Deoxyhaemoglobin, tHb = Total haemoglobin, MAP = mean arterial pressure, EIMD = Exercise induced muscle damage, CBV = Cerebral blood flow velocity, mVO_2_ = Muscle oxygen consumption
