# Supplementary material for: Putting the Squeeze on Compression Garments: Current Evidence and Recommendations for Future Research: A Systematic Scoping Review
Source: Sports Med. 2021 Dec 6;52(5):1141–60. doi: 10.1007/s40279-021-01604-9 (PMC9023423; doi:10.1007/s40279-021-01604-9)
Supplement: Supplementary file 5 — Supplementary file5 (DOCX 49 kb) [file 40279_2021_1604_MOESM5_ESM.docx]

**Supplementary Table S5.** Details of studies and information relevant to cardiorespiratory outcomes.

| **Study** | **Cohort/ sample size (n), sex, age** | **Study purpose** | **Outcome Measures** | **Exercise Protocol** | **Compression worn during/after/both** | **Compression pressure – reported value or not stated** | **Key findings** |
| --- | --- | --- | --- | --- | --- | --- | --- |
| Ali et al., 2010 | 10 triathletes, 1F and 9M, (36.0 ± 10.0 y) | To examine the physiological and perceptual responses to wearing graduated compression stockings during fast-paced running in a controlled laboratory environment. | Oxygen uptake | 90% of 10 km personal best speed at 1% incline for 40 mins on treadmill | During | Low compression garment: Calf: 12 mmHg, Ankle: 15 mmHg  High compression garment: Calf: 23 mmHg, Ankle: 32 mmHg | Runners experienced no physiological benefits while wearing graduated compression garments in terms of improved running economy or reduced heart rate. |
| Berry & McMurray, 1987 | Experiment 1: 6 fit college students, M,  (22.5 ± 5.4 y)  Experiment 2: 6 healthy college students, M, (21.4 ± 4.3 y) | The first study was designed to determine the effects of GCS on maximal oxygen consumption, time to exhaustion during a test for VO2 max and blood lactate levels during recovery from a test of VO2max. The second study was designed to evaluate retention of lactate by manipulation of the GCS at the end of exercise. | Oxygen consumption, expired O_2_ and CO_2_, VO_2_max. | 15 min at treadmill speed set to elicit a heart rate of 130 bpm, after which time the grade was increased every 2 min by 2.5% until exhaustion | During | Calf: 8mmHg  Ankle: 18mmHg | There were no differences in any of the experiments when comparing recovery oxygen uptake between trials. |
| Born et al., 2014 | 10 elite German ice speed skaters, 6F and 4M,  (23.0 ± 7.0 y) | To investigate whether the application of compression to both legs in elite ice speed skaters improves muscle oxygenation and blood volume, ratings of perceived exertion; blood lactate concentration; and, 3000-m time. | Gas exchange data, O_2_ uptake. | 3000-m race simulation | During | Thigh: 20.3 ± 2.3 mmHg, Calf: 24.4 ± 3.1 mmHg | Compression provided no effect on selected cardiorespiratory and circulatory parameters during 3000-m ice speed skating. |
| Born et al., 2014 | 12 track and team sport athletes, F, (25.0 ± 3.0 y) | To assess the effects of compression garments with silicone stripes (which mimic kinesio taping) on repeated sprint performance | O_2_ uptake, ventilation, | 30 x 30-m sprints (1 sprint per minute) | During | *Gluteus maximus*: 18.3 ± 4.1 mmHg, *Rectus femoris*: 19.0 ± 4.9mm Hg, *Vastus lateralis*: 17.5 ± 4.4 mmHg, *Bicep femoris*: 19.6 ± 4.7 mmHg, *Gastrocnemius medialis*: 21.7 ± 6.0 mmHg | There were no significant differences in physiological measures between conditions. |
| Bringard et al., 2006 | Test 1: 6 trained runners, M, (31.2 ± 5.4 y)  Test 2: 6 trained runners, M, (26.7 ± 2.9 y). | Experiment 1: To examine the effect of wearing compression tights compared to wearing shorts and classic tights on aerobic energy cost of running at various submaximal running intensities.  Experiment 2: evaluate the effects of wearing compression tights on the excess in VO_2_ during prolonged submaximal exercise. | Exp 1: VO_2_, CO_2_ production, RER, Ve  Exp 2: VO_2_ slow component | Exp 1: continuous incremental exercise test to voluntary exhaustion  Exp 2: 15 min run at 80% VO_2max_ | During | NS | Wearing compression tights decreased the energy cost of running at some submaximal intensities. Additionally, aerobic cost was lower with compression garments than when wearing shorts (by 36%) and classic tights (by 26%) during prolonged submaximal exercise. Other components were unaffected by compression. |
| Broatch et al., 2017 | 20 recreationally active, 11F (25.0 ± 2.0 y) and 9M (28.0 ± 6 y) | This study aims to assess the effects of lower-limb compression garments on markers of blood flow, oxygen kinetics, and exercise performance during a repeated sprint protocol with short rest intervals | Absolute VO_2_ | 4 sets of 10 x 6-s maximal sprints, inter-spaced by 24 s of recovery between bouts and 2 min recovery between sets | During | Thigh: 11.7 ± 2.3 mmHg, Calf: 26.4 ± 6.4 mmHg, Ankle: 21.5 ± 8.2 mmHg | Lower-limb compression garments worn during repeated sprint exercise improved hemodynamic variables. |
| Brophy-Williams et al., 2018 | 12 runners, M, (30.5 ± 8.1 y) | Assess the effect of wearing compression socks during a 5km running time trial on physiological, perceptual and performance-based parameters. In addition, the capacity for compression socks to impact subsequent performance was also investigated. | VO_2_ | A 5 km time trial, a one-hour recovery period, then a repeat of the warm-up and 5 km time trial | During the first warm up and time trial | Compression socks:  Calf: 37 ± 4 mmHg, Upper ankle: 31 ± 4 mmHg, Lower ankle: 23 ± 4 mmHg | No difference in any outcome measure apart from a small decrement in repeated time trial performance. |
| Broatch et al., 2020 | 27 recreational-active, M  Study 1, n=13 (22.0 ± 3.0 y),  Study 2, n=14 (27.0 ± 5.0 y) | To investigate the effectiveness of three different commercially-available lower-limb sports compression garments in reducing muscle displacement, soft-tissue vibrations, and muscle activation during running at different speeds | Oxygen consumption | Study 1: 4-min treadmill running bouts (2 min at 12 km/h and 15 km/h)  Study 2: 9-min treadmill running bouts (3 min at 8 km/h, 10 km/h, and 12 km/h) | During | 6 pressure sites all measured in mmHg: a) 5 cm proximal to medial malleolus, b) 5 cm proximal to a), c) maximal calf girth, d) thigh 10 cm below land- mark, e) midthigh, f) 5 cm proximal to landmark e.  2XU Tights: a) 13.2 ± 2.9, b) 17.2 ± 6.2, c) 21.8 ± 6.0, d) 12.0 ± 2.2, e) 12.1 ± 2.3, f) 10.7 ± 2.9 mmHg  Nike Pro Zonal Tights: a) 9.1 ± 2.5, b) 14.6 ± 4.9, c) 21.5 ± 5.1, d) 11.3 ± 2.1, e) 12.9 ± 2.7, f) 12.7 ± 2.1 mmHg  Under Armor Charged Tights: a) 7.7 ± 3.1, b) 11.4 ± 4.7, c) 18.9 ± 6.3, d) 13.3 ± 3.1, e) 13.2 ± 3.1, f) 12.6 ± 2.9 mmHg | Compression tights worn during submaximal treadmill running had no effect on running economy was observed. |
| Cheng and Xiong 2019 | 16 healthy participants, M, (22.5 ± 0.9 y) | To explore the effects of compression stockings on metabolic cost, muscle activation, kinematics and joint kinetics during walking | Metabolic rate | Participants walked at a speed of 5 km/h for 6 min.  Participants walked along a 10 metre level walkway following a metronome. | During | 30 – 40 mmHg | Compression stockings have trivial effects on the metabolic cost and kinematics. |
| Dascombe et al., 2011 | 11 well-trained middle-distance runners and triathletes, M, (28.4 ± 10.0 y) | To determine the effectiveness of wearing an undersized lower body compression garment on physiological and performance parameters relating to endurance running. | VO_2_max, Expired Gas Analysis | Time to exhaustion test consisting of the participant running at 90% of VO_2max_ velocity until volitional exhaustion | During | Regular size, thigh: 13.7 ± 2.3 mmHg, Calf: 19.2 ± 3.2 mmHg  Undersize, yhigh:  15.9 ± 2.6 mmHg, calf: 21.7 ± 4.3mmHg | At slower running velocities, both lower body compression garment conditions significantly increased muscle blood flow and O_2_ utilisation and O_2_ pulse compared with the control condition. During the faster running velocities (>12 km⋅h-1), both lower body compression garment conditions significantly increased deoxyhemoglobin concentration within the vastus lateralis, which coincided with a decrease in heart rate and tissue oxygen index. |
| Dascombe et al., 2013 | 7 elite flat-water kayakers, 2 F (25.0 ± 4.2 y) and 5 M (21.8 ± 2.8 y) | To determine the effects of wearing upper body compression garments on selected performance responses during simulated flat-water kayaking in elite kayakers, and to identify changes in the physiological responses during simulated kayaking that may be responsible for changes in performance. | Expired gas | Six-step incremental test and a subsequent 4min performance test on a kayak ergometer | During | NS | No significant improvements in the selected physiological measures during simulated flatwater kayaking in elite kayakers wearing the upper body compression garments. |
| Ehrstrom et al., 2018 | 13 trail runners, M, (38.6 ± 5.7 y) | To examine whether wearing high-pressure compression garments (>15 mmHg) during a 40-min treadmill downhill run on acute and delayed neuromuscular responses and running economy in well-trained trail runners accustomed to eccentric work | VO_2_, RER and Ve | 40-min downhill running at –8.5 deg decline | During | Middle calf: 20-25 mmHg, upper calf: 18-20 mmHg, middle thigh: 16-18 mmHg, lower thigh: 18-20 mmHg | The use of high-pressure compression garments during downhill running induces beneficial effects on physiological responses. |
| Fujii et al., 2017 | 9 participants, M, (24.7 ± 2.0 y) | To examine whether stocking-mediated graduated compression augments cutaneous vasodilation but not sweating during exercise in the heat. | Minute ventilation, tidal volume, respiratory frequency, end-tidal CO_2_ pressure, O_2_uptake, CO_2_ output, and respiratory exchange ratio were recorded over 30-sec periods. | Participants performed cycling at 60% peak oxygen uptake at a pedalling rate of 60 rpm for 45 min or until body core temperature reached ~1.5°C above baseline resting values | During | Ankle: 26.4 ± 5.3 mmHg, calf: 17.5 ± 4.4 mmHg, thigh: 6.1 ± 2.0 mmHg | Graduated compression stockings induced no differences in respiratory responses. |
| Glanville and Hamlin 2012 | 14 trained multisport, M, (33.8 ± 6.8 y) | To determine the effects of wearing commercially available graduated compression garments during prolonged recovery (24hours) on subsequent 40-km cycling time trial performance in trained multisport athletes. | Ventilation & expired gas, O_2_ cost. | 40-km cycling performance | Post-exercise for 24 hours | Upper ankle: 6.0 ± 2.4 mmHg, upper calf: 14.7 ± 2.5 mmHg, upper leg segment: 11.8 ± 2.5 mmHg | Compression provided no effect on physiological measures post-exercise. |
| Houghton et al., 2009 | 10 amateur field hockey, M,  (21.0 ±2 .0 y) | To investigate the effects of compression garments on thermoregulation in field hockey players. | VO_2_max | The Loughborough intermittent shuttle test | During | Compression Shorts and short sleeved top - NS | Only skin temperature was significantly higher in compression than control.  Similar heart rate between trials. |
| Kemmler et al., 2009 | 21 moderately trained runners, M, (39.3 ± 10.7 y) | To determine the effect of below-knee stockings with constant compression on selected parameters of running performance in healthy male runners | VO_2_, CO_2_ production, Ve, RER | Stepwise-speed incremental running test | During | Ankle: ~24 mmHg, calf: ~18 - 20 mmHg | Compression provided no effect on physiological measures during exercise |
| Leoz-Abaurrea et al 2016 | 10 recreational runners, M, (23. 0± 3.0 y) | To analyse the physiological responses of heat dissipating upper body compression garments during a running performance test to exhaustion | V̇O_2_, VCO_2_, RER, Ve, | 45-min run at 60% of the peak treadmill speed followed by a time to exhaustion run at 80% of the peak treadmill speed | During | *Biceps brachii*: 2.9 ± 1.5 mmHg, *triceps*: 3.0 ± 1.0 mmHg, *pectoralis major*: 2.0 ± 0.5 mmHg, *latissimus dorsi*: 1.4 ± 0.5 mmHg | Upper body compression garments impaired cardiorespiratory responses during exercise. |
| Leoz-Abaurrea et al 2015 | 13 untrained participants, M, (21 ± 6 y) | To investigate whether a heat dissipating upper body compression garment can mitigate thermoregulatory strain better than non-compression garments during cycling in hot (i.e., 40 degree) temperatures. | VO_2_, VCO_2_, Respiratory exchange ratio | Cycling at a fixed workload (~50% VO_2peak_) with 4 bouts of 14 minutes at 40 ºC with each bout separated with a minute active recovery | During | NS | Upper body compression garments impaired cardiorespiratory responses during recovery. Additionally, a significantly lower reduction in heart rate occurred during active recovery |
| Leoz-Abaurrea et al 2016 | 16 untrained participants, 4F and 12M, (21.3 ± 5.7 y) | To determine the effects of upper body compression garments on thermoregulatory responses during cycling in a controlled laboratory thermoneutral environment (~23°C). A secondary aim was to determine the cardiovascular and perceptual responses when wearing the garment. | Respiratory gas exchange | Cycling at a fixed workload (~50% VO_2peak_) with four bouts of 14 minutes at 40 ºC with each bout separated with a minute active recovery | During | NS | Wearing an upper body compression garment in a thermoneutral environment did not alter cardiorespiratory responses. |
| Leoz-Abaurrea et al 2017 | 12 trained individuals, M, (66.0 ± 2.0 y) | To evaluate the effects of an upper body compression garment vs. a control garment (CON) on thermoregulatory responses in trained older adults in a temperate environment. | Respiratory gas exchange | Cycling trial consisted of four bouts at a fixed load (50% peak power output) for 14 minutes, with each separated with a minute rest | During | Arm: ~1-3 mmHg | Results showed that wearing an upper body compression garment led to a significantly higher core and body temperature at the end of exercise. |
| Lovell et al., 2011 | 26 semi-professional rugby league players, M, (21.6 ± 2.5 y) | To examine the effect of compression garments on active post-exercise recovery after a bout of high-intensity exercise | VO_2max_, Ventilation, RER | A 6-stage submaximal treadmill test which consisted of 5-minute stages at 6 km/h, 10 km/h, approximately 85% of VO_2max_, and 6 km/h as a recovery stage followed by approximately 85% of VO_2max_ and 6 km/h | During | Ankle: 20 ± 2 mmHg, calf: 15 ± 2 mmHg | Heart rate was lower when wearing compression garments during active recovery after a bout of high-intensity running, with no difference in cardiorespiratory measures. |
| Mizuno et al., 2017 | 30 physically active participants  Compression thigh group, 10M,  (21.3 ± 0.4 y)    Compression sock group 10M, (21.6 ± 0.8 y)  Control group, 10M, (22.9 ± 0.7 y) | Examine the effects of the body coverage area of compression garments on the exercise performances and muscle damage during prolonged running | Expired gas analysis | 120min of uphill running at 55% of ˙VO_2max_ | During | Thigh compression group:  14.7 ± 0.6 mmHg  Calf compression group: 17.4 ± 0.5 mmHg  Control group: Thigh: 3.0 ± 0.3 mmHg and calf: 1.8 ± 0.2 mmHg | The present findings revealed no significant effects of the body coverage area of the compression garments on physiological measures. |
| Okamoto et al., 2012 | 10 healthy, 4F and 6M, (29.8 ± 5.9 y) | Investigate the acute effect of brisk walking with and without graduated compression garments on vascular endothelial function and oxidative stress. | O_2_ uptake | 30 min treadmill walking at an intensity of ~60% of their individually determined heart rate reserve | During | Ankle: 25 mmHg, calf: 17 mmHg | Graduated compression garments suppress the decrease in brachial artery flow mediated dilation and increases biological antioxidant potential.  No effect on heart rate was observed. |
| Priego et al., 2015 | 20 recreational runners, 7F and 13M, (28.1±5.4y) | The aim of this study was therefore to analyse the effects of running with and without graduated compression garments for three weeks on different cardiorespiratory parameters in runners. | Ve, Ve/O_2_ and Ve/VCO_2_ | 30 min running at 80% of maximal aerobic speed | During | Ankle: 24 mmHg  Calf: 21 mmHg | No effect on any of the cardiorespiratory parameters was observed after three weeks of running with graduated compression garments. |
| Rider et al., 2014 | 10 Division III cross-country runners, 3F (18.7 ± 0.6 y) and 7M (21.0 ± 1.3 y) | To determine whether wearing below-the-knee graduated compression stockings with a minimum of 15 mmHg of pressure during a maximal treadmill run would induce physiological changes among collegiate cross-country runners. | VO_2_max and RER | 5-km running time trial | During | Ankle: 20 mmHg  Calf: 15 mmHg | Compression provided no effect on physiological measures. |
| Rimaud et al., 2010 | 8 endurance trained, M,  (27.1 ± 0.9 y) | This study aimed to investigate if wearing compression stockings during exercise and recovery could affect lactate profile in sportsmen. | VO_2_, VCO_2_ and VO_2max_ | Incremental cycling to exhaustion | During and 60 mins after | Ankle: 12 mmHg  Calf: 22 mmHg | Compression had no influence physiological measures during and post-exercise. |
| Rivas et al., 2016 | 13 runners. 3F and 10M, (20.9 ± 2.5 y) | To determine if commercially available below the knee lower leg compressions would improve resting/submaximal/maximal exercise test cardiorespiratory measurements (heart rate, breathing rate, ventilation, oxygen uptake), lactate metabolism, and perception of exercise intensity during running in endurance-trained athletes | BR, VE, ventilatory efficiency VO_2_, running economy and RER | The incremental graded exercise test consisted of baseline rest and submaximal intensities at 23%, 70%, 75%, 85% and then a progressive increase to 100% VO_2max_ | During | Compression socks ankle: 12–15 mmHg, calf: 9–12 mmHg | Leg compression improves Ve but does not improve aerobic exercise capacity in endurance-trained runners. |
| Scanlan et al., 2008 | 12 well trained cyclists, M, (20.5 ± 3.6 y) | To investigate the effects of wearing lower body compression garments on physiological and performance responses during endurance cycling. | O_2_ and CO_2_ concentrations, VO_2_ | 1-hour time trial and incremental test | During | *Posterior gluteus maximus*: 9.1 ± 2.2 mmHg, *vastus lateralis*: 14.9 ± 2.3 mmHg, calf: 17.3 ± 3.0 mmHg, ankle: 19.5 ± 3.4mmHg | Compression provided no effect on physiological measures during exercise. |
| Smale et al., 2017 | 15 trained cyclists, M, (28.1 ± 6.3 y) | Examine the effects of varying grades of compression garments during incremental cycling exercise on cerebral artery blood flow velocity and its mechanistic physiological regulators, and cognitive performance in well-trained cyclists. | Respiratory gas analysis and P_et_CO_2_ | 4 x 8 min increments of cycling at 30%, 50%, 70%, and 85% peak power output and a 4 km time trial | During | Medium grade garment at the ankle: 21.8 ± 6.6 mmHg, knee: 20.3 ± 6.6 mmHg, and thigh: 15.4 ± 4.5 mmHg  Low grade compression: ankle: 8.6 ± 2.7 mmHg; knee: 14.9 ± 4.9 mmHg; and thigh: 9.1 ± 3.1 mmHg | Compression provided no effect on selected cardiorespiratory and circulatory parameters. |
| Sperlich et al., 2010 | 15 runners and triathletes, M, (27.1 ± 4.8 y) | Assess the effects of three types of compression clothing (socks, tights, and whole-body compression) on physiological responses and effects on performance. | VO_2_ | 15 min run at 70% VO_2max_. Following this, running speed was set at the highest speed achieved during incremental testing and participants ran to volitional exhaustion. | During | Calf: 20 mmHg – Not measured but aimed for this pressure | No differences between compression clothing and clothing without external pressure. |
| Sperlich et al., 2013 | 12 elite alpine skiers, M, (26.0 ± 4.0 y) | To evaluate the effects of different levels of compression on the legs of highly trained alpine skiers subjected to passive vibration in the downhill tuck position. | O_2_ uptake, CO_2_ output, RER, Ve and breathing frequency. | 3-min trials in a downhill tuck position involving application of passive vibration to the soles of both feet | During and five minutes after | Moderate compression calf: 19.7 ± 3.7 mmHg, thigh: 17.8 ± 1.9 mmHg; High compression calf: 39.5 ± 3.5 mmHg, thigh: 34.0 ± 2.6 mmHg | Greater deoxygenation of the vastus lateralis but no differences in whole-body oxygen consumption or other physiological measures with compression. |
| Sperlich et al., 2013 | 10 well-trained endurance athletes, M, (25.0 ± 4.0 y) | to assess whether upper body compression garments improve double-poling sprint performance (three 3-min simulated DP sprints on a cross-country ski ergometer) by enhancing power output and improving selected metabolic, cardio-respiratory, haemodynamic, and perceptual parameters | Oxygen uptake and production of carbon dioxide. | 3 x 3-min simulated double polling sprints on a cross-country ski ergometer | During | Forearm: 21 ± 5 mmHg, *Triceps brachii*: 14 ± 3 mmHg, *Biceps Brachii*: 14 ± 2 mmHg, *Latissimus dorsi*: 9 ± 2 mmHg | Upper-body compression revealed no influence on most of the parameters related to the metabolic, cardiorespiratory, and hemodynamic responses examined. |
| Mizuno et al., 2017 | 8 physically active, M, (23.4 ± 2.4 y) | To investigate the effect of wearing lower body compression garments exerting different pressure levels during prolonged running on exercise-induced muscle damage and the inflammatory response. | Perceived respiration | 120 min of uphill running at 60% of VO_2max_ | During | High pressure garment: Thigh: 26.9 ± 3.3 mmHg; Calf: 29.2 ± 3.8 mmHg  Medium pressure garment: Thigh: 16.1 ± 2.0 mmHg; Calf: 17.9 ± 3.5 mmHg  Control garment:  Thigh: 4.4 ± 1.2 mmHg; Calf: 3.0 ± 1.6 mmHg | The medium pressure showed a significantly smaller increase in heart rate compared with that in the control trial. |
| Stickford et al., 2015 | 16 trained runners, M, (22.4 ± 3.0 y) | Investigate the impact of wearing lower-leg compression sleeves on the running economy and mechanics of highly trained distance runners. | Ve, fraction of O_2_ and CO_2_, VE, VO_2_ and VCO_2_ | Four minute stages at each of three constant submaximal speeds of 233, 268, and 300 m/min on a motorized treadmill | During | Calf: 15-20 mmHg | Lower-leg compression sleeves did not alter whole-body VO_2_ during submaximal running, leg stiffness, running mechanics, or metabolic response when compared to control.  Those who improved running economy while wearing compression had lower measures of gait variability, particularly at the slowest speed |
| Varela-Sanz et al., 2011 | 16 well trained runners, 3F (32.0 ± 4.6 y) and 13M (35.4 ± 6.6 y) | To assess the influence of beneath-knee gradual elastic compression stockings on running economy and performance at competitive velocities in a group of well-trained runners | Experiment 1: VO_2_  Experiment 2: VO_2_ | Running to exhaustion at 1% incline and 105% of recent 10-km time (17 ± 2 km/h) | During | Ankle: 15 – 22 mmHg | Percentage of heart rate max reduced during a time trial at near competition pace, also showing a tendency to improve endurance time to fatigue and VO_2peak_. |
| Vercruyssen et al., 2017 | 12 competitive trail runners, sex NS, (39.6 ± 4.6 y) | The influence of wearing compression garment vs. conventional running clothing on muscle contractile function and running economy before and after a short distance trail running. | O_2_ uptake | 18.4-km short distance trail runs | During | Ankle: 18 mmHg, calf: 7.5 mmHg | There were no benefits from wearing compression garments on physiological variables following short distance trail running. |
| Williams et al., 2020 | 10 trained university-level cyclists, M, (21.0 ± 2 y) | To assess the effects of varying levels of compression applied via lower-limb compression garments on multiday cycling performance at typical levels of exercise induced muscle damage associated with multiday exercise events | Pulmonary gas exchange and ventilation | High intensity protocol, 24h rest, then an 8km time trial | During | Low-pressure compression garment:  distal hem: 7 ± 3 mmHg,  calf: 7 ± 3 mmHg,  mid-thigh: 5 ± 2 mmHg,  head of femur: 5 ± 2 mmHg,  posterior superior iliac spine: 5 ± 1 mmHg  High-pressure compression garment:  distal hem: 11 ± 3 mmHg,  calf: 15 ± 3 mmHg,  mid-thigh: 10 ± 3 mmHg,  head of femur: 8 ± 2 mmHg,  posterior superior iliac spine: 6 ± 1 mmHg | No physiological or subjective mechanistic explanations were present |

M = Male, F = Female, NS = Not specified, HRmax = Max heart rate, VO_2max_ = Maximal oxygen uptake, O_2_ = Oxygen, CO_2_ = Carbon Dioxide, Ve = minute ventilation , VO2 = Oxygen consumption, VCO2 = Carbon dioxide production, Ve/O2 = Ventilatory equivalent for oxygen, Ve/VCO2 = Ventilatory equivalent for carbon dioxide, RER = Respiratory exchange ratio, P_et_CO_2_ = Partial pressure of end tidal CO_2_
