# Supplementary material for: Putting the Squeeze on Compression Garments: Current Evidence and Recommendations for Future Research: A Systematic Scoping Review
Source: Sports Med. 2021 Dec 6;52(5):1141–60. doi: 10.1007/s40279-021-01604-9 (PMC9023423; doi:10.1007/s40279-021-01604-9)
Supplement: Supplementary file 6 — Supplementary file6 (DOCX 35 kb) [file 40279_2021_1604_MOESM6_ESM.docx]

**Supplementary Table S6.** Details of studies and information relevant to muscle damage and swelling outcomes.

| **Study** | **Cohort/ sample size (n), sex, age** | **Study purpose** | **Outcome Measures** | **Exercise Protocol** | **Compression worn during/after/both** | **Compression pressure – reported value or not stated** | **Key findings** |
| --- | --- | --- | --- | --- | --- | --- | --- |
| Areces et al., 2015 | 34 marathon runners, 4F and 30M,  Control group: (42.7 ± 7.8 y), Compression group: (41.2 ± 8.9 y) | Investigate the benefits obtained by wearing graduated compression stockings on running pace, prevention of muscle damage and maintenance of muscle performance during a real marathon race. | lower leg volume | Marathon race | During | The highest pressure was at the foot and the malleolus and it decreased proximally, from 25 mmHg to 20 mmHg | Outcomes were the same in the compression group and control group |
| Broatch et al., 2019 | 12 elite Australian volleyball athletes, F, (25 ± 2 y) | To determine the effects of wearing compression socks during long-haul travel on sports-specific performance, physiological, and haematological alterations in elite female volleyball athletes | calf girth | Air travel | Duration of travel | Max calf girth: 23 ± 11 mmHg  Ankle: between 19 and 22 (±8) mmHg | Decreased calf girth with compression |
| Brophy-Williams et al., 2016 | 12 well trained runners, M, (30.5 ± 8.1 y) | To assess the effect of wearing compression socks during a one-hour recovery period following a 5km running time trial on performance in a subsequent 5km time trial | cross sectional area | A 5km TT, then a one-hour recovery intermission before a second warm-up and 5km TT. | Between time trials | Max calf girth: 23 ± 11mmHg  Upper ankle: 22 ± 8mmHg  Lower ankle: 19 ± 8mmHg | Compression may have reduced muscle cross sectional area during recovery period. |
| Brown et al., 2020 | 45 Recreational rugby players, M, 13 custom fit (24.0 ± 6.0 y), 16 standard size (23.0 ± 3.0 y) and 16 control (22.0 ± 4.0 y) | To evaluate the effects of compression garments applying different pressures on muscular recovery after EIMD in rugby players | mid-thigh girth swelling | 20x20m sprints, 100 drop jumps | 48 hours post exercise | Custom fit:  Ankle: 32 ± 3 mmHg  Calf: 24 ± 4 mmHg  Thigh: 19 ± 3 mmHg  Standard size:  Ankle 11 ± 5 mmHg  Calf: 10 ±3 mmHg  Thigh: 7 ± 3 mmHg | Custom-fitted CG designed to apply higher pressures than commercially available garments reduced muscle swelling |
| Carling et al., 1995 | 23 participants,  16F and 7M, (26 ± 4 y) | Evaluate the effect of applying external compression on DOMS and the accompanying manifestations of soreness, swelling, range of motion, and isokinetic strength for a period of 4 days following induction of DOMS | oedema circumference and volume | 70 eccentric contractions of the elbow flexor muscles, dynamometer test (120degs/sec through 120 degs of motion) | After | Sleeve pressure: 17 mmHg | No significant differences were present for either group. |
| Coza et al., 2012 | 16 physically active, M, (26.3 ± 5.1 y) | To to quantify the effects compression on the gastrocnemius medialis muscle energy use during short-term activity and gastrocnemius medialis tissue oxygenation at the beginning of exercise. | calf circumference | 40 heel raises per min for 2 min | During | NS | Externally applied compression does not have a significant impact on calf circumference. |
| Davies et al., 2009 | 7 female netball players and 4 male basketball players, 7F (19.7 ± 0.5 y) and 4M (26.3 ± 5.1 y) | To investigate whether wearing compression tights for 48 hours following plyometric exercise would attenuate muscle damage markers and muscle soreness, and sprinting and jumping performance | mid-thigh circumference | 5 sets of 20 drop jumps from a platform 60 cm high followed immediately by a maximal upward jump, with a 2-minute rest period between sets | For 48 hours afterwards | 15 mmHg from the lower to the upper legs | Results indicate that CG demonstrated no benefit on muscle swelling |
| Driller and Halson 2013 | 12 highly trained male cyclists, M, (30 ± 6 y) | To investigate the effect of wearing lower body compression garments on performance during a 30-minute endurance cycling test in highly trained cyclists. A further aim of the study was to determine various physiological and perceptual responses when wearing compression garments during the cycle test. | calf girth | 15 minutes at a workload equal to 70% PPO, followed immediately by a 15-minute time trial. | During | ~18 mmHg at the medial malleolus decreasing to ~10 mmHg at the gluteus maximus | There were no significant differences in calf girth conditions. |
| French et al., 2008 | 26 participants, M, (24.1 ± 3.2 y) | To evaluate contrast bathing and contrast garments as regeneration strategies after EIMD | Midthigh and midcalf circumference | 6 x 10 squats with 100% of body mass + 5 second eccentric repetition with the participants 1RM | For 12 hours after exercise | Calf: 12 mmHg  Thigh: 10 mmHg | Results indicate that no intervention demonstrated a clear benefit on muscle circumference |
| Geldenhuys et al., 2019 | 41 marathon runners,  Experimental group, 6F and 14M, (34 ± 4.8 y)  Control group,  6F and 15M, (34 ± 6.4 y) | To determine the impact of below-knee CGs on lower leg EIMD and performance in runners before, during, and after an ultramarathon road race | Pennation angle, muscle thickness, ankle and midcalf circumference | 56-km ultramarathon | During | NS | No changes were detected in calf and ankle circumferences in the 6-week training period. However, reduced ankle circumferences were detected 2 days post-race. |
| Goto and Morishima 2014 | 9 participants, M, (21.0 ± 0.4 y) | To investigate the effects of wearing a compression garments for 24 h on the changes in muscular strength and blood parameters over time after resistance exercise. | Muscle circumference | Six exercises for the upper body and three for the lower body muscles. Each exercise set comprised 10 repetitions involving five sets for bilateral leg press and bilateral knee extension and three sets for the remaining seven exercises. | Worn for 24 hours after resistance training | NS | Wearing a CG after strenuous resistance exercise did not influence muscle swelling |
| Heiss et al., 2018 | 15 participants, 7F and 8M, (25 ± 6 y) | To investigate the influence of commercially available sport compression garments on the development of exercise-induced intramuscular oedema | intra-muscular oedema, calf circumference | All participants performed 5 sets of 30 repetitions of calf raises and rested 10 sec between each set with 25% of their body weight during the exercise. | The compression sock was worn continuously for 60 h after eccentric exercise and was removed for the first time for follow-up examination | NS | There was no significant effect on intra-muscular oedema or calf circumference |
| Kraemer et al., 2001 | 20 non-strength-trained participants, F,  Compression sleeve group (21.3 ± 2.9 y),  Control group,  (21.1 ± 3.3 y) | To investigate whether constant compression via the use of a compressive arm sleeve would reduce the severity and duration of soreness associated with DOMS. | arm circumference | Dynamometer (60°/s). 2 sets of 50 repetitions with 3 minutes rest between sets. Every fourth repetition, the subject performed a maximal concentric contraction with an isometric hold followed by an eccentric contraction | After | 10 mmHg | Compression was found to prevent swelling |
| Kraemer et al., 2010 | 20 resistance trained subjects, 9F (23.1 ± 2.2 y) and 11M (23.0 ± 2.9 y) | To evaluate the influence of a whole-body compression garment on recovery from a typical heavy resistance training workout | Vastus lateralis swelling thickness, patella tendon thickness,  oedema upper arm, forearm, upper leg, lower leg, ankle circumference. | 3 sets at 8-10RM of back squats, bench press, stationary lunge, bent-over row, Romanian deadlift, biceps curl, sit up, high pull from hang. | For 24 hours after exercise | NS | Whole-body CG did produce more rapid recovery of selected physiological variables |
| Marqués-Jiménez et al., 2017 | 18 semi-professional football players, M, (24.7 ± 4.07 y) | To evaluate the influence of wearing different types of compression garments during matches and recovery after a friendly soccer match | muscle swelling | Soccer match | Played the match wearing one type of graduated compression garment, and kept wearing them 7 h/day during 3 days post-match (players put them on each day after the testing session). | Compression stockings:  Ankle: 20–25 mmHg Calf: 15–20 mmHg  Compression tights: Calf: 25–30 mmHg  Thigh: 15–20 mmHg  Compression shorts: Thigh: 15–20 mmHg | Compression garments used during and post a soccer match helped attenuate muscle swelling in compressed muscle only |
| Miyamoto and Kawakami 2015 | 15 healthy participants, M, (25.2 ± 2.6 y) | To examine the effect of pressure profiles of compression stockings on muscle fatigue level of the lower leg muscles induced by running exercise, and to test the pressure profiles (targeting the gastrocnemius) against the development of muscle fatigue. | MR-T2 | Running on a treadmill set at 0- inclination for 34.5 min including 4.5-min warm-up, 1.5 min at 6 km, 1.5 min at 8 km, 1.5 min at 10 km, and 30 min at 12 km | During | Graduated low pressure compression condition G*astrocnemius*: 14 mmHg  Ankle:18 mmHg  Graduated high pressure compression condition: *Gastrocnemius*: 21 mmHg  Ankle: 27 mmHg  Uniform pressure distribution condition: *Gastrocnemius* = 21 mmHg Ankle = 21 mmHg   Gastrocnemius region condition: *Gastrocnemius*: 21 mmHg Ankle: 10 mmHg | Wearing CG improve T2 relaxation time during submaximal running exercise |
| Miyamoto et al., 2014 | Two groups of 11 participants, M, Experiment 1 (25.6 ± 3.7 y), Experiment 2 (27.0 ± 1.8 y) | To examine the effect of pressure intensity of elastic compression short-tights on the metabolic state of thigh muscles during submaximal running | MR-T2 Images | exercise on a treadmill set at 0- inclination for 34.5 min including 4.5-min warm-up running, 1.5 min at 6 km, 1.5 min at 8 km, 1.5 min at 10 km, and 30 min at 12 km | During | Two compression short-tights Low group: 8 mmHg High group: 15 mmHg  Mid-thigh: 20 mmHg  Thigh group: 25 mmHg | Wearing compression short tights improve T2 relaxation time during submaximal running exercise |
| Mizuno et al., 2016 | 18 participants, M, (21.9 ± 0.6 y) | To determine the effects of wearing a lower body compression garment for 24 h following running (either downhill or level) in terms of recovery of exercise performance, muscle damage, inflammatory markers in the blood, and subjective muscle soreness and fatigue. | thigh and calf circumference | 30 min of downhill running | 24 hours post exercise | Compression group: Thigh: 11.5 ± 0.6 hPa Calf: 17.6 ± 1.8 hPa  Control group:  Thigh: 7.1 ± 1.3 hPa Calf: 11.5 ± 2.1 hPa | Muscle swelling was not influence using CG for 24 h post-exercise. |
| Mizuno et al., 2017 | 30 physically active participants  Compression thigh group, 10M,  (21.3 ± 0.4 y)    Compression sock group 10M, (21.6 ± 0.8 y)  Control group, 10M, (22.9 ± 0.7 y) | Examine the effects of the body coverage area of compression garments on the exercise performances and muscle damage during prolonged running | Thigh and calf circumference | 120min of uphill running at 55% of ˙VO_2max_ | During | Thigh compression group: 14.7± 0.6 mmHg  Calf compression group: 17.4 ± 0.5 mmHg  Control group:  Thigh: 3.0 ± 0.3 mmHg Calf: 1.8 ± 0.2 mmHg | The present findings revealed no significant effects of CG on thigh and calf circumference |
| Montgomery et al., 2008 | 29 basketball players, M, (19.1 ± 2.1 y) | To (1) investigate the time course of muscle damage markers and inflammatory cytokines during basketball tournament play and (2) assess whether cold water immersion and compression recovery strategies ameliorate any post-game increases of these biomarkers, compared with traditional refuelling and stretching routines | Mid-thigh circumference | 3-day mini-tournament involving one full 48-min game per day | For ~18 hours post-game | ~18 mmHg | There were no benefits from wearing compression garments on muscle swelling |
| Pereira et al., 2014 | 22 resistance trained participants, M, (24.6 ± 5.1 y) | To examine the effect of graduated compression sleeves worn during exercise on muscle recovery in young resistance trained men. | echo intensity (ultrasound) | 4 sets of 10 maximal elbow flexion/extension at 120˚/s. 1 minute separated sets. | During | NS | No significant differences between groups for echo intensity across 96 hours post exercise. |
| Santos Cerqueira et al., 2014 | 13 untrained participants, M, Compression group (22 ± 1 y),  Control group  (20 ± 1 y) | To assess the efficacy of compression sleeves worn for short-time periods (12h), on recovery from the symptoms of exercise induced muscle damage in the upper arm muscles | upper arm circumference | 30 repetitions of eccentric dumbbell curls which lasted 4-5 seconds | 12 hours following | NS | No significant differences between groups. |
| Terbizan et al., 2018 | 30 participants, M,  Control (21.56 ± 2.55 y),  Knee high stockings (21.80 ± 2.53 y),  Waist high tights (20.91 ± 1.92 y) | To compare the effects of waist-high compression garments and knee-high compression stockings for recovery from plyometric box drops. | Muscle thickness | 10 sets of 10 plyometric box drop jumps from 60cm box. Up to ten seconds were allowed between drops, and one minute between sets. | Worn for 12 hours post-exercise | NS | No effect of compression |
| Trenell et al., 2006 | 11 recreational athletes, M, (21.2 ± 3.1 y) | To observe the effects of 30-min of downhill walking on muscle metabolism and DOMS. The effect of compression garments applied after eccentric exercise was also studied. | βATP, αATP, γATP, PCr, PDE, Pi, PME | Participants performed a downhill walking protocol for 30 minutes on a treadmill (6 km·h^-1^, 25% grade). | For 48 hours following exercise | Calf: 16 - 17 mmHg Thigh: 10 mmHg | Compression garments resulted in a relative increase of PDE but had no effect on other measures. |
| Valle et al., 2013 | 15 amateur soccer players, M, (25.0 y) | To evaluate if there is a protective effect of compression against DOMS | intracellular albumin, CD3+, MPO intra/interfibrillar infiltrates, TIC | 40 minutes with a 10% downhill slope at 73% of the participants maximum speed | During | NS | A reduction in the amount and severity of the histological muscle damage in the compression group. |

M = Male, F = Female, NS = Not specified, CG= Compression Garment, 1RM = 1-Repetition maximum, TT = Time trial, PPO = Peak power output, VO_2max_ = Maximal oxygen uptake, , EIMD = Exercise induced muscle damage, PCr = phosphocreatine, PDE = phosphodiester, Pi = inorganic phosphate, PME = phosphomonoester, ATP = Adenosine tri-phosphate, MR = Magnetic resonance, T2 = skeletal muscle proton transverse relaxation times, CD3+ = Lymphocytes CD3+, MPO = Myeloperoxidase, TIC = Total inflammatory cells, DOMS = Delayed onset muscle soreness
