# Supplementary material for: Putting the Squeeze on Compression Garments: Current Evidence and Recommendations for Future Research: A Systematic Scoping Review
Source: Sports Med. 2021 Dec 6;52(5):1141–60. doi: 10.1007/s40279-021-01604-9 (PMC9023423; doi:10.1007/s40279-021-01604-9)
Supplement: Supplementary file 7 — Supplementary file7 (DOCX 29 kb) [file 40279_2021_1604_MOESM7_ESM.docx]

**Supplementary Table S7.** Details of studies and information relevant to thermoregulatory outcomes.

| **Study** | **Cohort/ sample size (n), sex, age** | **Study purpose** | **Outcome Measures** | **Exercise Protocol** | **Compression worn during/after/both** | **Compression pressure – reported value or not stated** | **Key findings** |
| --- | --- | --- | --- | --- | --- | --- | --- |
| de Glanville and Hamlin 2012 | 14 trained multisport athletes, M,  (33.8 ± 6.8 y) | To determine the effects of wearing commercially available graduated compression garments during prolonged recovery (24 hours) on subsequent 40-km cycling time trial performance in trained multisport athletes | Hydration | 40-km cycling performance | Post-exercise for 24 hours | Upper ankle: 6.0 ± 2,4 mmHg, Upper calf: 14.7 ± 2.5 mmHg, Upper leg segment: 11.8 ± 2.5 mmHg | Compression provided no effect on physiological measures post-exercise |
| Del Coso et al., 2013 | 36 experienced triathletes, Sex NS, Control group, n=17, (35.8 ± 6.3 y),  Compression group, n=19, (35.0 ± 5.3 y) | To investigate the potential of compression stockings to prevent muscular damage and to preserve muscular performance during a half-ironman competition | Core temperature | Half-iron man | During | NS | Compression garments did not alter body mass or heart rate |
| Doan et al., 2003 | 20 track athletes,  10F (19.2 ± 1.3 y), 10M (20.0 ± 0.9 y) | To determine how custom-fit compression shorts affect athletic performance and to examine the mechanical properties of the shorts. Specific performance and mechanical tests were designed to assess the effect of the garment on muscle oscillation, jump power, skin temperature, impact absorption and elasticity | Skin temperature | 60m sprint; CMJ; 60s intervals cycling | During | NS | Compression garment was related to increased skin temperature during a warm-up protocol |
| Duffield & Portus, 2007 | 10 club level cricket players, M, (22.1 ± 1.1 y) | To compare the effects of three different types of full-body compression garments and a control condition on performance in intermittent, repeat-sprint and throwing performance in cricket players | Skin temperature | Throwing and repeat-sprint performance | During and 24 hours after | NS | Compression garment was related to increased skin temperature during exercise |
| Duffield et al., 2008 | 14 rugby players, M, (19.0 ± 1.0 y) | To determine whether compression garments improve intermittent-sprint performance and aid performance or self-reported (perceptual) recovery from high-intensity efforts on consecutive days | tympanic temperature, thigh temperature | 4 x 15-min quarters of a simulated team game (exercise circuit) repeated across 2 days | During the simulated team games and for ~15 hours afterwards | NS | Heart rate, tympanic temperature, was higher with the garments |
| Fujii et al., 2017 | 9 participants, M, (24.7 ± 2.0 y) | To examine whether stocking-mediated graduated compression augments cutaneous vasodilation but not sweating during exercise in the heat. | Oesophageal and skin temperature, and chest sweat rate | Participants performed cycling at 60% peak oxygen uptake at a pedalling rate of 60rpm for 45 min or until body core temperature reached ~1.5°C above baseline resting values | During | Ankle: 26.4 ± 5.3 mmHg,  Calf: 17.5 ± 4.4 mmHg,  Thigh: 6.1 ± 2.0 mmHg | Graduated compression induced increases in cutaneous vasodilation during exercise in the heat. In contrast to cutaneous vasodilation, no effect on the sweating response was observed with the use of graduated compression stockings. |
| Goh et al., 2011 | 10 recreational runners, M, (29.0 ± 10.0 y) | To compare the effects of compression garments on running performance at ventilatory threshold one and at velocity at VO2_max_ in hot (32C) compared with cold (10C) ambient temperatures. | Rectal and skin temperature. Hydration status | Running commenced on the treadmill at a velocity that elicited the subject’s pre-determined ventilatory threshold one for 20 min, followed by a run to exhaustion at the subject’s individual VO_2max_ in 10C and 32C temperature. | During | Calf: 13.6 ± 3.4 mmHg  Thigh: 8.6 ± 1.9 mmHg | Lower body compression garments induced a 1.5℃ skin temperature increase at 10℃ but not 32℃ |
| Houghton et al., 2009 | 10 amateur field hockey, M,  (21.0 ± 2.0 y) | To investigate the effects of compression garments on thermoregulation in field hockey players. | Core and skin temperature | The Loughborough intermittent shuttle test | During | NS | Only skin temperature was significantly higher in compression than control.  Similar heart rate between trials. |
| Leoz-Abaurrea et al., 2015 | 13 untrained participants, M, (21 ± 6 y) | To investigate whether a heat dissipating upper body compression garment can mitigate thermoregulatory strain better than non-compression garments during cycling in hot (i.e., 40 degree) temperatures. | Weight loss, sweat rate, sweat retention in the garment, rectal temperature | Cycling at a fixed workload (~50% VO_2peak_) with 4 bouts of 14 minutes at 40 ºC with each bout separated with a minute active recovery | During | NS | Upper body compression garment impaired cardiorespiratory responses during recovery. Additionally, a significantly lower reduction in heart rate occurred during active recovery |
| Leoz-Abaurrea et al., 2016 | 10 recreational runners, M, (23.0 ± 3.0 y) | To analyse the physiological responses of heat dissipating upper body compression garments during a running performance test to exhaustion | Core temperature | 45-min run at 60% of the peak treadmill speed followed by a time to exhaustion run at 80% of the peak treadmill speed | During | *Bicep brachii*: 2.9 ± 1.5 mmHg, *Triceps*: 3.0 ± 1.0 mmHg, *Pectoralis major*: 2.0 ± 0.5 mmHg, *Latissimus dorsi*:1.4 ± 0.5 mmHg | Upper body compression garment impaired cardiorespiratory responses during exercise. |
| Leoz-Abaurrea et al., 2016 | 16 untrained participants, 4F and 12M, (21.3 ± 5.7 y) | To determine the effects of upper body compression garments on thermoregulatory responses during cycling in a controlled laboratory thermoneutral environment (~23°C). A secondary aim was to determine the cardiovascular and perceptual responses when wearing the garment. | Hydration status, body mass, sweat rate, core, skin and mean body temperature, Heat storage | cycling at a fixed workload (~50% VO_2peak_) with 4 bouts of 14 minutes at 40 ºC with each bout separated with a minute active recovery | During | NS | Wearing an upper body compression garment helped in lowering mean body temperature during the recovery process in a thermoneutral environment when compared to a similar control garment. |
| Leoz-Abaurrea et al., 2017 | 12 trained individuals, M, (66.0 ± 2.0 y) | To evaluate the effects of an upper body compression garment vs. a control garment on thermoregulatory responses in trained older adults in a temperate environment. | Sweat rate, Core, skin and mean body temperature, heat storage. | Cycling trial consisted of 4 bouts at a fixed load (50% peak power output) for 14 min, with each separated with a minute rest | During | Arm: ~1 - 3 mmHg of compression | Results showed that wearing an upper body compression garment led to a significantly higher temp-core and temp-body at the end of exercise. |
| Leoz-Abaurrea et al., 2017 | 20 recreational road cyclists, 4F and 16M, Compression group (21.4 ± 4.4 y), Control group: (19.9 ± 2.5 y) | To evaluate the effects of a heat dissipating upper body compression garment on thermoregulatory, cardiovascular, and perceptual responses during continuous cycling at a moderate intensity in a hot environment. | Weight loss, sweat rate, sweat retention in the garment, rectal temperature, body temperature | Cycling trial consisted of 30 minutes at a moderate intensity (~50% V̇O_2max_). | During | NS | The group of participants who wore the upper body compression garment finished the 30-minute exercise bout at 40ºC with significantly greater thermoregulatory and cardiovascular strain compared with participants who wore a control garment. |
| MacRae et al., 2012 | 12 recreationally trained cyclists, M, (26.0 ± 7.0 y) | To examine the effects of full-body compression garments on cardiovascular and thermoregulatory function during rest and exercise, and on exercise performance. | Skin and core temperature, skin wetness. | 60-min fixed-load cycling at ~65% VO_2max_ and a 6-km time trial. | During | At rest:  Correctly sized, full body compression garment, forearm: 13 ± 2 mmHg, thigh: 11 ± 2 mmHg,  calf: 15 ± 1 mmHg.  Oversized,  forearm: 9 ± 2 mmHg,  thigh: 8 ± 2 mmHg, calf: 13 ± 2 mmHg.  Exercise:  Correctly sized Full body compression garment  thigh: 10 - 16 mmHg,  calf: 11 - 18 mmHg. Oversized,  thigh: 8 - 13 mmHg,  calf: 9 - 14 mmHg | Full-body compression garments did not significantly augment stroke volume during rest or prolonged dynamic exercise. These garments caused increases in skin temperature and exercising cardiac output without affecting core temperature, stroke volume, arterial blood pressure |
| Ménétrier et al., 2015 | 15 endurance trained, M, (22.5 ± 0.7 y) | To assess the effect of compression stockings and contrast water therapy on muscle leg blood flow following a high-intensity interval training session. | Skin temperature. | Cycling, 9 x 5 min intervals (4 min at 50% of peak power output followed by 1 min at 80% of peak power output). | After | Thigh: 14 mmHg, Calf: 27 mmHg, Ankle: 15 mmHg | During immediate recovery of a high intensity exercise, compression garments result in higher femoral artery blood flow than passive recovery. |
| Priego Quesada et al., 2015 | 44 runners, 15F and 29M, (29.3 ± 5.8 y) | To analyse the effects of running in a moderate environment with and without graduated compression stockings on skin temperature in runners. | Skin temperature. | 20 min at 75% of their maximal aerobic speed and finished the running test with an established cool-down | During | Ankle: 20 – 25 mmHg, knee: 15–10 mmHg) | Running with graduated compression stockings produces a greater increase of skin temperature without modifying the athlete's heart rate. |
| Šambaher et al., 2016 | 15 active participants, 8F (22.3 ± 1.5 y) and 7M (24.8 ± 4.3 y) | To examine the effects of compression garments on neuromuscular performance, blood lactate, and skin temperature before and after fatigue | Skin temperature. | Drop jumps from 30cm | During | Graduated compression Ankle: 20 – 30 mmHg | Skin temperature was higher with ankle compression garments compared with control |
| Toolis & McGawley et al., 2020 | Seven senior biathletes from the Swedish national team, 3F & 4M, (25.1 ± 3.1 y) | To assess the effects of wearing upper- and lower-body compression garments on laboratory-based roller-skiing performance in elite biathletes, using ski durations and techniques simulating the demands of biathlon racing. | Thermal sensation and thermal comfort | Roller Ski time trial followed by a test of time to exhaustion | During | Biceps: 7.4 ± 2.2 mmHg, Triceps: 7.9 ± 2.2 mmHg, *Brachioradialis*: 13.1 ± 4.5 mmHg, *Rectus femoris*: 13.3 ± 2.3 mmHg, *Gastrocnemius*: 19.9 ± 5.9 mmHg | However, compression was shown to elicit a moderate effect of a lowered heart rate at rest, post warm-up, and post-time trial. |
| Venckunas et al., 2014 | 13 active participants, F, (25.1 ± 4.2 y) | To evaluate the effect of lower body compression garments on the cardiovascular function in response to a running session in a thermoneutral environment. | Rectal and skin temperature. | 4 km was covered in 30 min + 400m sprint | During | Thigh: ~17 mmHg, upper calf: ~19 mmHg | Except for the increased skin temperature of the undergarment region and venous emptying at 30 min post exercise, no differences were found both at rest and in response to running sessions. |

M = Male, F = Female, NS = Not specified, VO_2max_ = Maximal oxygen uptake.
