# Supplementary material for: Putting the Squeeze on Compression Garments: Current Evidence and Recommendations for Future Research: A Systematic Scoping Review
Source: Sports Med. 2021 Dec 6;52(5):1141–60. doi: 10.1007/s40279-021-01604-9 (PMC9023423; doi:10.1007/s40279-021-01604-9)
Supplement: Supplementary file 8 — Supplementary file8 (DOCX 96 kb) [file 40279_2021_1604_MOESM8_ESM.docx]

**Supplementary Table S8.** Details of studies and information relevant to perceptual outcomes.

| **Study** | **Cohort/ sample size (n), sex, age** | **Study purpose** | **Outcome Measures** | **Exercise Protocol** | **Compression worn during/after/both** | **Compression pressure – reported value or not stated** | **Key findings** |
| --- | --- | --- | --- | --- | --- | --- | --- |
| Ali et al., 2007 | Experiment 1: 14 participants, M, (22 ± 0.4 y)  Experiment 2: 14 healthy participants, M, (23 ± 0.5 y) | To examine the influence of wearing graduated compression stockings on several physiological and perceptual responses during and after exercise. | Experiment 1: RPE post multi-stage fitness test.  Experiment 2: MS before, post and 24 h post, RPE post exercise | Experiment 1: two multi-stage intermittent shuttle running tests with 1 h recovery between tests.  Experiment 2: continuous 10-km road run. | During | 18 – 22 mmHg | A reduction in muscle soreness 24 h after a bout of high-intensity continuous road-running while wearing the stockings. No effect on RPE |
| Ali et al., 2010 | 10 triathletes, 1F and 9M, (36.0 ± 10.0 y) | To examine the physiological and perceptual responses to wearing graduated compression stockings during fast-paced running | Changes in MS pre-run, immediately and 24 and 48 h post-run | 90% of 10 km personal best speed at 1% incline for 40 mins on treadmill | During | Low CG: 12 - 15 mmHg  High CG: 23 - 32 mmHg | Graduated compression stockings did not have an impact on MS in the days following exercise. |
| Ali et al., 2011 | 12 well-trained runners, 3F and 9M, (33 ± 10 y) | Examine the effects of wearing different grades of graduated compression garments on 10-km running performance | RPE, pleasure-displeasure, perceived activation, PCT, MP | 10km running time trial | During | Compression at the ankle and knee; low condition 15 and 12 mmHg; medium condition: 21 and 18mmHg; High 32 and 23 mmHg | Perceptions of comfort, tightness, and pain were most favourable during Low and Con trials. |
| Areces et al., 2015 | 34 marathon runners, 4F and 30M,  Control group: (42.7 ± 7.8y), Compression group: (41.2 ± 8.9 y) | Investigate the benefits obtained by wearing graduated compression stockings on running pace, prevention of muscle damage and maintenance of muscle performance during a real marathon race | RPE, MP | Marathon race | During | The highest pressure was at the foot and the malleolus and it decreased proximally, from 25 mmHg to 20 mmHg | Improved subjective muscle pain 24-h after the marathon |
| Argus et al., 2013 | 11 highly trained cyclists, M, (31 ± 6 y) | To evaluate the use of 3 recovery strategies compared with a passive control on repeated sprint-cycling performance in highly trained cyclists | TQR, belief in recovery interventions | 3 maximal 30 s sprint separated by 20 min of recovery with compression garment | Between maximal sprints | A pressure gradient of 27 ± 6 mmHg at the lower calf and 18 ± 2 mmHg at the upper thigh | No effect |
| Armstrong et al., 2015 | 33 moderately trained runners, 10F and 23M, (38.5 ± 7.2 y) | To determine if lower limb compression garments influence functional recovery from distance running | RPE | Marathon race | During | Below knee compression socks with ankle: 30-40mmHg; calf: 21-28mmHg | No effect on RPE |
| Atkins et al., 2020 | 30 recreational basketball athletes,  M, (22.5 ± 4.1 y) | To evaluate the effect of wearing lower-body compression garments following basketball-specific exercise on perceptual ratings of recovery and physical performance | MS, PF | The Basketball Exercise Simulation Test | Post-exercise for 15 hours | Ankle: 7.5 ± 3 mmHg  Calf: 10 ± 2.5 mmHg  Thigh: 8 ± 2 mmHg | Compression garments aided PF and MS with large, significant improvements compared to the control group. |
| Bahnert et al., 2012 | 45 AFL players, M, (23.3 ± 4.2 y) | To investigate the associations between post-game recovery modalities chosen by Australian Football League players and their subsequent physical and perceptual recovery, and game performances throughout a season. | PR, PF, MS | AFL match | After | NS | Perceptual recovery among players was enhanced through the selection of specific combinations of recovery protocols post game. However, no links were found between recovery protocols and physical or game performance measures. |
| Ballman et al., 2019 | 12 basketball players, M, (20.3 ± 1.37 y) | To examine the effects of wearing lower body compression garments on anaerobic exercise performance | RPE | 2 x 30 second repeated WAnT | During | Ankle: 15–20 mmHg  Thigh: 6–10 mmHg at the thigh | No effect |
| Barwood et al., 2013 | 8 physically active, M, (21 ± 2 y) | To establish the thermal and performance effects of wearing graduated compression garments in a hot environment in contrast to control and sham treatment conditions | RPE, TC and TS every 5min | 15 minutes running at 10-12km/h followed by a 5 km time trial | During | Calf:  Compression: 20 ± 3 mmHg;  Sham: 17 ± 4 mmHg  Thigh  Compression: 11 ± 2 mmHg  Sham: 10 ± 2 mmHg | No effect on perceptual measures |
| Beaven et al., 2013 | 16 professional rugby players, M, (25 ± 3 y) | To assess the effectiveness of compression garments and an electrostimulation device at assisting recovery of professional rugby players during a preseason training period. | RPE | Preseason training | During | NS | The combination of an electrostimulation device with a compression garment was effective at eliciting positive psychometric benefits in professional male rugby athletes over a preseason training period. |
| Bieuzen et al., 2014 | 11 highly trained runners, M, (34.7 ± 9.8 y) | To examine the effect of wearing compression stockings on indices of EIMD during trail-running. Compression stockings were worn either during or after a trail run performed at competition pace by experienced off-road runners | Immediate and delayed-onset MS | The simulated trail race consisted of 3 laps of 5.2 km (total distance: 15.6 km) in mountainous terrain | During | Elastic CS during running (pressure of 25 mmHg). For the CS recovery condition, an elastic CS was used (pressure of 20 mmHg) | Perceived immediate MS likely lower |
| Born et al., 2014 | 10 elite German ice speed skaters, M, (23 ± 7 y) | To investigate whether the application of compression improves muscle oxygenation and blood volume, ratings of perceived exertion; blood lactate concentration; and, 3000-m time. | RPE for calf, thigh and whole body | 3000-m race simulation | During | 20.3 ± 2.3 mmHg at the thigh and 24.4 ± 3.1 mmHg at the calf | No effect on RPE. |
| Born et al., 2014 | Sub-study 1:  12 track and team sport athletes, F, (25.0±3.0y); Sub-study 2: 12 track and team sport athletes, F,  (23 ± 2 y) | There were 2 aims; 1) To assess the effects of compression garments with silicone stripes (which mimic kinesio taping) on repeated sprint performance; 2) to identify the physiological, biomechanical, and perceptual effects of compression garments with silicone stripes | RPE of the whole body, upper legs, and lower legs | 30 x 30-m sprints (one sprint per minute) | During | ~18 – 20 mmHg across the entire lower body | No effect |
| Bringard et al., 2005 | 6 trained runners, M, (31.2 ± 5.4 y) | To examine the effect of wearing compression tights compared to wearing shorts and classic tights on aerobic energy cost of running at various submaximal running intensities | RPE | Continuous incremental exercise test to voluntary exhaustion | During | NS | RPE was not significantly different between conditions, |
| Broatch et al., 2019 | 12 elite Australian volleyball athletes, F, (25 ± 2 y) | To determine the effects of wearing compression socks during long-haul travel on sports-specific performance, physiological, and haematological alterations in elite female volleyball athletes | PF, MS | Air travel | Worn during travel | Calf: 23 ± 11 mmHg  Ankle: between 19 and 22 (±8) mmHg at the ankle | Sports compression socks improved subjective ratings of fatigue and muscle soreness following travel. |
| Brophy-Williams et al., 2016 | 12 well trained runners, M, (30.5 ± 8.1 y) | to assess the effect of wearing compression socks during a one-hour recovery period following a 5km running time trial on performance in a subsequent 5km time trial | MS, PF, TQR | A 5km TT, then a one-hour recovery intermission before a second warm-up and 5km TT. | Between time trials | Calf: 23 ± 11mmHg  Upper ankle: 22 ± 8 mmHg  Lower ankle: 19 ± 8 mmHg | Participant belief in the product played a large role in determining whether a benefit occurred. Muscle soreness was reduced with compression garment |
| Brophy-Williams et al., 2018 | 12 runners, M, (30.5 ±8.1y) | Assess the effect of wearing compression socks during a 5km running time trial on physiological, perceptual and performance-based parameters, including subsequent performance. | RPE, MS, PF, TQR | A5 km time trial, a one-hour recovery period, then a repeat of the warm-up and 5 km time trial. | During the first warm up and time trial. | Calf: 37 ± 4 mmHg  Upper ankle: 31 ± 4 mmHg  Lower ankle: 23 ± 4 mmHg | No effect |
| Burden and Glaister 2012 | 10 well-trained triathletes and cyclists, M, (34.6 ± 6.8 y) | To investigate the effects of both ionized and nonionized compression tights on the physiological and performance responses to, and short-term recovery from, sprint and endurance cycling | RPE at end of each stage | 8 x 3–minute stages on a cycle ergometer,  30 seconds at 150% of the power output required to elicit VO_2max_. Subjects then recovered for 3 minutes at 40% power of VO_2max_ before performing a maximal 30-second Wingate anaerobic test | During | Control: Mid-thigh Anterior: 8mmHg, Posterior: 6 mmHg. Mid-shank Anterior: 11 mmHg, Posterior: 8 mmHg  Nonionized and ionized: Mid-thigh Anterior: 15 mmHg, Posterior: 11 mmHg. Mid-shank Anterior: 21 mmHg, Posterior: 16 mmHg | Ionized compression tights had no effect on physiological parameters or cycling performance of any type. |
| Carling et al., 1995 | 23 participants,  16F and 7M, (26 ± 4 y) | Evaluate the effect of compression on DOMS and the accompanying manifestations of soreness, swelling, range of motion, and isokinetic strength for a period of 4 days following induction of DOMS | MS | 70 eccentric contractions of the elbow flexor muscles, dynamometer test (120˚/s through 120˚ of motion) | After | Sleeve pressure: 17 mmHg | No significant differences were present for either group. |
| Chatard et al., 2004 | 12 trained cyclists, M, (63 ± 3 y) | To determine whether compression stockings affect performance recovery and leg pain following maximal exercise. | RPE, leg MP | All out five-minute cycloergometer followed by 80-min rest and then another all out five minutes | For 80 minutes between maximal attempts | Ankle: 44 hPa  Calf: 24 hPa  Mid-thigh: 17 hPa | No change in RPE, however reduced leg MP |
| Choi et al., 2019 | 38 baseball players, M, (19.6 ± 0.2 y) | To examine the effects of wearing DPV576-C garments at night on the physical and psychological stress of Japanese male baseball players induced by strenuous training over a two-week period | POMS questionnaire | Strenuous training over a two-week period | Participants were asked to wear the garments before bedtime and for at least 10 h (9:00 pm–07:00 am) every night for two weeks | NS | Nanodiamond- and nanoplatinum-coated fibres (DPV576-C) in compression garments worn at night significantly suppressed an increase in total mood disturbance levels |
| Dascombe et al., 2013 | Seven elite flat-water kayakers, 2F (25.0 ± 4.2 y) and 5M (21.8 ± 2.8 y) | To determine the effects of wearing upper body compression garments on performance during simulated flat-water kayaking and physiological responses during simulated kayaking | RPE at end of each increment | Participants completed a six-step incremental test and a subsequent 4min performance test on a kayak ergometer | During | NS | No effect |
| Davies et al., 2009 | Seven female netball players and four male basketball players, 7F (19.7 ± 0.5 y) and 4M (26.3 ± 5.1 y) | To investigate whether wearing compression tights for 48 hours following plyometric exercise would attenuate muscle damage markers and muscle soreness, and sprinting and jumping performance | MS | 5 sets of 20 drop jumps from a platform 60 cm high followed immediately by a maximal upward jump, with a 2-minute rest period between sets | For 48 hours afterwards | The compression tights applied a graduated pressure of approximately 15 mmHg from the lower to the upper legs | Reduced perceived muscle soreness |
| Driller and Halson 2013 | 12 highly trained male cyclists, M, (30 ± 6 y) | To investigate the effect of wearing lower body compression garments on performance during a 30-minute endurance cycling test. A further aim of the study was to determine various physiological and perceptual responses when wearing compression garments during the cycle test. | MS | 15 minutes at a workload equal to 70% PPO, followed immediately by a 15-minute time trial. | During | ~18 mmHg at the medial malleolus decreasing to ~10 mmHg at the gluteus maximus | No effect |
| Duffield and Portus, 2015 | 10 club cricket players, M (22.1 ± 1.1 y) | To compare the effects of three different types of full-body compression garments and a control condition on performance in intermittent, repeat-sprint and throwing performance in cricket players. | RPE every 10min. MS of the arm and leg before and 24h post exercise. | 30 min repeat-sprint exercise protocol comprising 20 m sprints every minute, separated by submaximal exercise. Throwing tests included a pre-exercise and a post-exercise maximal distance test and accuracy throwing tests. | During and 24 hours after | NS | A significant difference was observed between compression garments and control conditions for the rating of muscle soreness of the arm and legs 24 h after exercise |
| Duffield et al., 2008 | 14 rugby players, M, (19 ± 1 y) | To determine whether compression garments improve intermittent-sprint performance and aid performance or self-reported (perceptual) recovery from high-intensity efforts on consecutive days | MS | 4 x 15-min quarters of a simulated team game (exercise circuit) repeated across two days | During the simulated team games and for ~15 hours afterwards | NS | Reduced MS after wearing compression |
| Duffield et al., 2014 | 8 professional tennis players, M, (20.9 ± 3.6 y) | To investigate the effects of combining cold water immersion, compression garments, and sleep-hygiene recommendations on physical, physiological, and perceptual recovery after 2-a-day on-court training and match-play sessions | MS | Each respective on-court session involved 90 minutes of coach-led drills (including a 30-min warm-up) and 90 minutes of competitive match play | Between drill and match play (~3 hours) and for 4 hours match play | NS | Post-match cold water immersion and compression garments reduced perceived soreness |
| Ehrstrom et al., 2018 | 13 trail runners, M, (38.6 ± 5.7 y) | To examine whether wearing high-pressure compression garments during a 40-min treadmill downhill run on acute and delayed neuromuscular responses and running economy | MS | 40-min downhill running at –8.5 deg decline | During | 15 – 20 mmHg for quadriceps and calves | Reduced muscle soreness |
| Faulkner et al., 2013 | 11 trained runners, M (23.7 ± 5.7 y) | To examine the effects of lower-limb compression on 400-m run performance and physiological and perceptual indicators of intensity and performance | RPE at each 100m split. MS, PCT. Feeling scale and arousal scale monitored pre/during/post | 400m sprint | During | Long garment: 2.0 - 13.2 mmHg  Short length garment: 3.7-20.7 mmHg | Significantly lower RPE when wearing compression |
| Ferguson et al., 2014 | 21 healthy participants, M,  (21 ± 1 y) | To examine the effects of neuromuscular electrical stimulation, compared to graduated compression socks on muscle soreness, strength, and markers of muscle damage and inflammation following intense intermittent exercise | MS | Two 45-min sections of continuous intermittent exercise separated by a 15-min rest period.  3 × 20 metres at walking pace, 1 × 20 metre maximal sprint, 4 s recovery, 3 × 20 metres at a running speed corresponding to 75 % VO_2max_, 3 × 20 metres at a running speed corresponding to 100 % VO_2max_ | At least 12 h post the 1 h testing point | Ankle: 40 mmHg  Calf: 20 mmHg | At 24 h post exercise, MS was lower than control in the compression trial. |
| French et al., 2008 | 26 participants, M, (24.12 ± 3.2 y) | To evaluate contrast bathing and contrast garments as regeneration strategies after EIMD | MS | 6 x 10 squats with 100% of body mass + 5 second eccentric repetition with the participants 1RM | For 12 hours after exercise | 12 mmHg at the calf and 10 mmHg at the thigh | No effect |
| Geldenhuys et al., 2019 | 41 marathon runners,  Experimental group, 6F and 14M, (34 ± 4.8 y)  Control group,  6F and 15M, (34 ± 6.4 y) | To determine the impact of below-knee CGs on lower leg EIMD and performance in runners before, during, and after an ultramarathon road race | MP | 56-km ultramarathon | During | NS | Higher pain scores |
| Gimenes et al., 2019 | 20 football players, Sex NS, 10 compression (18.3 ± 0.5 y) and 10 control (18.5 ± 0.5 y) | the effects of using compression stockings on the match-based physical performance indicators, HR responses, and perceptual measurements in under-20 soccer players during 2 matches separated by 72 hours | RPE during matches, perceived soreness before and after | Two football matches separated by 72 hours | During | Estimated between 20-30 mmHg | No effect RPE but a smaller increase in perceived soreness following the second match |
| Goh et al., 2011 | 10 recreational runners, M, (29.0 ± 10.0 y) | To compare the effects of compression garments on running performance at ventilatory threshold one and at velocity at VO2_max_ in hot (32C) compared with cold (10C) ambient temperatures | RPE | Running commenced on the treadmill at a velocity that elicited the subject’s pre-determined VT1 for 20 min, followed by a run to exhaustion at the subject’s individual VO_2max_ in 10C and 32C temperature. | During | Calf: 13.6 ± 3.4 mmHg  Thigh: 8.6 ± 1.9 mmHg | RPE was significantly lower at both 10 and 20 min during the run at VT1 in 32C when wearing compression garments as compared to the control clothing |
| Goto and Morishima, 2014 | 9 participants, M, (21.0 ± 0.4 y) | To investigate the effects of wearing a compression garments for 24 h on the changes in muscular strength and blood parameters over time after resistance exercise. | MS | Six exercises for the upper body and three for the lower body muscles. Each exercise set comprised 10 repetitions involving five sets for bilateral leg press and bilateral knee extension and three sets for the remaining seven exercises | Worn for 24 hours after resistance training | NS | Muscle soreness and fatigue were reduced by wearing the CG during the post-exercise period. |
| Goto et al., 2017 | 11 participants, M, (22.7 ± 0.9 y) | To determine the effect of compression garments during post-exercise periods after two repeated bouts of exercise on exercise performance, muscle damage, and inflammatory responses | PF, MS | Repeated sprint cycling and resistance exercise | The subjects wore the prescribed garments throughout the whole recovery period (4 h after experiment 1 and approximately 18 h after experiment 2), except during two repeated bouts of exercise (60 min for each exercise). | 11.5 ± 0.6 hPa for the thigh and 17.6 ± 1.8 hPa for the calf | Reduced muscle soreness for upper body muscles |
| Govus et al., 2018 | 32 cross country national and junior level skiers, Seniors  9F (23.2 ± 2.6 y) and  12M (25.2 ± 3.6 y),  Juniors  5F (18.2 ± 0.8 y) and  6M (18.0 ± 0.6 y) | To determine whether compression garments and neuromuscular electrical stimulation accelerated the recovery of blood biomarkers of muscle damage, countermovement jump height and perceived muscle pain before and 8, 20, 44 and 68 h after a cross-country sprint skiing competition in a cohort of elite senior and junior cross-country skiers. | MP measured at baseline and 8, 20, 44, and 68 h post sprint skiing | Cross-country sprint skiing competition | For ∼17 h following the post-competition period. | Lower-body compression, ankle: 14.6 ± 0.1 mmHg, *achilles tendon*: 13.6 ± 1.4 mmHg, calf: 13.7 ± 1.3 mmHg, *tibial tuberosity*: 7.6 ± 1.1 mmHg, *patella*: 8.3 ± 2.3 mmHg, mid-thigh: 5.3 ± 1.1 mmHg and 5 cm below crotch: 4.2 ± 2.8 mmHg | No effect |
| Hamlin et al., 2012 | 22 rugby union players, M, (20.1 ± 2.1 y) | To determine the effects of wearing either a compression garment or placebo garment over a 24-hour recovery period on subsequent physiological and performance measures | MS before, after and 24 hours post rugby circuit, PF | A series of exercise circuits designed to simulate a game of rugby | After | Compression: *Sphyrion*: 8.6 ± 2.6 mmHg, mid-calf: 13.4 ± 2.0 mmHg, and *mid*-*trochanterion*: and 9.0 ± 2.2 mmHg  Control: *Sphyrion*: 2.6 ± 1.2 mmHg, mid-calf: 5.0 ± 1.5 mmHg, and *mid*-*trochanterion*: 3.5 ± 0.9 mmHg | Reduced MS and PF |
| Heiss et al., 2018 | 15 participants, 7F and 8M (25 ± 6 y) | To investigate the influence of commercially available sport compression garments on the development of exercise-induced intramuscular oedema | MS | All participants performed 5 sets of 30 repetitions of calf raises with 25% of their body weight during the exercise and rested 10 sec between each set. | The compression sock was worn continuously for 60 h after eccentric exercise and was removed for the first time for follow-up examination | NS | No effect |
| Hettchen et al., 2019 | 19 handball players, M, (31.3 ± 7.7 y) | To determine the effect of compression tights on relevant parameters of recovery | PEPS | Two sets of 8-10 repetitions of lunges, unilateral calf raises, and squats were prescribed. Exercise to failure per exercise in the range of 8-10 reps. 60 seconds recovery between exercises | Compression was applied initially for 24 h and then 12 h intermitted by 12 h of non-use for a total of 96 h | 19.0 - 26.2 mmHg for the onset of the calf muscle; 16.3 - 23.5 mmHg for the highest calf circumference; 9.9 - 18.1 mmHg two fingerbreadths beneath the *fossa popliteal*; 7.7 - 14.3 mmHg at the mid-knee; 9.9-13.9 at mid-thigh and 8.0 - 12.2 mmHg for the region two fingerbreadths beneath the crotch | Improved PEPS |
| Hill et al., 2014 | 24 recreational marathon runners, 7F and 17M, Compression group (47.7 ± 10.8 y),  Sham ultrasound (41.1 ± 10.5 y) | To investigate the effects of wearing a commercially available, lower limb, compression garment on the recovery of strength, soreness and indices of muscle damage following a marathon run. | RPE, MS | Marathon run | For 72 hours after exercise | Between 9.9 - 24.4 mmHg | The compression garment group experienced less muscle soreness 24 h post marathon when compared to the sham group. |
| Hill et al., 2017 | 45 recreationally active participants, 19F and 26M, Low pressure group  (29.2 ± 4.7 y),  High group (32.7 ± 7.8 y),  Sham group (28.3 ± 4.1 y) | To assess whether garments exerting a higher degree of pressure are more effective in facilitating recovery compared to garments exerting a lower pressure | Global lower limb and quadriceps MS measured 1, 24, 48 and 72 h post muscle damaging protocol | The muscle damaging protocol consisted of 100 drop jumps from a 0.6 m platform. Participants performed 5 sets of 20 drop jumps, with 10 seconds between each jump and a 2 min rest period between sets | For 72 hours post exercise | Low condition  Thigh: 8.1 ± 1.3 mmHg  Calf: 14.8 ± 2.1 mmHg.  High condition  Thigh: 14.8 ± 2.2 mmHg  Calf: 24.3 ± 3.7 mmHg | No effect |
| Hintzy et al., Ahead of print | 12 participants, M, (25.3 ± 3.6 y) | Examine the effects of different levels of thigh compression in shorts on both vibration and muscle activity of the thigh during cycling with superimposed vibrations. | RPE | Four sets of 18-minute cycling test | During | 0, 2, 6 and 15-mmHgdepending on condition. | No effect |
| Houghton et al., 2007 | 12 trained amateur field hockey players,  M, (21 [19-23] y) | To investigate the effects of compression garments on thermoregulation in field hockey players. | RPE | The Loughborough intermittent shuttle test | During | NS | No difference in RPE between trials |
| Jakeman et al., 2010 | 32 physically active participants, F, (21.4 ± 1.7 y) | To determine whether a combined treatment involving sports massage and compression immediately after damaging exercise was an effective strategy to manage the symptoms of EIMD induced by strenuous plyometric exercise | MS | 10 x 10 plyometric drop jumps from a 0.6-m box. One minute rest between sets | For 12 hours post-exercise | Average compression of 17.3 mmHg at the calf and 14.9 mmHg at the thigh | Improved perceived MS |
| Jakeman et al., 2010 | 17 physically active participants, F, (21.4 ± 1.7 y) | To investigate the efficacy of complete lower limb compression clothing on recovery from the symptoms of EIMD following strenuous plyometric activity | MS | 10 x 10 plyometric drop jumps from a 0.6-m box. One minute rest between sets | For 12 hours post-exercise | Average compression of 17.3 mmHg at the calf and 14.9 mmHg at the thigh | Compression can diminish perceptions of soreness following damaging exercise. |
| Kerhervé et al., 2017 | 14 participants, M, (21.7 ± 3.0 y) | To determine if wearing calf compression sleeves during a prolonged running exercise affected local muscle tissue oxygenation, running pattern, muscle power capability, performance, and subjective perception of muscle fatigue, pain and soreness. | PF and pain in the calf, thigh, and Achilles, MS | 24 km run | During | 23 ± 2 mmHg | Wearing compression sleeves improved the perception of pain in the Achilles’ tendon |
| Kim et al., 2017 | 16 participants, M, 8 control, (23.13 ± 3.76 y), 8 compression, (24.25 ± 1.28 y) | To investigate wearing compression garments after eccentric exercise using elbow flexor and effects on DOMS and inflammatory response | MS | Each eccentric muscle contraction was performed for 3 sec. Each participant completed two sets, performing 25 reps per set with a 5-min rest period between sets | 24 hours after exercise | 5–10 mmHg | Compression garments reduced perceived muscle soreness |
| Kraemer et al., 1998 | 20 athletic participants, 10F (21.3 ± 2.3 y) and 10M (22.3 ± 4.8 y)  20 non-athletic controls, 10F (20.3 ± 2.4 y) and 10M (21.3 ± 2.3 y) | The purpose of this study was to determine whether compression shorts affected vertical jump performance after different fatigue tasks. | Perceived Performance | Endurance Fatigue: 30 min run at 70% HR_max_  Strength Fatigue: 4 x 10 leg press (10RM)  Power Fatigue: 10 x 10 maximal effort jumps | During | NS | The compressive garment significantly enhanced mean power output in the jump test both before and after different fatigue tasks. |
| Kraemer et al., 2001 | 20 non-strength-trained participants, F,  Compression sleeve group (21.3 ± 2.9 y),  Control group,  (21.1 ± 3.3 y) | To investigate whether constant compression via the use of a compressive arm sleeve would reduce the severity and duration of soreness associated with DOMS. | Global soreness, soreness through ROM, soreness with palpation, perception of difficulty through daily activities | Dynamometer (60°/s). 2 sets of 50 repetitions with 3 minutes rest between sets. Every fourth repetition, the subject performed a maximal concentric contraction with an isometric hold followed by an eccentric contraction | After | 10 mmHg | Decreased pain at rest and during palpation of the exercised muscle. |
| Kraemer et al., 2010 | 20 resistance trained subjects, 9F (23.1 ± 2.2 y) and 11M (23.0 ± 2.9 y) | To evaluate the influence of a whole-body compression garment on recovery from a typical heavy resistance training workout | MS | 3 sets at 8-10RM of back squats, bench press, stationary lunge, bent-over row, Romanian dead lift, biceps curl, sit up, high pull from hang. | For 24 hours after exercise | NS | No effect |
| Kraemer et al., 2016 | 15 healthy non-strength-trained, M, Compression sleeves group  (22.3 ± 2.9 y),  Control group  (22.1 ± 3.3 y) | To determine whether a compression sleeve worn immediately after maximal eccentric exercise enhances recovery | MS before exercise and for 3 days | Dynamometer (60°/s). 2 sets of 50 repetitions with 3 minutes rest between sets. Every fourth repetition, the subject performed a maximal concentric contraction with an isometric hold followed by an eccentric contraction | After | NS | Compression sleeves decreased subjects’ perception of soreness |
| Leicht et al., 2020 | 30 active participants, 15F and 15M, (19.8 ± 1.9 y) | To examine the impact of lower body compression garments on cardiac autonomic control of heart rate prior to, during and following submaximal exercise. | RPE | Cycling at a moderate intensity equivalent to 70% age-predicted maximum heart rate | During and after | NS | No effect |
| Leoz-Abaurrea et al 2015 | 13 untrained participants, M, (21 ± 6 y) | To investigate whether a heat dissipating upper body compression garment can mitigate thermoregulatory strain better than non-compression garments during cycling in hot (i.e., 40 degree) temperatures. | RPE, thermal sensation | Cycling at a fixed workload (~50% VO_2peak_) with 4 bouts of 14 minutes at 40 ºC with each bout separated with a minute active recovery | During | NS | The group of participants who wore the upper body compression garment had an increased thermal sensation, with no change in RPE |
| Leoz-Abaurrea et al 2017 | 20 recreational road cyclists, 4F and 16M, Compression group (21.4 ± 4.4 y), Control group (19.9 ± 2.5 y) | To evaluate the effects of a heat dissipating upper body compression garment on thermoregulatory, cardiovascular, and perceptual responses during continuous cycling at a moderate intensity in a hot environment. | Thermal sensation, RPE | Cycling trial consisted of 30 minutes at a moderate intensity (~50% V̇O_2max_). | During | NS | The group of participants who wore the upper body compression garment had an increased thermal sensation, with no change in RPE |
| Li et al., 2011 | 2 healthy college students, M, (Subject 1, 26y; Subject 2, 27y) | To investigate the effect of tight knee-high gradient compression socks sportswear on lower limb muscle activity during running. | RPE | Run at 10-15 km/h until exhaustion | During | 27 hPa at the ankle | Wearing graduated compression socks during running seemed to help to alleviate muscle fatigue |
| Lucas-Cuevas et al., 2015 | 40 recreational runners, 20F and 20M, (28.4 ± 5.9 y) | To analyse the effect of 3 weeks of training with graduated compressive stockings and non-compressive stockings and muscle fatigue on stride kinematics, impact acceleration and perception of comfort during running | RPE, PC | After 3 weeks of training testing occurred during 30 min running at 80 % of the individual’s MAS | During training and testing | Ankle: 24 mmHg  Calf: 21 mmHg | No effect |
| Lucas-Cuevas et al., 2017 | 36 runners, 15F (29.17 ± 3.8 y) and 21M (28.14 ± 4.46 y) | To analyse the influence of graduated compression stockings on the perception of comfort and muscle activation of the lower leg during high intensity running | PC, PF | 20-min run at 75% of their MAS at 1% slope on a treadmill | During | 24 mmHg at the ankle and 21 mmHg at the calf | No difference in comfort or fatigue between conditions |
| MacRae et al., 2012 | 12 recreationally trained cyclists, M, (26 ± 7 y) | To examine the effects of full-body compression garments on cardiovascular and thermoregulatory function during rest and exercise, and on exercise performance | TS, RPE | 60-min fixed-load cycling at ~65% VO_2max_ and a 6-km time trial. | During | Correctly-sized group: 11–15 mmHg  Over-sized group: 8–13 mmHg | No difference when wearing compression |
| Marqués-Jiménez et al., 2017 | 18 semi-professional football players, M, (25.2 ± 3.0 y) | To evaluate the influence of wearing different types of compression garments during matches and recovery after a friendly soccer match | MS measured after halves, 24, 48 and 72 h post-match | Soccer match | Each participant in the experimental condition played the match wearing one type of graduated compression garment and kept wearing them 7 h/day during 3 days post-match | Compression stockings  Ankle: 20 – 25 mmHg  Calf: 15 – 20 mmHg  Compression tights  Calf: 25 – 30 mmHg  Thigh:15 – 20 mmHg  Compression shorts:  Thigh: 15 – 20 mmHg | Reduced MS |
| Marqués-Jiménez et al., 2017 | 18 semi-professional football players, M, (24.7 ± 4.07 y) | To evaluate the influence of wearing different types of compression garments during matches and recovery after a friendly soccer match | RPE (half time, post-match, 24, 48 and 72 h post-match), and PR using TQR scale (24, 48, and 72 h post-match). | Soccer match | Each participant in the experimental condition played the match wearing one type of graduated compression garment and kept wearing them 7 hours per day for 3 days post-match (players put them on each day after the testing session). | Compression stockings  Ankle: 20 – 25 mmHg  Calf: 15 – 20 mmHg  Compression tights  Calf: 25 – 30 mmHg  Thigh:15 – 20 mmHg  Compression shorts:  Thigh: 15 – 20 mmHg | No effect |
| Martínez Navarro et al., 2020 | 32 recreational ultra-endurance athletes, 13F and 19M, (41 ± 6 y) | To analyse the effect on DOMS, muscle damage, inflammatory response, and renal function of wearing a full-body compression garment for 24 h immediately after a 107-km ultra-trail | Pre-, and 24hr-post: DOMS | 107km Ultra trail race | Worn for 24 h post | 10 – 15 mmHg | Compression may be recommended as a recovery method to reduce muscle soreness |
| McDonnell et al., 2018 | 59 participants, 20F and 39M, (18 – 60 y) | To assess efficacy of two types of compression strategies (knee high graduated compression socks and knee high compression socks) following exercise on functional and perceived recovery | MP | Hike Trial and Trail Run | worn during the daytime for 4 days, including the day of the DOMS-inducing exercise | Ankles: 21 ± 0.7 mmHg, calf: 13.2 ± 1.9 mmHg; Uniform compression socks: ankle: 20.5 ± 2.2 mmHg, calf: 21.0 ± 2.4 mmHg | Compression socks mitigated the perception of calf muscle pain, with uniform compression providing more benefit compared to graduated socks. No differences between the uniform and graduated socks were observed in the calf exercise trial. |
| Ménétrier et al., 2011 | 14 moderately trained endurance participants, M, (21.9 ± 0.7 y) | To test whether calf compression sleeves influence RPE | RPE | 15 min at rest, 30 min at 60 % maximal aerobic velocity, 15 min of recovery, a running time to exhaustion at 100 % maximal aerobic velocity | Before, during and after | Medial ankle: 15 mmHg  *Gastrocnemius*: 27 mmHg | No change in RPE |
| Miyamoto and Kawakami 2015 | 15 healthy participants, M, (25.2 ± 2.6 y) | To examine the effect of pressure profiles of compression stockings on muscle fatigue level of the lower leg muscles induced by running exercise, and to test the pressure profiles against the development of muscle fatigue. | RPE at 0, 10, 20, 30 min of 12km/hr run | Running on a treadmill set at 0- inclination for 34.5 min including 4.5-min warm-up, 1.5 min at 6 km, 1.5 min at 8 km, 1.5 min at 10 km, and 30 min at 12 km | During | Graduated low pressure compression condition, *gastrocnemius*: 14mmHg and ankle:18 mmHg  Graduated high pressure compression condition, *gastrocnemius*: 21 mmHg and ankle: 27 mmHg  Uniform pressure distribution condition, *gastrocnemius* = 21 mmHg; Ankle =21 mmHg  Localized pressure at the *gastrocnemius* region condition, *gastrocnemius*: 21 mmHg and ankle: 10 mmHg | No effect on RPE |
| Miyamoto et al., 2014 | Two groups of 11 participants, M, Experiment 1 (25.6 ± 3.7 y), Experiment 2 (27.0 ± 1.8 y) | To examine the effect of pressure intensity of elastic compression short-tights on the metabolic state of thigh muscles during submaximal running | RPE | exercise on a treadmill set at 0- inclination for 34.5 min including 4.5-min warm-up running, 1.5 min at 6 km, 1.5 min at 8 km, 1.5 min at 10 km, and 30 min at 12 km | During | Experiment 1, two compression short-tights Low group: 8 mmHg  High group: 15 mmHg  Mid-high: 20 mmHg  High group: 25 mmHg | Reduced RPE when wearing compression is Exp 1 |
| Mizuno et al., 2016 | 18 participants, M, (21.9 ± 0.6 y) | To determine the effects of wearing a lower body compression garment for 24 h following running (either downhill or level) in terms of recovery of exercise performance, muscle damage, inflammatory markers in the blood, and subjective muscle soreness and fatigue. | MS, PF | 30 minutes of downhill running | 24 hours post exercise | Compression group: thigh:11.5 ± 0.6 hPa, calf:17.6 ± 1.8 hPa at the calf.  Control group: Thigh: 7.1 ± 1.3 hPa, calf: 11.5 ± 2.1 hPa | No effect |
| Mizuno et al., 2017 | 30 physically active participants  Compression thigh group, 10M,  (21.3 ± 0.4 y)    Compression sock group 10M, (21.6 ± 0.8 y)  Control group, 10M, (22.9 ± 0.7 y) | Examine the effects of the body coverage area of compression garments on the exercise performances and muscle damage during prolonged running | MS, PF, RPE | 120min of uphill running at 55% of VO_2max_ | During | Thigh compression group:  14.7 ± 0.6 mmHg  Calf compression group: 17.4 ± 0.5 mmHg  Control group: Thigh: 3.0 ± 0.3 mmHg and calf: 1.8 ± 0.2 mmHg | No significant effects of the body coverage area of the compression garments on subjective ratings. |
| Mizuno et al., 2017 | 8 participants, M, (23.4 ± 2.4 y) | To investigate the effect of wearing lower body compression garments exerting different pressure levels during prolonged running on exercise-induced muscle damage and the inflammatory response. | RPE (legs) | 120 min of uphill running at 60% of VO_2max_ | During | High pressure garment: Thigh: 26.9 ± 3.3 mmHg; Calf: 29.2 ± 3.8 mmHg  Medium pressure garment: Thigh: 16.1 ± 2.0 mmHg; Calf: 17.9 ± 3.5 mmHg  Control garment: Thigh: 4.4 ± 1.2 mmHg; Calf: 3.0 ± 1.6 mmHg | No effect |
| Montgomery et al., 2008 | 29 basketball players, M, (19.1 ± 2.1 y) | To (1) investigate the time course of muscle damage markers and inflammatory cytokines during basketball tournament play and (2) assess whether cold water immersion and compression recovery strategies ameliorate any post-game increases of these biomarkers, compared with traditional refuelling and stretching routines | MS | 3-day mini-tournament involving one full 48 min game per day | For ~18 hours post-game | ~18 mmHg | The application of compression garments appears to have little advantage in enhancing clearance of muscle damage biomarkers over consecutive days |
| Montgomery et al., 2008 | 29 basketball players, M, (19.1 ± 2.1 y) | To evaluate the effectiveness of recovery strategies on physical performance during a 3-day tournament style basketball competition | PF, MS | 3-day mini-tournament involving one full 48-min game per day | For ~18 hours post-game | ~18 mmHg | Compression reduced muscle soreness and perceived fatigue |
| Okamoto et al., 2012 | 10 healthy participants, M (29.8 ± 5.9 y) | Investigate the acute effect of brisk walking with and without graduated compression garments on vascular endothelial function and oxidative stress | RPE | Walking on a motor-driven treadmill at 5.9–6.7 km/h for 30 min at an intensity of ~60% of heart rate reserve | During | Ankle: 25mmHg and calf: 17mmHg | No effect of wearing compression |
| Pavin et al., 2018 | 20 amateur soccer players, F, (20.6 ± 3.9 y) | To evaluate the effect of compression stocking use during an amateur female soccer match on match-induced fatigue indicators | 48 h before match: PR; Pre-match: PR, PCT;  post-match: PCT, RPE | Soccer match | During | NS | No effect |
| Pearce et al., 2009 | 8 participants, M, (23-27 y) | To examine if sports compression garments assist in the performance of a visuomotor tracking task following a bout of eccentric exercise, resulting in DOMS, at intervals up to 14 days post-exercise | MS | 35 maximal isokinetic eccentric extensions at 90˚/s | During | NS | No effect |
| Pereira et al., 2014 | 22 resistance trained participants, M, (24.6 ± 5.1 y) | To examine the effect of graduated compression sleeves worn during exercise on muscle recovery in young resistance trained men. | MS (before, 24, 48, 72, 96 h post-exercise) | 4 sets of 10 maximal elbow flexion/extension at 120˚/s. 1 min separated sets | During | NS | No effect |
| Perrey et al., 2008 | 8 participants, M, (26 ± 4 y) | To assess the adaptation response to DOMS while wearing graduated compression stockings | MS | Subjects exercised by walking on a motorized treadmill for 30-min at a constant velocity of 1m^.^s^-1^ with a negative grade of -25% and a load of 12% of body mass | Graduated compression stockings were worn 5 h per day at 2 h, 24 h, 48 h and 72 h | NS | Compression may attenuate soreness associated with delayed onset muscle soreness |
| Priego et al., 2015 | 20 recreational runners,  13M and 7F,  (28.1 ± 5.4 y) | To analyse the effects of running with and without GCS for three weeks on cardiorespiratory parameters in runners | PF | Running test: 30 minutes at 80% of MAS | During | Ankle: 24 mmHg and calf: 21 mmHg | No effect was observed after three weeks of running with GCS |
| Priego Quesada et al., 2015 | 44 runners, 29M and 15F, (29.3 ± 5.8 y) | To analyse the effects of running in a moderate environment with and without GCS on skin temperature in runners. | PF | 20min at 75% MAS | During | Ankle: 20 – 25 mmHg and knee: 15 – 10 mmHg | Running with graduated compression stockings did not influence perception of fatigue |
| Pruscino et al., 2013 | 8 highly trained hockey players, M, (21.9 ± 2.3 y) | To investigate the efficacy of wearing a full-length, lower-body compression garment following a hockey-simulation and post-exercise biochemical response and recovery of muscle function | RPE (following each 15min bout). PR (1, 24 & 48 h), MS | Hockey simulation protocol | 24 hours after | Ankle: 19.1 mmHg, calf: 7.2 mmHg, thigh: 4.9 mmHg. | Improved muscle soreness and perceived recovery |
| Ravier et al., 2018 | 18 handball players, M, (23.22 ± 4.97 y) | To investigate benefits of wearing full-leg length compression garments during conventional handball-specific circuit exercise on maximal and rapid muscle force characteristics immediately at the end and 24 hours post-exercise. | PP | Handball-specific circuit exercise during three 12-min periods. Exercise was separated by four minutes of rest. | During exercise | Ankle: 15, *medial gastrocnemius*: 27, and *vastus lateralis*: 14 mmHg | No effect |
| Rider et al., 2014 | 10 runners,  3F (18.7 ± 0.6 y) and 7M (21 ± 1.3 y) | To determine whether wearing below-the-knee graduated compression stockings e during a maximal treadmill run would induce physiological changes among collegiate cross-country runners. | RPE | 5-km running time trial | During | Ankle: 20 mmHg, Calf: 15 mmHg | No effect |
| Rimaud et al., 2010 | 8 endurance trained participants, M, (27.1 ± 0.9 y) | To investigate effects of wearing compression stockings during exercise | RPE | Volitional test to exhaustion | During and 60 min after | Maximum of 22 mmHg at the calf and 12 mmHg at the ankle | No significant difference in RPE |
| Rivas et al., 2016 | 13 endurance trained runners,  10M and 3F, (20.9 ± 2.5 y) | To determine if commercially available below the knee lower leg compressions would improve resting/submaximal/maximal exercise test cardiorespiratory measurements (heart rate, breathing rate, ventilation, oxygen uptake), lactate metabolism, and perception of exercise intensity during running in endurance-trained athletes | RPE | The incremental graded exercise test consisted of baseline rest and submaximal intensities at 23%, 70%, 75%, 85% and then a progressive increase to 100% VO_2max_ | During | Ankle: 12 – 15 mmHg, Calf: 9 – 12 mmHg | The results of this investigation demonstrate perception of effort at submaximal and maximal exercise capacity was not affected by lower leg compressions |
| Rugg & Sternlicht 2013 | 14 healthy participants, M, (28.2 ± 14.0 y) | To investigate if wearing graduated compression tights, compared with loose fitting running shorts, improves and sustain CMJ height after submaximal endurance running | RPE after 15mins of running, PC | 15 minutes of continuous running with 5 minutes performed at each of the following intensities: 50%, 70%, and 85% of heart rate reserve | During | Ankle: 18.0 mmHg, Calf: 12.6 mmHg, Thigh: 7.2 mmHg | Subjects reported significantly lower levels of perceived exertion and greater comfort values while wearing the graduated compression tights. |
| Santos Cerqueira et al., 2014 | 13 untrained participants, M, Compression group (22 ± 1 y),  Control group  (20 ± 1 y) | To assess the efficacy of compression sleeves worn12h, on recovery from the symptoms of exercise induced muscle damage in the upper arm muscles | MS | 30 repetitions of eccentric dumbbell curls which lasted 4-5 seconds | 12 hours following | NS | No effect |
| Smale et al., 2017 | 15 well-trained cyclists, M, (28.1 ± 6.3 y) | Examine the effects of varying grades of compression garments during incremental cycling exercise on cerebral artery blood flow velocity and cognitive performance | Stroop task. RPE, motivation, and perceived difficulty | Four 8 min increments of cycling at 30%, 50%, 70%, and 85% maximal power output and a 4 km time-trial. | During | Medium-grade garment at the ankle: 21.8 ± 6.6 mmHg, knee: 20.3 ± 6.6 mmHg, and thigh: 15.4 ± 4.5 mmHg  Low grade compression: ankle: 8.6 ± 2.7 mmHg, knee: 14.9 ± 4.9 mmHg, and thigh: 9.1 ± 3.1 mmHg | Compression garments may provide an advantage for cognitive performance during higher intensity exercise.  No effect on RPE, motivation and perceived difficulty. |
| Sperlich et al., 2010 | 15 healthy runners and triathletes, M, (27.1 ± 4.8 y) | To compare effects of three types of compression clothing (socks, tights, and whole-body) in well-trained athletes on s physiological responses and performance | RPE, MS | Sub-maximal running speed for 15 min. Thereafter, running speed was set at the highest speed achieved during incremental testing. | During | Aimed for 20 mmHg | No differences between compression clothing and clothing without external pressure. |
| Sperlich et al., 2013 | 10 well-trained endurance athletes, M, (25 ± 4 y) | To assess whether upper body compression garments improve double-poling sprint performance by measuring power output and selected metabolic, cardio-respiratory, hemodynamic and perceptual parameters | RPE | Three 3-min simulated double polling sprints on a cross-country ski ergometer | During | Forearm: 21 ± 5 mmHg  m. *triceps brachii*: 14 ± 3 mmHg  m. *biceps brachii*: 14 ± 2 mmHg  m. *latissimus dorsi*: 9 ± 2 mmHg | No effect |
| Terbizan et al., 2018 | 30 participants, M,  Control (21.56 ± 2.55 y),  Knee high stockings (21.80 ± 2.53 y),  Waist high tights (20.91 ± 1.92 y) | To compare the effects of waist-high compression garments and knee-high compression stockings for recovery from plyometric box drops. | MS ratings at 24-, 48-, and 72-h post-exercise | 10 sets of 10 plyometric box drop jumps from 60cm box. Up to ten seconds were allowed between drops, and one minute between sets. | Worn for 12 hours post-exercise | NS | No effect |
| Trenell et al., 2006 | 11 recreational athletes, M, (21.2 ± 3.1 y) | To observe the effects of 30-min of downhill walking on muscle metabolism and DOMS. The effect of compression garments applied after eccentric exercise was also studied. | MS | Participants performed a downhill walking protocol for 30 minutes on a treadmill (6 km·h^-1^, 25% grade). | 48 hours following exercise | Calf: 16 - 17 mmHg, thigh: 10 mmHg | No effect |
| Treseler et al., 2016 | 19 physically active participants,  F, (20 ± 1 y) | To examine the physiological and perceptual responses to wearing below-the-knee compression stockings after a 5-km running test in recreationally active women | MS for lower extremity and calf at 30 min and 24 h after exercise. PC | 5km time trial | During | 18 – 21 mmHg of pressure around the ankles, and 12.6 – 14.7 mmHg below the knee | Lower MS was observed in the compression trial |
| Upton et al., 2017 | 19 club level rugby union players, M, (20.3 ± 1.7 y) | To evaluate the efficacy of compression garments for the recovery of strength, power and indices of muscle damage from a rugby specific, muscle damaging protocol. | MS | Twenty 20m maximal sprints with 10m deceleration. | 48 hours post-exercise | Calf: 14 ± 4.1 mmHg, Thigh: 8.5 ± 2.3 mmHg | Perceived muscle soreness was significantly lower in the compression group compared to sham at both 24 and 48 h post exercise. |
| Varela-Sanz et al., 2011 | 16 well-trained runners, 3F (32.00 ± 4.58 y) and 13M (35.41 ± 6.61 y) | To assess the influence of below knee compression stockings on running economy and performance at competitive velocities | RPE | Experiment 1: (4 x 6min at 1/2 marathon pace)  Experiment 2: running as long as possible on a treadmill at a gradient of 1% and at a speed of 105% of the athlete’s recent 10-km time (average speed of 17 6 2 kmh21) until exhaustion. | During | NS | No effect |
| Venckunas et al., 2014 | 13 active participants, F, (25.1 ± 4.2 y) | To evaluate the effect of lower body compression garments on the cardiovascular function in response to a running session in a thermoneutral environment | Ratings of TS, shivering/sweating sensation and RPE | 4 km was covered in 30 min and 400m sprint | During | Thigh: ~17mmHg, upper calf: ~19mmHg | No significant differences were found for sensations both at rest and in response to running sessions. |
| Vercruyssen et al., 2012 | 11 trained runners, M, (34.7 ± 9.8 y) | To investigate the effects of wearing compression socks on performance indicators and physiological responses during prolonged trail running | RPE | 15.6 km trail run | During | 18 mmHg applied to the calf | No effect on RPE |
| Vercruyssen et al., 2016 | 12 runners, M, (39.6 ± 4.6 y) | To examine the influence of wearing compression garment vs. conventional running clothing on muscle contractile function and running economy before and after a short distance trail running. | RPE central and RPE peripheral | 18.4-km short distance trail runs | During | Ankle: 18 mmHg, Calf: 13 mmHg, short-tight: 7.5 mmHg | No effect |
| Webb & Willems, 2010 | 18 participants, M, (20 ± 1 y) | Examine the effect of wearing lower body compression garments during downhill running on recovery of jump height and delayed onset muscle soreness | MS | 5 x 8 minute bouts of downhill running performed at 80% of VO_2max_ at a -10% gradient. Two minute static recoveries followed each bout | During exercise | Calf: 18 mmHg, Thigh: 9 mmHg | Decreased perceived MS |
| Zinner et al., 2017 | 12 handball players, M, (22 ± 4 y) | To investigate the effects of increasing the level of compression on recovery following repeated sprints. | SARS pre-, post-, 24h, and 48h | 30 x 30m sprints | 48 hours post exercise | From below the hip to the foot exerting  mean pressures applied of 3 ± 1 mmHg, 11 ± 1 mmHg, 23 ± 2 mmHg | No effect |

CG= Compression Garment, GCS= Graduated compression stockings, CS= Compression Sleeve, RPE= Rating of Perceived Exertion, AFL = Australian Football League, VAS= Visual Analogue Scale, MS= Muscle soreness, MP= Muscle Pain, NS = Not stated, PR= Perceived Recovery, PF= Perceived Fatigue, PC= Perception of comfort, PCT= Perceived Comfort and Tightness, PEPS= Perceived Physical State Scale, PPO = Peak power output, MAS = maximal aerobic speed, WAnT = Wingate Anaerobic Test, TQR= Total Quality Recovery, PP= Perceived Pain, SARS= Acute Recovery and Stress Scale, TS= Thermal State, EIMD= Exercise induced muscle damage, VT1= ventilatory threshold one.
